# Discovery of Effective Inhibitors Against Phosphodiesterase 9, a Potential Therapeutic Target of Alzheimer’s Disease with Antioxidant Capacities

**DOI:** 10.3390/antiox14020123

**Published:** 2025-01-21

**Authors:** Qian Zhou, Xu-Nian Wu, Wei-Hao Luo, Qing-Hua Huang, Ling-Ling Feng, Yinuo Wu, Chen Zhang

**Affiliations:** 1 School of Chemistry and Chemical Engineering, Guangdong Pharmaceutical University, Zhongshan 528458, China; zhouq_47@163.com (Q.Z.); 2112373045@stu.gdpu.edu.cn (W.-H.L.); 2 State Key Laboratory of Anti-Infective Drug Discovery and Development, School of Pharmaceutical Sciences, Sun Yat-sen University, Guangzhou 510006, China; wuxn3@mail2.sysu.edu.cn (X.-N.W.); huangqh68@mail2.sysu.edu.cn (Q.-H.H.); fenglling3@mail2.sysu.edu.cn (L.-L.F.)

**Keywords:** phosphodiesterase 9 (PDE9), antioxidant capacity, multitarget-directed ligands (MTDLs), Alzheimer’s disease (AD)

## Abstract

Alzheimer’s disease (AD) is a widely recognized type of dementia that leads to progressive cognitive decline and memory loss, affecting a significant number of people and their families worldwide. Given the multifactorial nature of AD, multitarget-directed ligands (MTDLs) hold promise in developing effective drugs for AD. Phosphodiesterase-9 (PDE9) is emerging as a promising target for AD therapy. In this study, by combining a PDE9 inhibitor C33 with the antioxidant melatonin, we designed and discovered a series of pyrazolopyrimidinone derivatives that simultaneously inhibit PDE9 and possess antioxidant activities. Molecular docking, together with dynamics simulations, were applied to accelerate compound design and reduce synthetic work. Four out of the 14 compounds were validated as effective PDE9 inhibitors with comparable antioxidant activity. Notably, compounds **17b** and **17d** demonstrated IC_50_ values of 91 and 89 nM against PDE9, respectively, with good antioxidant activities (ORAC (Trolox) of 2.00 and 2.60). This work provides a new approach for designing MTDLs for the treatment of AD and offers insights for further structural modifications of PDE9 inhibitors with antioxidant capacities.

## 1. Introduction

Dementia, the seventh leading cause of death around the world, results from a variety of diseases and injuries that affect the brain. Alzheimer’s disease (AD) is a widely recognized type of dementia, characterized by age-related neurodegenerative disorders that lead to progressive cognitive decline and memory loss [1,2]. AD may contribute to 60–70% of dementia cases. As the population ages, the prevalence of AD continues to rise, making it a major public health challenge. According to open data from the World Health Organization (WHO), the number of people living with dementia worldwide is expected to increase from 55 million in 2019 to 139 million in 2050. The global costs associated with dementia are also expected to double, rising from $1.3 trillion per year in 2019 to $2.8 trillion by 2030 [3,4].

The pathogenesis of AD has been extensively studied for decades but remains unclear. It is generally believed that a combination of genetic, environmental, and biological factors leads to the progressive degeneration of brain cells in AD. Multiple biological factors, such as decreased acetylcholine levels, β-amyloid (Aβ) deposition, Tau-protein aggregation, and oxidative stress, have been judged important contributors to the pathophysiology of AD [4]. Unfortunately, there is no proven way to prevent or cure AD. Classical single-target drugs such as donepezil, rivastigmine, and memantine could only improve symptoms but not reverse the progression of AD patients [5]. Aducanumab and lecanemab are novel anti-AD biologics targeting and removing amyloid-beta (Aβ) plaques from the brain. However, these biologics are costly and have been subject to controversy regarding their efficacy and side effects [6,7].

Multitarget-directed ligands (MTDLs) are a class of compounds specifically designed to interact with multiple biological targets simultaneously. Given the multifactorial nature of AD, developing MTDLs for AD offers a promising alternative approach that has garnered significant attention from scientists [8,9]. Several MTDLs have progressed to clinical trials for treating AD, such as tramiprosate, which targets Aβ oligomers and Tau-protein aggregation simultaneously, ladostigil, a dual inhibitor against cholinesterase and monoamine oxidase, and blarcamesine, which functions as a sigma-1 receptor agonist and muscarinic receptor modulator [10].

Oxidative stress occurs when there is an imbalance between the production of reactive oxygen/nitrogen species (ROS/RNS) and the body’s ability to neutralize them with antioxidants. Due to their high metabolic activity, brain cells are particularly susceptible to oxidative damage, and this oxidative stress is believed to be a significant contributor to the progressive degeneration of brains in AD [11,12]. The antioxidant melatonin has been reported to reduce the levels of hydroxyl radicals in mitochondria, protect antioxidant enzyme activities, and prevent apoptosis of brain cells. However, there is currently insufficient evidence to substantiate the efficacy of any single antioxidant remedy in controlling the progression of AD in clinical research [13,14].

**Figure 1:**
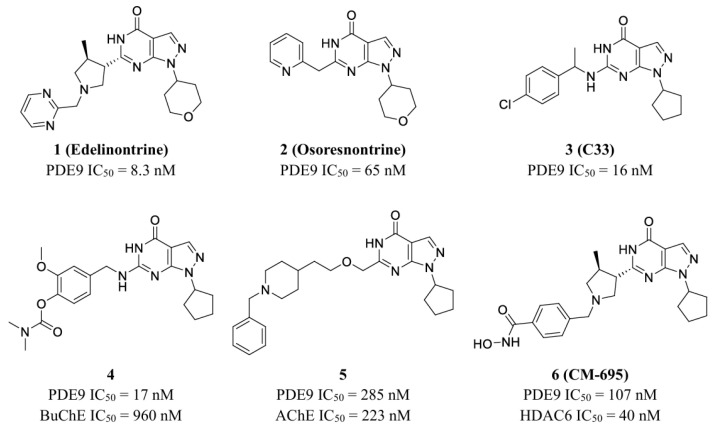
Current PDE9 inhibitors for research in AD.

Phosphodiesterases (PDEs) represent a superfamily of enzymes that are responsible for the degradation of the second messengers-cyclic nucleotides [15,16]. Among them, PDE9 has the highest affinity for cyclic guanosine monophosphate (cGMP) and is widely distributed in the cortex, basal ganglia, hippocampus, and cerebellum of the brain [17]. Human PDE9 is encoded by a single gene that undergoes alternative splicing, resulting in the production of at least four isoforms (PDE9A1-PDE9A4) [18]. PDE9A is localized to the membrane in different organs except in the bladder, where it is found in the cytosol. PDE9A1 is the longest variant with restricted localization in the nucleus [19,20]. In humans, particularly in elderly individuals with dementia and a history of traumatic brain injury, the mRNA for PDE9A in the hippocampus was found to be elevated [19]. PDE9 inhibitors block the degradation of cGMP and amplify NO-cGMP-signaling, leading to enhancement in synaptic plasticity and long-term potentiation (LTP). Considering that PDE9 is specifically localized to regulate the nuclear and membrane-proximal pools of cGMP, inhibition of PDE9 for the enhancement of cGMP signaling in the brain could be a promising new therapeutic method for AD [21]. Additionally, PDE9 has been shown to regulate a cGMP pool that is independent of neuronal nitric oxide synthase (nNOS), which is involved in nitric oxide-cGMP signaling [22]. This indicates a novel mechanism for regulating cGMP levels, distinct from those of other PDEs. Edelinontrine and osoresnontrine, two PDE9 inhibitors approved for clinical trials, have shown positive cognitive effects in numerous preclinical studies [23]. For example, edelinontrine could increase rat cerebrospinal fluid (CSF) cGMP levels dose-dependently and improve memory performances in the social recognition tasks, object recognition tasks, and Morris water maze. In the amyloid precursor protein (APP) transgenic Tg2576 mice, edelinontrine is capable of regulating dendritic spine density in hippocampal neurons [24]. PDE9A inhibition with osoresnontrine increases cGMP levels in the brain, facilitates synaptic plasticity by enhancing both early and late LTP in hippocampus, and improves performance on cognitive tasks assessing working and episodic memory in rodents [25]. This differs from AChE inhibitors such as donepezil, which could only enhance short memory in the early LTP [26]. Thus, PDE9 inhibitors provide a novel means for the treatment of AD. Several MTDLs targeting PDE9 and other enzymes such as cholinesterases (AChE and BuChE) and histone deacetylases (HDACs) were also reported for the treatment of AD in recent years (Figure 1) [27,28].

Since oxidative stress and impaired neuronal signaling are two major contributors to AD pathology, combining PDE9 inhibitors with antioxidants may offer synergistic benefits in treating AD. However, there are few reports on the combined use of PDE9 inhibitors with antioxidants or on the development of MTDLs that simultaneously target PDE9 and oxidative stress. With our continuous interest in designing MTDLs for AD [29,30], herein, we report the design and discovery of a series of pyrazolopyrimidinone derivatives inspired by the PDE9 inhibitor C33 developed by our group [31] and the antioxidant melatonin. These derivatives were evaluated as potent PDE9 inhibitors with good antioxidant activities comparable to melatonin, which will benefit AD drug development.

## 2. Materials and Methods

### 2.1. Chemistry

#### 2.1.1. General Remarks

All starting materials and reagents were purchased from commercial suppliers (Bide, Macklin, Aladdin, and Meryer) without further purification. For chromatography, chemical HG/T2354-92 silica gel (200−300 mesh, Haiyang) was used, and for the thin-layer chromatography analysis, silica gel plates with fluorescence F254 (0.25 mm, Huanghai) were used. NMR spectra were recorded at room temperature using either a Bruker Ascend TM 500 (Bruker, Berlin, Germany) or a Bruker Avance III (Bruker, Zurich, Switzerland). The following abbreviations are applied: s (singlet), br (broad signal), d (doublet), dd (doublet of doublets), t (triplet), q (quartet), and m (multiplet). Coupling constants are reported in hertz (Hz). The high-resolution mass spectra (HRMS) (SHIMADZU, Osaka, Japan) were recorded on a MAT-95. HPLC instrument (SHIMADZU, Osaka, Japan): SHIMADZU LC20AT [column: Hypersil BDS C18, 5.0 μm, 4.6 × 150 mm (Elite)]; detector: SPD-20A UV/vis detector, UV detection at 254 nm; elution, MeOH in water (80–100%, *v*/*v*); T = 25 °C; and flow rate = 1.0 mL/min.

#### 2.1.2. General Procedure for the Preparation of the Intermediates and Pyrazolopyrimidinone Derivatives

*4,6-dichloro-1-cyclopentyl-1H-pyrazolo [3,4-d]pyrimidine* (**8**). Cyclopentylhydrazine hydrochloride (**7**) (0.300 g, 2.2 mmol), 2,4,6-trichloropyrimidine-5-carbaldehyde (0.424 g, 2.0 mmol), and triethylamine (0.404 g, 4.0 mmol) were dissolved in ethanol (40 mL). The mixture was stirred at −78 °C for 2 h, heated to ambient temperature, and then stirred for another 8 h. After the reaction was finished, the mixture was diluted with water and extracted three times with ethyl acetate. The combined organic extracts were then dried over anhydrous Na_2_SO_4_ and concentrated to yield a crude product. This crude product was subsequently purified using silica gel column chromatography (DCM/MeOH) to afford intermediate **8** as a white solid (0.373 g, 73% yield). ^1^H NMR (400 MHz, CDCl_3_) δ 8.14 (s, 1H), 5.36–5.26 (m, 1H), 2.25–2.07 (m, 4H), 2.05–1.94 (m, 2H), 1.82–1.71 (m, 2H).

*6-chloro-1-cyclopentyl-1,5-dihydro-4H-pyrazolo [3,4-d]pyrimidin-4-one* (**9**). To a solution of sodium hydroxide in water (1 mol/L, 20 mL), intermediate **8** (0.257 g, 1 mmol) was added, and the mixture was stirred at 60 °C for 1 h. After the reaction was finished, the mixture was acidified with acetic acid to pH of 5–6. Then the solid was filtered, washed with water, and dried to get the white solid as intermediate **9** (0.410 g, 80% yield). ^1^H NMR (400 MHz, CDCl_3_) δ 8.10 (s, 1H), 5.15 (*p*, *J* = 7.5 Hz, 1H), 2.20–2.04 (m, 4H), 2.02–1.91 (m, 2H), 1.72 (ddd, *J* = 11.2, 7.8, 3.2 Hz, 2H).

*4,6-dichloro-1-(tetrahydro-2H-pyran-4-yl)-1H-pyrazolo[3,4-d]pyrimidine* (**11**). The title compound was synthesized from (tetrahydro-2H-pyran-4-yl)hydrazine hydrochloride (**10**) (0.727 g, 7.2 mmol) and **7** (0.763 g, 3.6 mmol) following the general procedure for the preparation of intermediate **8**, affording a white solid as intermediate **11** (0.324 g, 66% yield). ^1^H NMR (400 MHz, CDCl_3_) δ 8.16 (s, 1H), 5.14–4.89 (m, 1H), 4.16 (dd, *J* = 11.5, 4.6 Hz, 2H), 3.63 (td, *J* = 12.1, 2.0 Hz, 2H), 2.48–2.25 (m, 2H), 1.98–1.93 (m, 2H).

*6-chloro-1-(tetrahydro-2H-pyran-4-yl)-1,5-dihydro-4H-pyrazolo[3,4-d]pyrimidin-4-one* (**12**). The title compound was synthesized from intermediate **11** (0.273 g, 4.0 mmol) following the general procedure for the preparation of intermediate **11**, affording a white solid as intermediate **12** (0.209 g, 82% yield). ^1^H NMR (400 MHz, CDCl_3_) δ 8.13 (s, 1H), 4.89–4.83 (m, 1H), 4.19–4.15 (m, 2H), 3.66–3.59 (m, 2H), 2.44–2.34 (m, 2H), 1.99–1.92 (m, 2H).

*5-amino-1-cyclopentyl-1H-pyrazole-4-carbonitrile* (**13**). Cyclopentylhydrazine hydrochloride (**7**) (0.540 g, 4.0 mmol) was dissolved in ethanol (4 mL), followed by slow-paced addition of triethylamine (1.272 g, 12.6 mmol) at 0 °C. After stirring for 2 h, a solution of 2-(ethoxymethylene)malononitrile (439 mg, 3.6 mmol) in ethanol was added to the mixture dropwise. The mixture was then stirred at ambient temperature for 3 h, followed by refluxing at 80 °C for another 3 h. Upon completion of the reaction, water was added and the resulting precipitate formed. The brown precipitate was collected and then purified through recrystallization with the solvent composed of ether and hexane (1:1), affording intermediate **13** (0.539 g, 85% yield). ^1^H NMR (400 MHz, DMSO) d 8.11 (s, 1H), 5.51 (d, *J* = 6.8 Hz, 1H), 4.50–4.38 (m, 1H), 3.39 (s, 1H), 2.03–1.93 (m, 2H), 1.88–1.79 (m, 2H), 1.76–1.68 (m, 2H), 1.63–1.53 (m, 2H). ^13^C NMR (101 MHz, DMSO) d 157.33, 134.01, 114.78, 76.01, 62.18, 31.82, 23.64.

*5-amino-1-cyclopentyl-1H-pyrazole-4-carboxamide* (**14**). To a solution of intermediate **13** (0.528 g, 3.0 mmol) in ethanol (15 mL), 30% hydrogen peroxide (1.5 mL) and 25% aqueous ammonia (4.5 mL) were added sequentially. The mixture was stirred at ambient temperature for 1 h. Upon completion of the reaction, saturated sodium thiosulfate was added, and ethanol was evaporated to form an orange precipitate. The precipitate was filtered, washed with water, and dried under vacuum to afford intermediate **15** as a white solid (0.500 g, 86% yield). ^1^H NMR (400 MHz, DMSO) δ 7.63 (s, 1H), 7.16 (br s, 1H), 6.62 (br s, 1H), 6.13 (m, 1H), 4.57–4.45 (m, 1H), 3.39 (s, 1H), 2.00–1.87 (m, 2H), 1.87–1.72 (m, 4H), 1.63–1.50 (m, 2H). ^13^C NMR (101 MHz, DMSO) δ 165.73 (d, *J* = 0.8 Hz), 148.24 (t, *J* = 4.9 Hz), 136.27, 96.17 (d, *J* = 2.9 Hz), 55.30 (d, *J* = 1.5 Hz), 30.65, 23.48.

*benzyl (R)-(1-(1-cyclopentyl-4-oxo-4,5-dihydro-1H-pyrazolo[3,4-d]pyrimidin-6-yl)ethyl)carbamate* (**15**). To a solution of intermediate **14** (0.485 g, 2.5 mmol), (*R*)-ethyl-2-(((benzyloxy)carbonyl)amino)propanoate (2.5 g, 10 mmol) in anhydrous THF (10 mL), sodium hydride (60% dispersion in mineral oil, 0.400 g, 10 mmol) was added. The mixture was stirred overnight at ambient temperature. Then, water was added slowly to the mixture to quench the reaction. The mixture was then extracted with ethyl acetate, washed with brine, and dried over Na_2_SO_4_. After concentration, the residue was purified by silica gel chromatography (MeOH/CH_2_Cl_2_ 1:100), yielding intermediate **15** in the form of a yellow oil (400 mg, 42% yield). ^1^H NMR (400 MHz, CDCl_3_) d 11.90 (s, 1H), 8.05 (s, 1H), 7.32–7.28 (m, 5H), 5.98 (d, *J* = 6.1 Hz, 1H), 5.23–5.05 (m, 3H), 4.98–4.83 (m, 1H), 2.10 (dd, *J* = 13.6, 6.9 Hz, 4H), 2.02–1.92 (m, 2H), 1.79–1.69 (m, 2H), 1.60 (d, *J* = 7.1 Hz, 3H). ^13^C NMR (101 MHz, CDCl_3_) d 160.26, 159.79, 155.83, 151.90, 136.12, 134.56, 128.50, 128.18, 104.41, 67.18, 57.89, 50.43, 32.47, 24.78, 20.56.

*(R)-6-(1-aminoethyl)-1-cyclopentyl-1,5-dihydro-4H-pyrazolo[3,4-d]pyrimidin-4-one* (**16**). To a solution of intermediate **15** (0.381 g, 1 mmol) in methanol, Pd/C (10%, 0.038 g) as a catalyst was added. The mixture was stirred at ambient temperature for 24 h under an atmospheric hydrogen pressure. The catalyst was filtered off, and the filtrate was concentrated. The residue was purified by silica gel chromatography (MeOH/CH_2_Cl_2_ 1:50) to afford intermediate **16** as a white solid (128 mg, 52%). ^1^H NMR (400 MHz, CDCl_3_) δ 8.05 (s, 1H), 5.22–5.08 (m, 1H), 4.12 (q, *J* = 6.8 Hz, 1H), 2.20–2.03 (m, 4H), 2.01–1.91 (m, 2H), 1.76–1.67 (m, 2H), 1.53 (d, *J* = 6.9 Hz, 3H). ^13^C NMR (101 MHz, CDCl_3_) δ 162.65, 158.53, 152.48, 134.49, 104.72, 57.81, 49.70, 32.44, 32.32, 24.75, 23.15.

*6-((1-(1H-indol-3-yl)propan-2-yl)amino)-1-cyclopentyl-1,5-dihydro-4H-pyrazolo[3,4-d]pyrimidin-4-one* (**17a**). To a solution of intermediate **9** (48 mg, 0.20 mmol) in isopropanol (3 mL), 1-(1*H*-indol-3-yl)propan-2-amine (43 mg, 0.24 mmol), and triethylamine (40 mg, 0.40 mmol) were added. The mixture was refluxed overnight at 90 °C. After the reaction was finished, the mixture was cooled to ambient temperature. The solution was evaporated in a vacuum and the residue was purified by silica gel chromatography (MeOH/CH_2_Cl_2_ 1:50) to afford **17a** as a white solid (40 mg, 52% yield). ^1^H NMR (400 MHz, CDCl_3_) δ 8.08 (s, 1H), 7.77 (d, *J* = 9.6 Hz, 2H), 7.32 (d, *J* = 8.1 Hz, 1H), 7.17 (t, *J* = 7.4 Hz, 1H), 7.10 (m, 2H), 5.90 (d, *J* = 7.5 Hz, 1H), 5.11 (m, 1H), 4.51 (m, 1H), 3.21 (dd, *J* = 13.9, 4.8 Hz, 1H), 2.93 (dd, *J* = 14.1, 7.5 Hz, 1H), 2.12 (m, 4H), 1.97 (dt, *J* = 12.7, 7.4 Hz, 2H), 1.74 (m, 2H), 1.30 (d, *J* = 6.5 Hz, 3H). ^13^C NMR (101 MHz, Acetone) δ 157.95, 154.18, 152.75, 136.83, 133.73, 128.04, 123.43, 121.25, 118.72, 118.46, 111.57, 111.36, 100.08, 57.09, 47.18, 31.97, 31.80, 31.78, 24.49, 24.48, 19.37. HRMS (ESI-TOF) *m*/*z* [M + H]^+^ calcd for C_21_H_24_N_6_O 377.2084, found 377.2074.

*1-cyclopentyl-6-((2-(5-hydroxy-1H-indol-3-yl)ethyl)amino)-1,5-dihydro-4H-pyrazolo[3,4-d]pyrimidin-4-one* (**17b**). The title compound was synthesized from intermediate **9** (48 mg, 0.20 mmol) and 5-hydroxytryptamine hydrochloride (51 mg, 0.24 mmol) following the general procedure for the preparation of **17a**, affording a white solid as the final product (51 mg, 67% yield). Purity = 96%. ^1^H NMR (400 MHz, MeOD) δ 7.79 (s, 1H), 7.16 (d, *J* = 8.6 Hz, 1H), 7.03 (s, 1H), 7.00 (d, *J* = 2.1 Hz, 1H), 6.67 (dd, *J* = 8.6, 2.2 Hz, 1H), 5.05 (m, 1H), 3.71 (t, *J* = 6.9 Hz, 2H), 3.00 (t, *J* = 6.9 Hz, 2H), 2.10 (m, 2H), 2.00 (m, 2H), 1.93 (m, 2H), 1.72 (m, 2H). ^13^C NMR (101 MHz, MeOD) δ 160.01, 154.46, 153.28, 149.81, 133.93, 131.80, 128.10, 123.09, 111.28, 111.00, 110.79, 102.23, 99.40, 57.38, 41.04, 31.41, 24.89, 24.29. HRMS (ESI-TOF) *m*/*z* [M + H]^+^ calcd for C_20_H_22_N_6_O_2_ 379.1877, found 379.1884.

*1-cyclopentyl-6-((1-(5-methoxy-1H-indol-3-yl)propan-2-yl)amino)-1,5-dihydro-4H-pyrazolo[3,4-d]pyrimidin-4-one* (**17c**). The title compound was synthesized from intermediate **9** (48 mg, 0.20 mmol) and 5-methoxy-α-methyltryptamine (51 mg, 0.24 mmol) following the general procedure for the preparation of **17a**, affording a white solid as the final product (53 mg, 66% yield). ^1^H NMR (400 MHz, CDCl_3_) δ 10.70 (s, 1H), 8.09 (s, 1H), 7.67 (s, 1H), 7.15 (d, *J* = 8.8 Hz, 1H), 7.10 (d, *J* = 2.1 Hz, 1H), 7.05 (d, *J* = 1.6 Hz, 1H), 6.79 (dd, *J* = 8.7, 2.3 Hz, 1H), 6.27 (d, *J* = 7.6 Hz, 1H), 5.10 (dd, *J* = 15.0, 7.5 Hz, 1H), 4.56 (dt, *J* = 13.1, 6.6 Hz, 1H), 3.81 (s, 3H), 3.06 (qd, *J* = 14.4, 6.0 Hz, 2H), 2.12 (m, 3H), 2.00 (m, 3H), 1.74 (m, 2H), 1.33 (d, *J* = 6.6 Hz, 3H). ^13^C NMR (101 MHz, CDCl_3_) δ 160.29, 154.66, 153.89, 152.40, 133.94, 131.49, 128.32, 123.85, 111.70, 111.65, 111.58, 101.44, 99.64, 57.43, 55.91, 46.94, 32.07, 31.95, 24.86, 20.34. HRMS (ESI-TOF) *m*/*z* [M + H]^+^ calcd for C_22_H_26_N_6_O_2_ 407.2190, found 407.2197.

*6-((1-(5-methoxy-1H-indol-3-yl)propan-2-yl)amino)-1-(tetrahydro-2H-pyran-4-yl)-1,5-dihydro-4H-pyrazolo[3,4-d]pyrimidin-4-one* (**17d**). The title compound was synthesized from intermediate **12** (50 mg, 0.20 mmol) and 5-methoxy-α-methyltryptamine (51 mg, 0.24 mmol) following the general procedure for the preparation of **17a**, affording a white solid as the final product (42 mg, 50% yield). Purity = 99%. ^1^H NMR (400 MHz, MeOD) δ 7.77 (s, 1H), 7.19 (d, *J* = 8.8 Hz, 1H), 7.04 (d, *J* = 2.1 Hz, 2H), 6.75 (s, 1H), 4.60 (m, 1H), 4.47 (dd, *J* = 12.9, 6.4 Hz, 1H), 4.04 (td, *J* = 11.5, 3.7 Hz, 2H), 3.74 (s, 3H), 3.58 (dd, *J* = 12.1, 1.8 Hz, 2H), 3.00 (ddd, *J* = 33.7, 14.3, 6.2 Hz, 2H), 2.21 (m, 2H), 1.79 (m, 2H), 1.28 (d, *J* = 6.7 Hz, 3H). ^13^C NMR (101 MHz, MeOD) δ 160.47, 154.24, 153.50, 152.82, 134.05, 132.01, 128.26, 123.79, 111.41, 110.67, 110.61, 100.58, 99.99, 66.62, 66.60, 54.85, 53.12, 31.56, 31.53, 19.10. HRMS (ESI-TOF) *m*/*z* [M + H]^+^ calcd for C_22_H_26_N_6_O_3_ 423.2139, found 423.2147.

*6-((4-(1H-indol-3-yl)butan-2-yl)amino)-1-cyclopentyl-1,5-dihydro-4H-pyrazolo[3,4-d]pyrimidin-4-one* (**17e**). The title compound was synthesized from intermediate **9** (48 mg, 0.20 mmol) and 4-(1*H*-indol-3-yl)butan-2-amine (47 mg, 0.24 mmol) following the general procedure for the preparation of **17a**, affording a white solid as the final product (40 mg, 50% yield). ^1^H NMR (400 MHz, MeOD) δ 7.80 (s, 1H), 7.49 (d, *J* = 7.9 Hz, 1H), 7.32 (d, *J* = 8.1 Hz, 1H), 7.06 (t, *J* = 7.2 Hz, 1H), 7.02 (s, 1H), 6.93 (t, *J* = 7.2 Hz, 1H), 4.93 (m, 1H), 4.17 (dd, *J* = 13.3, 6.6 Hz, 1H), 2.87 (t, *J* = 7.5 Hz, 2H), 1.99 (dt, *J* = 20.7, 7.1 Hz, 6H), 1.90 (m, 2H), 1.68 (m, 2H), 1.30 (d, *J* = 6.5 Hz, 3H). ^13^C NMR (101 MHz, MeOD) δ 159.98, 154.39, 152.68, 136.79, 133.87, 127.32, 121.39, 120.78, 117.95, 117.84, 114.29, 110.79, 99.41, 57.62, 46.36, 36.87, 31.35, 31.26, 24.30, 21.33, 19.48. HRMS (ESI-TOF) *m*/*z* [M + H]^+^ calcd for C_22_H_26_N_6_O 391.2241, found 391.2248.

*1-cyclopentyl-6-((1-(indolin-3-yl)propan-2-yl)amino)-1,5-dihydro-4H-pyrazolo[3,4-d]pyrimidin-4-one* (**17f**). The title compound was synthesized from intermediate **9** (48 mg, 0.20 mmol) and 1-(indolin-3-yl)propan-2-amine (42 mg, 0.55 mmol) following the general procedure for the preparation of **17a**, affording a white solid as the final product (34 mg, 47% yield). ^1^H NMR (400 MHz, MeOD) δ 7.86 (d, *J* = 4.6 Hz, 1H), 7.23 (dd, *J* = 41.2, 7.4 Hz, 1H), 7.05 (t, *J* = 7.4 Hz, 1H), 6.76 (m, 2H), 5.07 (m, 1H), 4.41 (m, 1H), 3.72 (dt, *J* = 14.1, 8.6 Hz, 1H), 3.42 (dd, *J* = 13.2, 5.6 Hz, 1H), 3.29 (ddd, *J* = 8.9, 7.0, 3.7 Hz, 1H), 2.16 (m, 2H), 2.09 (m, 2H), 2.02 (m, 2H), 1.78 (m, 2H), 1.37 (dd, *J* = 12.8, 6.6 Hz, 3H). ^13^C NMR (101 MHz, MeOD) δ 162.35, 156.85, 156.81, 155.27, 155.16, 153.70, 153.65, 136.44, 136.42, 135.57, 135.47, 129.63, 129.56, 126.18, 125.67, 121.29, 121.11, 112.73, 112.67, 101.97, 101.88, 60.10, 59.80, 55.84, 55.68, 47.68, 47.38, 43.99,43.71, 41.80, 41.64, 33.96, 33.95, 33.84, 33.70, 26.82, 26.80, 26.74, 26.71, 22.58, 22.43. HRMS (ESI-TOF) *m*/*z* [M + H]^+^ calcd for C_21_H_26_N_6_O 379.2241, found 379.2232.

*6-((1-(1H-indazol-3-yl)propan-2-yl)amino)-1-cyclopentyl-1,5-dihydro-4H-pyrazolo[3,4-d]pyrimidin-4-one* (**17g**). The title compound was synthesized from intermediate **9** (48 mg, 0.20 mmol) and 1-(1*H*-indazol-3-yl)propan-2-amine (42 mg, 0.36 mmol) following the general procedure for the preparation of **17a**, affording a white solid as the final product (45 mg, 60% yield). ^1^H NMR (400 MHz, MeOD) δ 7.82 (d, *J* = 8.1 Hz, 1H), 7.76 (s, 1H), 7.45 (d, *J* = 8.4 Hz, 1H), 7.35 (t, *J* = 7.5 Hz, 1H), 7.10 (t, *J* = 7.4 Hz, 1H), 4.90 (m, 1H), 4.61 (dd, *J* = 13.0, 6.5 Hz, 1H), 3.29 (d, *J* = 6.2 Hz, 1H), 3.22 (m, 1H), 2.01 (m, 4H), 1.92 (m, 2H), 1.69 (m, 2H), 1.29 (d, *J* = 6.6 Hz, 3H). ^13^C NMR (101 MHz, MeOD) δ 159.84, 154.20, 152.57, 142.99, 141.20, 133.91, 126.46, 122.14, 119.83, 119.68, 109.85, 99.38, 57.29, 46.58, 33.29, 31.40, 31.23, 24.18, 24.15, 19.08. HRMS (ESI-TOF) *m*/*z* [M + H]^+^ calcd for C_20_H_23_N_7_O 378.2037, found 378.2026.

*1-cyclopentyl-6-((1-(5-methoxy-1H-indazol-3-yl)propan-2-yl)amino)-1,5-dihydro-4H-pyrazolo[3,4-d]pyrimidin-4-one* (**17h**). The title compound was synthesized from intermediate **9** (48 mg, 0.20 mmol) and 1-(5-methoxy-1*H*-indazol-3-yl)propan-2-amine (48 mg, 0.36 mmol) following the general procedure for the preparation of **17a**, affording a white solid as the final product (56 mg, 46% yield). ^1^H NMR (400 MHz, MeOD) δ 7.75 (s, 1H), 7.33 (dd, *J* = 9.0, 0.5 Hz, 1H), 7.07 (d, *J* = 2.0 Hz, 1H), 7.01 (dd, *J* = 9.0, 2.3 Hz, 1H), 4.89 (m, 1H), 4.59 (dt, *J* = 11.8, 5.9 Hz, 1H), 3.74 (s, 3H), 3.32 (dd, *J* = 15.1, 5.0 Hz, 1H), 3.17 (dd, *J* = 14.2, 6.4 Hz, 1H), 1.99 (m, 4H), 1.91 (ddd, *J* = 11.4, 5.8, 3.4 Hz, 2H), 1.68 (m, 2H), 1.32 (d, *J* = 6.7 Hz, 3H). ^13^C NMR (101 MHz, MeOD) δ 159.75, 154.46, 154.22, 152.52, 142.10, 137.08, 133.83, 122.53, 118.53, 110.79, 99.39, 99.04, 57.36, 54.69, 46.51, 32.77, 31.43, 31.24, 24.21, 24.18, 19.20. HRMS (ESI-TOF) *m*/*z* [M + H]^+^ calcd for C_21_H_25_N_7_O_2_ 408.2142, found 408.2132.

*(R)-6-(1-(((1H-indol-3-yl)methyl)amino)ethyl)-1-cyclopentyl-1,5-dihydro-4H-pyrazolo[3,4-d]pyrimidin-4-one* (**18a**). To a solution of intermediate **16** (48 mg, 0.20 mmol), indole-3-carboxaldehyde (29 mg, 0.20 mmol), anhydrous sodium acetate (44 mg, 0.54 mmol) in isopropanol (3 mL), sodium cyanoborohydride (23 mg, 0.36 mmol) was added. The mixture was stirred for 16 h at ambient temperature. The solution was evaporated in a vacuum and the residue was dissolved in ethyl acetate. Then the solution was sequentially washed with a saturated solution of NaHCO_3_ and brine three times, dried over anhydrous Na_2_SO_4_, and purified by silica gel chromatography to afford **18a** as a white solid (15 mg, 19% yield). ^1^H NMR (400 MHz, CDCl_3_) δ 8.23 (s, 1H), 8.05 (s, 1H), 7.63 (d, *J* = 7.7 Hz, 1H), 7.36 (d, *J* = 8.0 Hz, 1H), 7.20 (t, *J* = 7.0 Hz, 1H), 7.15 (m, 2H), 5.17 (m, 1H), 3.95 (m, 3H), 2.11 (m, 4H), 1.98 (m, 2H), 1.72 (m, 2H), 1.44 (d, *J* = 6.9 Hz, 3H). ^13^C NMR (101 MHz, CDCl_3_) δ 161.90, 158.07, 152.48, 134.55, 126.65, 122.90, 122.43, 119.89, 118.43, 113.65, 111.36, 105.02, 99.98, 57.81, 56.46, 43.42, 32.47, 32.36, 24.75, 21.62. HRMS (ESI-TOF) *m*/*z* [M − H]^−^ calcd for C_21_H_24_N_6_O 375.1939, found 375.1934.

*(R)-1-cyclopentyl-6-(1-(((5-fluoro-1H-indol-3-yl)methyl)amino)ethyl)-1,5-dihydro-4H-pyrazolo[3,4-d]pyrimidin-4-one* (**18b**). The title compound was synthesized from intermediate **16** (48 mg, 0.20 mmol) and 5-fluoroindole-3-carboxaldehyde (31 mg, 0.20 mmol) following the general procedure for the preparation of **18a**, affording a white solid as the final product (39 mg, 49% yield). Purity = 96%. ^1^H NMR (400 MHz, CDCl_3_) δ 10.13 (s, 1H), 8.16 (s, 1H), 8.04 (s, 1H), 7.29 (d, *J* = 4.5 Hz, 1H), 7.25 (d, *J* = 2.2 Hz, 1H), 7.20 (d, *J* = 1.9 Hz, 1H), 6.95 (td, *J* = 9.0, 2.4 Hz, 1H), 5.17 (m, 1H), 3.91 (m, 3H), 2.12 (m, 4H), 1.98 (m, 2H), 1.72 (m, 2H), 1.44 (m, 3H). ^13^C NMR (101 MHz, CDCl_3_) δ 161.78, 158.11, 152.41, 134.51, 132.80, 124.68, 112.06, 111.97, 110.95, 110.69, 103.56, 103.32, 57.83, 56.57, 43.26, 32.44, 32.36, 24.74, 21.63. HRMS (ESI TOF) *m*/*z* [M − H]^−^ calcd for C_21_H_23_FN_6_O 393.1845, found 393.1836.

*(R)-1-cyclopentyl-6-(1-(((5-methoxy-1H-indol-3-yl)methyl)amino)ethyl)-1,5-dihydro-4H-pyrazolo[3,4-d]pyrimidin-4-one* (**18c**). To a solution of intermediate **16** (48 mg, 0.20 mmol), 5-methoxyindole-3-carboxaldehyde (32 mg, 0.20 mmol), anhydrous sodium acetate (44 mg, 0.54 mmol) in isopropanol (5 mL), sodium cyanoborohydride (23 mg, 0.36 mmol) was added. The title compound was synthesized according to the general procedure for the preparation of **18a**, affording a white solid as the final product (47 mg, 63% yield). ^1^H NMR (400 MHz, CDCl_3_) δ 10.20 (s, 1H), 8.05 (s, 1H), 8.01 (s, 1H), 7.26 (d, *J* = 8.5 Hz, 1H), 7.13 (s, 1H), 7.06 (s, 1H), 6.88 (d, *J* = 8.8 Hz, 1H), 5.17 (*p*, *J* = 7.4 Hz, 1H), 3.92 (m, 6H), 2.11 (td, *J* = 13.0, 6.5 Hz, 4H), 1.97 (m, 2H), 1.73 (m, 2H), 1.44 (m, 3H). ^13^C NMR (101 MHz, CDCl_3_) δ 161.89,157.90, 154.44, 152.52, 134.61, 131.45, 127.07, 123.62, 113.43, 112.81, 112.13, 105.07, 100.20, 57.75, 56.40, 55.99, 43.54, 32.47, 32.41, 24.77, 21.70. HRMS (ESI-TOF) *m*/*z* [M + H]^+^ calcd for C_22_H_26_N_6_O_2_ 407.2190, found 407.2180.

*(R)-1-cyclopentyl-6-(1-(((5-methoxy-1-methyl-1H-indol-3-yl)methyl)amino)ethyl)-1,5-dihydro-4H-pyrazolo[3,4-d]pyrimidin-4-one* (**18d**). The title compound was synthesized from intermediate **16** (48 mg, 0.20 mmol) and 5-methoxy-1-methylindole-3-carbaldehyde (38 mg, 0.20 mmol) following the general procedure for the preparation of **18a**, affording a white solid as the final product (55 mg, 65% yield). Purity = 91%. ^1^H NMR (400 MHz, CDCl_3_) δ 10.19 (s, 1H), 8.05 (s, 1H), 7.18 (d, *J* = 8.8 Hz, 1H), 7.04 (s, 1H), 6.97 (s, 1H), 6.90 (d, *J* = 8.8 Hz, 1H), 5.16 (m, 1H), 3.90 (m, 6H), 3.72 (s, 3H), 2.11 (m, 4H), 1.98 (m, 2H), 1.73 (m, 2H), 1.44 (d, *J* = 6.8 Hz, 3H). ^13^C NMR (101 MHz, CDCl_3_) δ 161.95, 157.90, 154.21, 134.53, 132.50, 128.24, 127.46, 112.27, 111.50, 110.24, 105.01, 100.41, 57.77, 56.18, 55.79, 43.35, 32.82, 32.45, 32.38, 29.68, 24.75, 21.61. HRMS (ESI-TOF) *m*/*z* [M − H]^−^ calcd for C_23_H_28_N_6_O_2_ 419.2201, found 419.2205.

*(R)-6-(1-(((1H-indazol-3-yl)methyl)amino)ethyl)-1-cyclopentyl-1,5-dihydro-4H-pyrazolo[3,4-d]pyrimidin-4-one* (**18e**). The title compound was synthesized from intermediate **16** (48 mg, 0.20 mmol) and 1*H*-indazole-3-aldehyde (30 mg, 0.20 mmol) following the general procedure for the preparation of **18a**, affording a white solid as the final product (32 mg, 41% yield). ^1^H NMR (400 MHz, CDCl_3_) δ 11.62 (s, 1H), 7.83 (s, 1H), 7.55 (d, *J* = 7.9 Hz, 1H), 7.35 (d, *J* = 8.4 Hz, 1H), 7.14 (t, *J* = 7.6 Hz, 1H), 6.91 (t, *J* = 7.2 Hz, 1H), 5.01 (dd, *J* = 13.8, 6.9 Hz, 1H), 4.39 (d, *J* = 14.8 Hz, 1H), 4.19 (d, *J* = 14.9 Hz, 1H), 3.95 (m, 1H), 2.06 (m, 4H), 1.97 (m, 2H), 1.73 (m, 2H), 1.45 (d, *J* = 6.4 Hz, 3H). ^13^C NMR (101 MHz, CDCl_3_) δ 162.57, 158.90, 151.56, 144.48, 140.71, 134.02, 126.79, 121.11, 120.58, 119.38, 110.28, 103.91, 58.26, 57.60, 44.46, 32.54, 32.27, 24.79, 21.66. HRMS (ESI-TOF) *m*/*z* [M − H]^−^ calcd for C_20_H_23_N_7_O 376.1891, found 376.1886.

*(R)-1-cyclopentyl-6-(1-(((5-methoxy-1H-indazol-3-yl)methyl)amino)ethyl)-1,5-dihydro-4H-pyrazolo[3,4-d]pyrimidin-4-one* (**18f**). The title compound was synthesized from intermediate **16** (48 mg, 0.20 mmol) and 5-methoxy-1*H*-indazole-3-carboxyaldehyde (35 mg, 0.20 mmol) following the general procedure for the preparation of **18a**, affording a white solid as the final product (40 mg, 49% yield). ^1^H NMR (400 MHz, CDCl_3_) δ 11.57 (s, 1H), 7.91 (s, 1H), 7.24 (d, *J* = 8.8 Hz, 1H), 6.89 (d, *J* = 8.8 Hz, 2H), 4.99 (dd, *J* = 13.7, 7.0 Hz, 1H), 4.30 (dd, *J* = 14.6, 3.7 Hz, 1H), 4.13 (d, *J* = 14.6 Hz, 1H), 3.94 (q, *J* = 6.7 Hz, 1H), 3.83 (s, 3H), 2.05 (m, 4H), 1.96 (m, 2H), 1.70 (m, 2H), 1.45 (dd, *J* = 6.7, 3.2 Hz, 3H). ^13^C NMR (101 MHz, CDCl_3_) δ 162.45, 158.92, 154.50, 151.79, 143.50, 136.67, 134.09, 121.58, 119.05, 111.26, 104.25, 98.56, 57.67, 57.15, 55.57, 44.51, 32.40, 32.14, 24.71, 21.69. HRMS (ESI-TOF) *m*/*z* [M − H]^−^ calcd for C_21_H_25_N_7_O_2_ 406.1997, found 406.1989.

### 2.2. In Vitro Assay for Potential PDE9 Inhibitors

#### 2.2.1. Expression and Purification of PDE9 Protein

The expression and purification protocols of PDE9 recombinant catalytic domain (amino acid 181–506) were similar to our previously reported works [32]. In brief, E. coli strain BL21 (Codonplus), which carrying with recombinant pET-PDE9A2 plasmid, was grown in LB medium (containing 100 μg/mL ampicillin and 0.4% glucose) at 37 °C until an OD_600_ achieved 0.6–0.8. Then, the temperature was turned down to 15 °C, and 0.1 mM iso-propyl-b-D-thiogalactopyranoside was added to LB medium to induce the expression of PDE9A2 protein. Bacteria were induced for 20 h and collected for purified PDE9A2 recombinant protein by a Ni-NTA column (Qiagen, Venlo, The Netherlands). The purification batch yielded 60–120 mg of PDE9A2 protein from a 1 L cell culture, with purity exceeding 90%.

#### 2.2.2. Enzymatic Assays Against PDE9

As for the enzymatic assays, ^3^H-cGMP (20,000–30,000 cpm, GE Healthcare), the substrate for enzymatic assays against PDE9A2, were diluted in an assay buffer (containing 50 mM Tris-HCl (pH 8.0), 10 mM MgCl_2_, and 1 mM DTT). And then, the substrate ^3^H-cGMP, catalytic domain of PDE9A2, and different concentrations of tested compounds were incubated at room temperature for 15 min. To terminate the reaction, 0.2 M ZnSO_4_ and 0.2 N Ba(OH)_2_ were added to the mixture, leaving unreacted ^3^H-cGMP in the supernatant. The radioactivity of the supernatant was measured using the PerkinElmer 2910 liquid scintillator counter (PerkinElmer, Waltham, MA, USA) for the calculated inhibition rates. Each compound was measured at a minimum of eight concentrations for the IC_50_ calculation and repeated at least three times. 1-Cyclopentyl-6-(((*R*)-1-((*S*)-3-fluoropyrrolidin-1-yl)-1-oxopropan-2-yl)amino)-1,5-dihydro-4*H*-pyrazolo [3,4-*d*]-pyrimidin-4-one, synthesized by our group, was used as the reference compound with around 50% inhibition against PDE9 at a concentration of 10 nM [32].

### 2.3. Antioxidant Activity Assay

The modified Oxygen Radical Absorbance Capacity Fluorescein (ORAC-FL) method was used for antioxidant activity test [33,34]. In brief, the mixture of test compounds (20 μL) and fluorescein (120 μL, final concentration of 150 nM) was added into a black 96-well plate and incubated for 15 min at 37 °C. Then, 60 μL of 2,2′-azobis(2-amidinopropane) dihydrochloride (AAPH) solution (12 mM) was added rapidly to induce the reaction. The fluorescence with an excitation wavelength of 485 nm and emission at 535 nm was measured in Spectrafluor Plus plate reader (Tecan, Crailsheim, Germany) every minute for 4 h. Integration of the area under the curve (AUC) was Final concentration 1 to 8 μM Trolox was used as a standard. Each compound was tested at 1–10 μM at least in three independent assays. The ORAC-FL values of each compound were calculated according to Equation (1) and expressed as Trolox equivalents.

(1) $$\mathrm{ORAC-FL}\;value=\frac{{AUC}_{\mathrm{sample}}-{AUC}_{\mathrm{blank}}}{{AUC}_{\mathrm{Trolox}}-{AUC}_{\mathrm{blank}}}\times \frac{{Concentration}_{\mathrm{Trolox}}}{{Concentration}_{\mathrm{sample}}}$$

### 2.4. Molecular Docking

The crystal structure of PDE9A complexed with a highly selective inhibitor 1-Cyclopentyl-6-(((*R*)-1-((*S*)-3-fluoropyrrolidin-1-yl)-1-oxopropan-2-yl)amino)-1,5-dihydro-4*H*-pyrazolo [3,4-*d*]-pyrimidin-4-one (PDB ID: 6A3N) was used in this study [32]. Molecular docking was conducted using Surflex-dock, a component of Tripos Sybyl (version X2.0, Tripos Software, Inc., El Cerrito, CA, USA) [35]. Two metal ions present within this crystal structure were retained due to their critical role in the catalytic activities of PDEs. All but a few water molecules were removed from this crystal structure. The exception is for those water molecules located adjacent to the metal ions. Hydrogen atoms were added, and the ionizable residues were protonated to reflect neutral pH conditions. The protomol represents a group of molecular fragments selected from CH_4_, C=O, and N–H, and was placed in PDE9’s pocket by identifying empty 1 Å voxels between marked residues using the bound ligand as the reference. It was then automatically optimized locally by 3D Gaussian smoothing for placement, with high-scoring fragments retained, constituting the docking site. The parameters proto_thresh and proto_bloat indicate the extent to which the protomol can be buried within the protein and the permissible outward extension beyond the cavity, respectively. The proto_thresh was set to 0.5, while the proto_bloat was designated as 0. After preparing the protomol, molecular docking was conducted. The parameter of “Maximum Number of Poses per Ligand” was set to 10, resulting in at most 10 top-ranked docked conformations retained. CScore calculations were performed to obtain docking scores between each conformation and PDE9. All designed molecules were docked to the prepared PDE9 protein, and the top 20 molecules possessing both higher docking scores and suitable binding patterns were selected for subsequent MD simulations, followed by binding free energy calculations.

### 2.5. Molecular Dynamics Simulations

The MM-GBSA approach [36] was utilized for calculating binding-free energies according to 100 snapshots taken from the eighth nanosecond of every MD simulation trajectory, as previously reported [29,30]. The electrostatic contribution to the solvation-free energy was calculated by the PBSA program embedded in the Amber 16 suite, with a grid size of 0.5 Å. The interior and exterior dielectric constants for the solute and solvent were set to 1 and 80, respectively. The optimized atomic radii provided in Amber 16 were applied. The entropy contributions were omitted to enhance computational efficiency without significantly sacrificing accuracy.

### 2.6. Prediction of Pharmacokinetic Properties

Pharmacokinetic properties, such as lipophilicity, solubility, blood-brain barrier (BBB) permeant, and inhibition of cytochrome P450 proteins were estimated using the online web tool SwissADME (http://www.swissadme.ch (accessed on 14 November 2024)).

## 3. Results and Discussion

### 3.1. Multitarget-Directed Ligand Design Strategy

**Figure 2:**
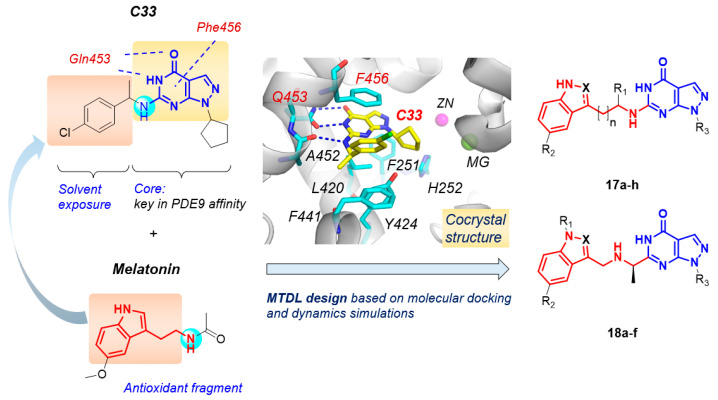
MTDL design strategy combining PDE9 inhibition and antioxidant capacities. The R_1_, R_2_, and R_3_ represent alternative groups. The X represents carbon or nitrogen, and the n represents 1 or 2.

We have previously reported pyrazolopyrimidinone derivatives as effective PDE9 inhibitors exemplified by **C33** (IC_50_ = 16 nM) [31]. The X-ray cocrystal structure of PDE9 with **C33** shows that the pyrazolopyrimidinone core is sandwiched by Phe456 and Leu420, forming strong hydrophobic interaction and π-π stacking. Hydrogen bonds can be observed between the core of **C33** and Gln453. Since the pyrazolopyrimidinone core plays a crucial role in PDE9 recognition, it was retained for further design. The 6-((1-(4-chlorophenyl)ethyl)amino) group of **C33** stretches into a large solvent-exposed region of PDE9, providing space for the introduction of substituents with antioxidant activities. Therefore, compounds with both PDE9 inhibitory activities and antioxidant capacities were designed by attaching different melatonin-derived fragments to the pyrazolopyrimidinone core at the *C*6-position (Figure 2). Molecular docking and dynamics simulations were performed before chemical synthesis and bioassay. The binding free energies were predicted by the MM-GBSA method ranging from −40 to −31 kcal/mol, indicating that all these compounds have strong interactions with PDE9.

### 3.2. Chemistry

**Scheme 1:**
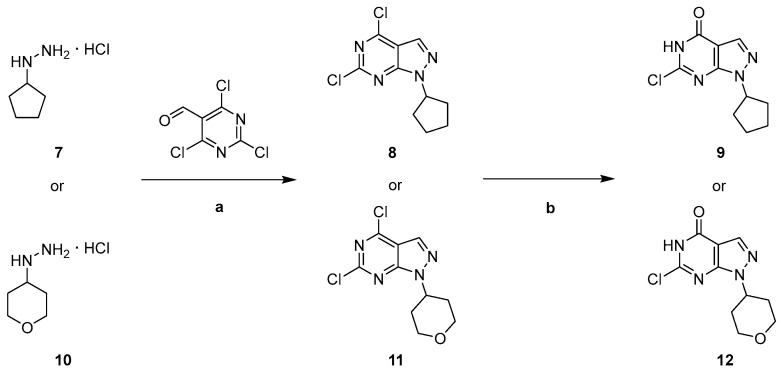
Synthetic route for intermediates **9** and **12**. Reagents and conditions: (**a**) triethylamine, EtOH, −78 °C, 2 h, heated to r. t., 8 h; (**b**) 4% NaOH, 60 °C, 1 h.

The preparation route of intermediates **9** and **12** is displayed in Scheme 1. Intermediates **8** and **11** were synthesized using substituted hydrazine hydrochloride and 2,4,6-trichloropyrimidine-5-carbaldehyde. Subsequently, **8** or **11** experienced hydrolysis reactions using sodium hydroxide as the base to afford intermediates **9** and **12**.

**Scheme 2:**
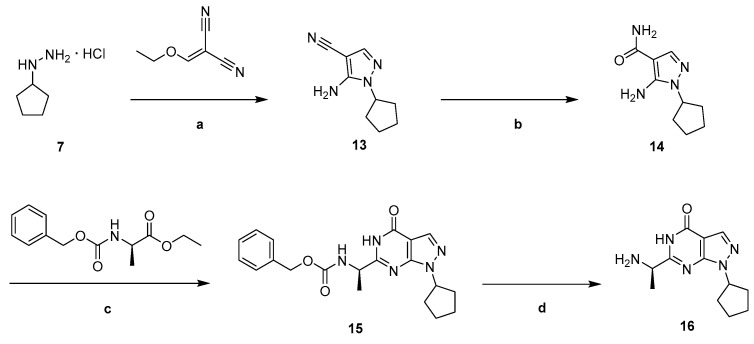
Synthetic route for intermediate **16**. Reagents and conditions: (**a**) triethylamine, EtOH, 0 °C, 2 h, then reflux, 3 h; (**b**) 25% NH_3_·H_2_O, 30% H_2_O_2_, EtOH, r.t., 1 h; (**c**) 60% NaH, anhydrous THF, r.t., 8 h; (**d**) MeOH, Pd/C, H_2_, r.t., 24 h.

The preparation route of intermediate **16** is displayed in Scheme 2. Intermediate **13** were synthesized using cyclopentylhydrazine hydrochloride (**7**) and 2-(ethoxymethylene)malononitrile. Subsequently, the cyano group of **13** underwent a hydrolytic process in the presence of hydrogen peroxide to afford **14** containing an amide group. Intermediate **14** was then reacted with (*R*)-ethyl-2-(((benzyloxy)carbonyl)amino)propanoate under basic condition to afford **15**. The benzyloxyl group was left by hydrogenation in hydrogen atmosphere with Pd/C as the catalyst, affording intermediate **16**.

**Scheme 3:**
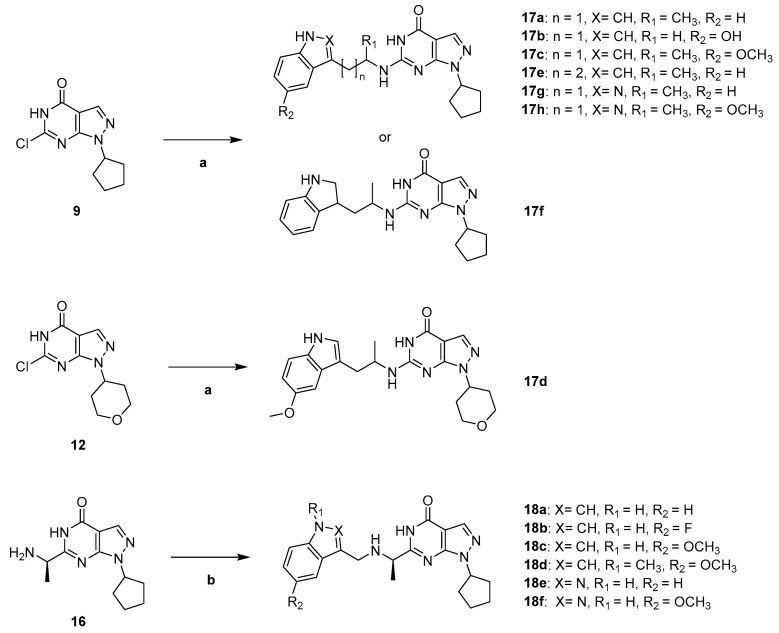
Synthetic route for compounds **17a**–**h** and **18a**–**f**. Reagents and conditions: (**a**) isopropanol, triethylamine, refluxed, 8 h; (**b**) anhydrous AcONa, isopropanol, NaBH_3_CN, r.t., 16 h.

The preparation route of pyrazolopyrimidinone derivatives **17a**–**h**, **18a**–**f** is displayed in Scheme 3. Different amines were reacted with intermediates **9** and **12**, respectively, to afford **17a**–**h** in moderate to high yields. Different aldehydes were reacted with intermediate **16**, resulting in the formation of **18a**–**f** in moderate to high yields.

### 3.3. Biological Evaluation of Designed Compounds

The enzymatic assay of designed compounds against PDE9 was performed in vitro. The antioxidant activity test was also performed by the ORAC method. The inhibitory and antioxidant activities of these compounds are summarized in Table 1 and Table 2.

#### 3.3.1. Inhibition of Designed Compounds Against PDE9

In vitro enzymatic assays for PDE9 inhibition of designed compounds combining the pharmacophore features of PDE9 inhibitors and antioxidant fragments were performed, and most of the targeted compounds showed moderate to high inhibition against PDE9 (Table 1 and Table 2). There were 10 compounds with over 50% inhibition at a concentration of 0.1 μM, while seven compounds showed over 50% inhibition at a concentration of 0.01 μM. Among these compounds, **17c** showed an excellent IC_50_ value of 1.8 nM, indicating that **17c** is a potent PDE9 inhibitor.

Overall, compounds **17a**–**h** showed stronger inhibition against PDE9 compared with compounds **18a**–**f**. A methyl group (R_1_ position) on the side chain linked to the *C*6-position of the pyrazolopyrimidinone core benefits the inhibitory activity, since compound **17b** showed only 16% inhibition (at a centration of 0.01 μM, the same below), much weaker than other compounds such as **17a**, **17c**, and **17e**–**h**. For compounds **17a**–**f** containing an indole ring at the *C*6-position of the core, the substituent groups on indole affected the inhibitory activity. For example, **17c** with 5-methoxyl-1*H*-indol-3-yl (84% inhibition) demonstrated enhanced inhibition than **17a** with 1*H*-indol-3-yl (68% inhibition). On the other hand, changing the *C*6 substituent from (1-(1*H*-indol-3-yl)propan-2-yl)amino to (4-(1*H*-indol-3-yl)butan-2-yl)amino led to compound **17e** (67% inhibition) with comparable inhibitory activity to **17a**, indicating that the chain between indole and the core could be lengthened appropriately. However, when the indole ring in **17a** was replaced with an indoline one, there was a sight loss in potency of **17f** (59% inhibition). A similar decrease in potency can be seen from compound **17c** (84% inhibition) to **17h** (65% inhibition) with an indazole ring, indicating that the indole is a preferable substituent in the *C*6-position of the core. Unexpectedly, compound **17d** displayed a dramatic loss in potency (13% inhibition) compared to **17c**, indicating that tetrahydropyran at the *N*1-position of the pyrazolo[3,4-*d*]pyrimidin-4-one core was unfavorable.

As opposed to the above, introducing a methoxyl at *C*5-position of the indole or indazole ring in compounds **18a** and **18e** (around 50% inhibition, at a concentration of 0.1 μM, the same below) failed to improve the inhibitory activity for compounds **18c** and **18f** (less than 30% inhibition). However, introducing a fluoro group at this position led to compound **18b** (76% inhibition) with a remarkable increase in potency compared to **18a**. In addition, introducing a methyl on the *N*1-position of the indole ring led to **18d** with a slight decrease in potency compared to **18c** (IC_50_ from 194 nM to 214 nM).

#### 3.3.2. Antioxidant Activity of Designed Compounds by the ORAC Method

ORAC-FL is a widely used method for assessing the antioxidant activities of hydrophilic antioxidants. In this study, we evaluated the antioxidant activities of compounds **17a**-**h** and **18a**-**f** using the ORAC-FL method. The results are presented as Trolox equivalents in Table 1 and Table 2. Among the 14 pyrazolopyrimidinone derivatives, compounds **17d**, **18c**, and **18d** with a methoxyl group on the indole ring resembling the fragment of melatonin demonstrated good ORAC values of 2.60-, 1.61-, and 1.09-fold Trolox equivalents. Besides, compound **17b** with a hydroxyl group on the indole ring also demonstrated good ORAC values of 2.00-fold Trolox equivalents. Compounds lacking in a methoxyl or a hydroxyl group on the indole or indoline ring (**17a**, **17e**–**f**, **18a**–**b**) demonstrated low ORAC values. Similarly, compounds with an indazole ring instead of an indole one (**17g**–**h**, **18e**–**f**) exhibited low ORAC values. Unexpectedly, compound **17c** with a fragment of melatonin displayed low antioxidant activity with an ORAC value of 0.32-fold Trolox equivalents.

### 3.4. Pharmacokinetic Properties

Pharmacokinetic properties of compounds **17b** and **17d** were estimated using the online web tool SwissADME. Both **17b** and **17d** have slightly larger molecular weights from 370 to 430. They exhibit favorable lipophilicity, with LogP values ranging from 2 to 4, and show moderate to good solubility. Furthermore, **17d** shows no inhibition against CYP2C9, 2C19, 2D6, and 3A4, indicating a lower likelihood of drug-drug interactions and reduced risk of side effects. However, both **17b** and **17d** are deduced to be P-glycoprotein substrates and possess poor BBB permeant. While the above findings provide valuable insights for MTDLs that target AD through computational and biochemical approaches, the lack of experimental validation in biological systems limits the ability to fully assess the efficacy, safety, and pharmacokinetics of **17b** and **17d**. This limitation should be carefully considered when performing further structural optimization and before proceeding with pharmacokinetic studies, such as cell-based assays and animal model experiments.

### 3.5. Structure-Activity Relationship Analysis Based on Molecular Docking

**Figure 3:**
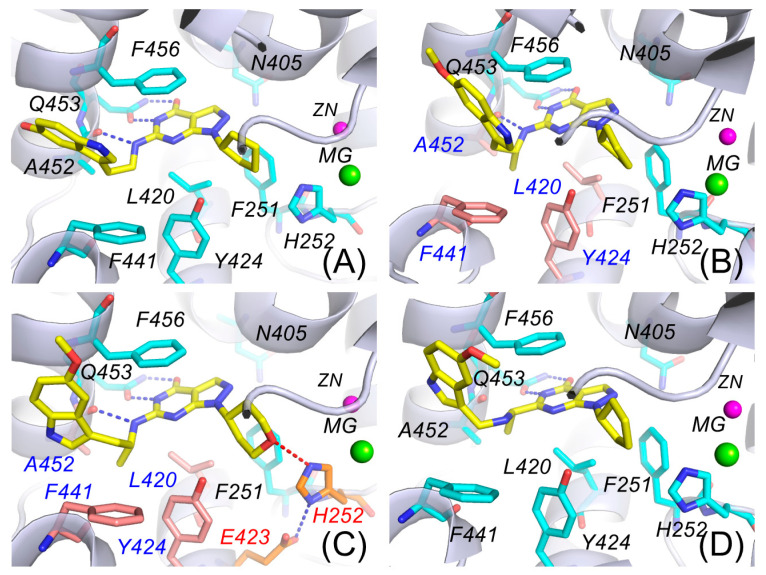
Proposed binding patterns of compounds **17b** (**A**), **17c** (**B**), **17d** (**C**), and **18c** (**D**) with PDE9 (PDB ID: 6A3N). A small hydrophobic pocket is composed of Ala452, Leu420, Tyr424, and Phe441, which are labeled as blue. Hydrogen bonds are depicted in blue dashed lines. Unfavorable electric repulsion is depicted in red dashed lines.

Molecular docking, together with dynamics simulations, were performed in the initial design to reduce synthetic work, and the structure-activity relationship was analyzed aided by the calculation results to explain the differences in inhibitory activity among the targeted compounds. From the binding patterns of typical compounds including **17b**–**d** and **18c** with PDE9 (Figure 3), two crucial hydrogen bonds were formed between each compound and Gln453. Besides, π-π stacking was also observed between the pyrazolo [3,4-*d*]pyrimidin-4-one core and Phe456. These interactions have been validated as important for the recognition of inhibitors and PDE9. Furthermore, an additional hydrogen bond was observed between Ala452 and the amino group of the side chain in compounds **17b**–**d**, which was not observed between compound **18c** and PDE9. Thus, this hydrogen bond with Ala452 was recognized as an important contributive factor to the potency of these inhibitors. The methyl group in the side chain of **17c** occupied a small pocket composed of Leu420, Tyr424, Phe441, and Ala452, leading to the indole ring forming “T-shaped” π-π stacking with Phe456. However, due to a lack of this methyl group in **17b**, the indole ring at the end of the *C*6-substituent stretched directly into the solvent region of PDE9 and could not form π-π stacking with Phe456. This could explain the potency loss from **17c** to **17b**.

The decreased inhibition of compound **17d** compared with **17c** may be due to electric repulsion between the oxygen of the tetrahydropyran ring of **17d** and the nitrogen of the imidazole ring of His252. Although compound **17c** could also form “T-shaped” π-π stacking with Phe456, steric hindrance rather than hydrogen bonding was formed with Ala452, leading to its decreased potency to **17c**.

## 4. Conclusions

In summary, a series of pyrazolo [3,4-*d*]pyrimidin-4-one derivatives were designed and synthesized as multitarget-directed anti-AD ligands. With the aid of molecular docking and dynamics simulations, compounds that combine the pharmacophore features of PDE9 inhibitors with the antioxidant melatonin were evaluated to be novel PDE9 inhibitors with moderate to high potency and remarkable antioxidant activities. Among them, compounds **17b** and **17d** demonstrated IC_50_ values of 91 and 89 nM against PDE9, respectively. They also showed good antioxidant activity with ORAC values of 2.00- and 2.60-fold Trolox equivalents, respectively. Molecular docking-based binding patterns between inhibitors and PDE9 disclosed several important interactions with Gln453, Phe456, and Ala452, which enriched the structure-activity relationship. We believe this MTDL designing strategy is useful for further structural modification of potent PDE9 inhibitors with enhanced antioxidant activities, which could be utilized for further research in AD in the near future.

## 5. Patents

The work reported in this manuscript has been patented (Patent Number: CN106977518A).

## Figures and Tables

**Table 1:** Predicted binding free energies, inhibitory activities against PDE9, and oxygen radical absorbance capacity (ORAC) of compounds **17a**–**h**.

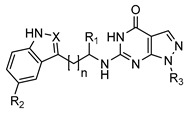 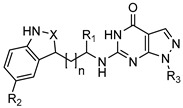 17a–e, 17g–h 17f								
Comp.	n	X	R_1_	R_2_	R_3_	Binding Free Energy (kcal/mol) ^1^	PDE9 IC_50_ (nM)	ORAC ^4^
17a	1	CH	-CH_3_	-H	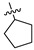	−31.42 ± 2.48	78% ^2^ 68% ^3^	0.37 ± 0.04
17b	1	CH	-H	-OH	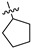	−31.87 ± 3.14	91 ± 4	2.00 ± 0.27
17c	1	CH	-CH_3_	-OCH_3_	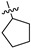	−39.59 ± 2.50	1.8	0.32 ± 0.06
17d	1	CH	-CH_3_	-OCH_3_	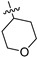	−37.89 ± 2.59	89 ± 4	2.60 ± 0.05
17e	2	CH	-CH_3_	-H	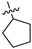	−35.24 ± 2.50	83% ^2^ 67% ^3^	0.33 ± 0.002
17f	1	CH_2_	-CH_3_	-H	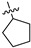	−32.67 ± 3.21	67% ^2^ 59% ^3^	0.66 ± 0.04
17g	1	N	-CH_3_	-H	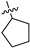	−37.37 ± 2.68	82% ^2^ 70% ^3^	0.17 ± 0.004
17h	1	N	-CH_3_	-OCH_3_	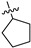	−38.67 ± 2.73	69% ^2^ 65% ^3^	0.22 ± 0.12

^1^ Binding free energies were estimated by the MM-GBSA method. ^2^ Inhibition ratio at a concentration of 0.1 μM. ^3^ Inhibition ratio at a concentration of 0.01 μM. ^4^ Data are expressed as μmol of Trolox equiv/μmol tested compound. Trolox serves as a standard. The ORAC value of melatonin was tested to be 1.85 ± 0.04 Trolox. The means and SD values are calculated from at least three independent experiments.

**Table 2:** Predicted binding free energies, inhibitory activities against PDE9, and oxygen radical absorbance capacity (ORAC) of compounds **18a–f**.

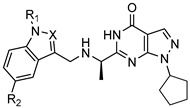						
Comp.	X	R_1_	R_2_	Binding Free Energy (kcal/mol) ^1^	PDE9 IC_50_ (nM)	ORAC ^4^
18a	CH	-H	-H	−32.78 ± 2.27	56% ^2^ 46% ^3^	0.60 ± 0.06
18b	CH	-H	-F	−36.12 ± 2.74	76% ^2^ 61% ^3^	0.88 ± 0.08
18c	CH	-H	-OCH_3_	−37.38 ± 3.19	194 ± 26	1.61 ± 0.11
18d	CH	-CH_3_	-OCH_3_	−36.09 ± 2.85	214 ± 20	1.09 ± 0.02
18e	N	-H	-H	−34.68 ± 2.48	47% ^2^	0.15 ± 0.03
18f	N	-H	-OCH_3_	−32.70 ± 2.81	28% ^2^	0.24 ± 0.02

^1^ Binding free energies were estimated by the MM-GBSA method. ^2^ Inhibition ratio at a concentration of 0.1 μM. ^3^ Inhibition ratio at a concentration of 0.01 μM. ^4^ Data are expressed as μmol of Trolox equiv/μmol tested compound. Trolox serves as a standard. The ORAC value of melatonin was tested to be 1.85 ± 0.04 Trolox. The means and SD values are calculated from at least three independent experiments.

## Data Availability

The data presented in this study are available within the article and Supplementary Materials.

## Supplementary Materials

The following supporting information can be downloaded at: https://www.mdpi.com/article/10.3390/antiox14020123/s1. The spectra of ^1^H-NMR, ^13^C-NMR, and HRMS for the target compounds are included in the uploaded file of supporting information [36,37,38,39,40,41].
