# Supplementary material for: Discovery of Effective Inhibitors Against Phosphodiesterase 9, a Potential Therapeutic Target of Alzheimer’s Disease with Antioxidant Capacities
Source: Antioxidants (Basel). 2025 Jan 21;14(2):123. doi: 10.3390/antiox14020123 (PMC11852235; doi:10.3390/antiox14020123)
Supplement: Supplementary file 1 [file antioxidants-14-00123-s001.zip › antioxidants-3344680-supplementary.pdf]

Discovery of Effective Inhibitors against Phosphodiesterase 9, a potential therapeutic target of Alzheimer's Disease with Antioxidant Capacities

Qian Zhou[1, #], Xu-Nian Wu[2, #], Wei-Hao Luo[1], Qing-Hua Huang[2], Ling-Ling Feng[2], Yinuo Wu[2, *], Chen Zhang[1, *]

[1] School of Chemistry and Chemical Engineering, Guangdong Pharmaceutical University, Zhongshan 528458, China

[2] State Key Laboratory of Anti-Infective Drug Discovery and Development, School of Pharmaceutical Sciences, Sun Yat-sen University, Guangzhou 510006, China

[#] These authors contributed equally to this work.

Table of Contents:

1. ^{1}HNMR and ^{13}CNMR spectrums of the targeted compounds…….………………………..………S2

2. The HRMS spectrums of the targeted compounds……..……………………..…………………S16

3. Molecular dynamics simulations………………………………………………………..S30

4. Enzymatic assays against PDE9.……..……………………..……………………..………………..S34

5. HPLC spectrums for the purity of representative target compounds…………………………….S39

6. References…………………………………………………………………………………..S43

1. ^{1}HNMR and ^{13}CNMR spectrums of the targeted compounds

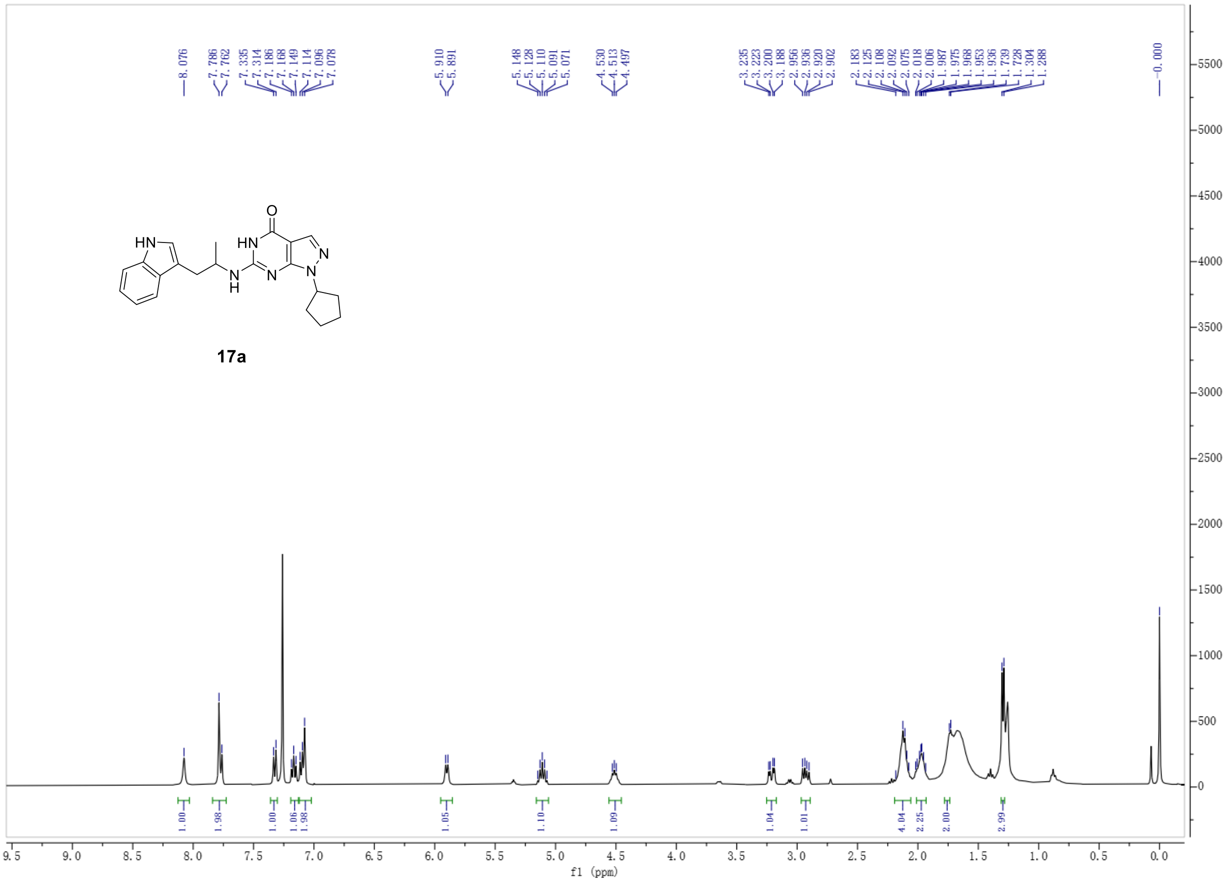

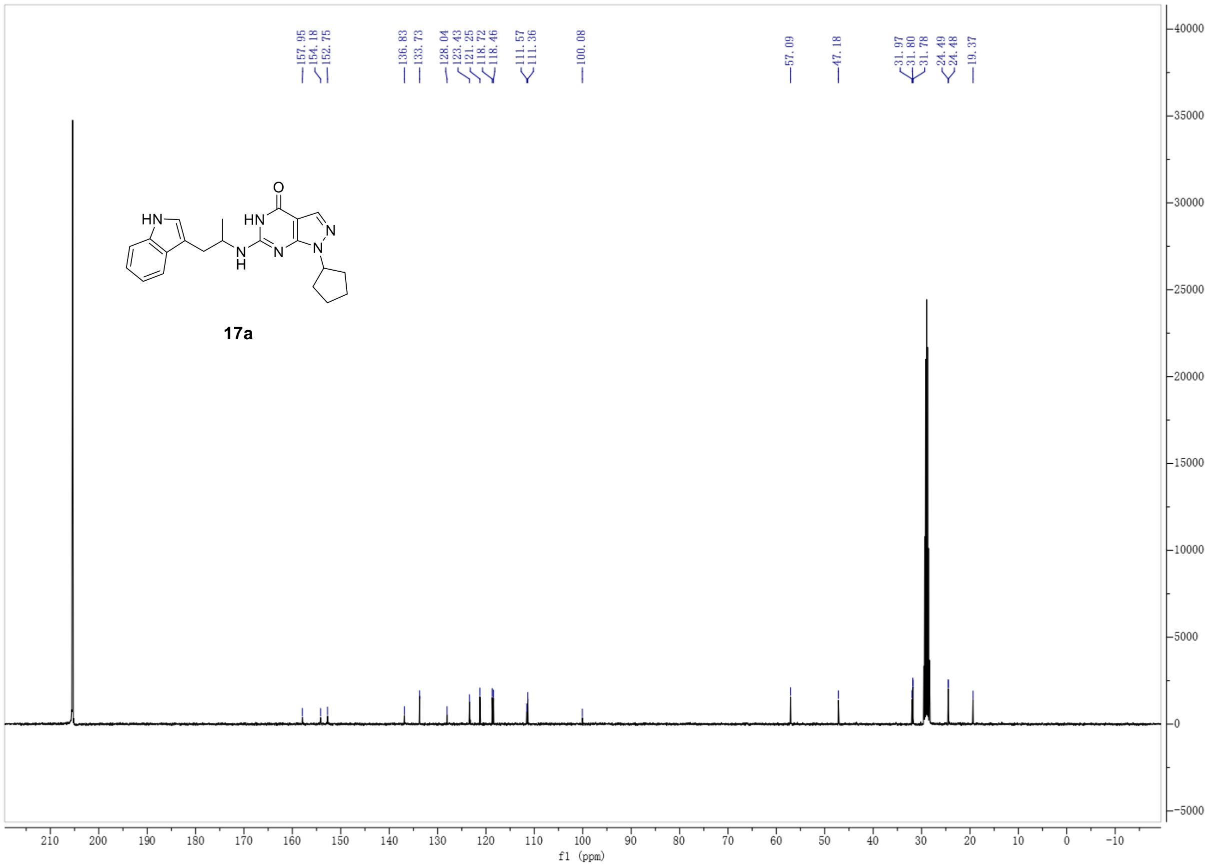

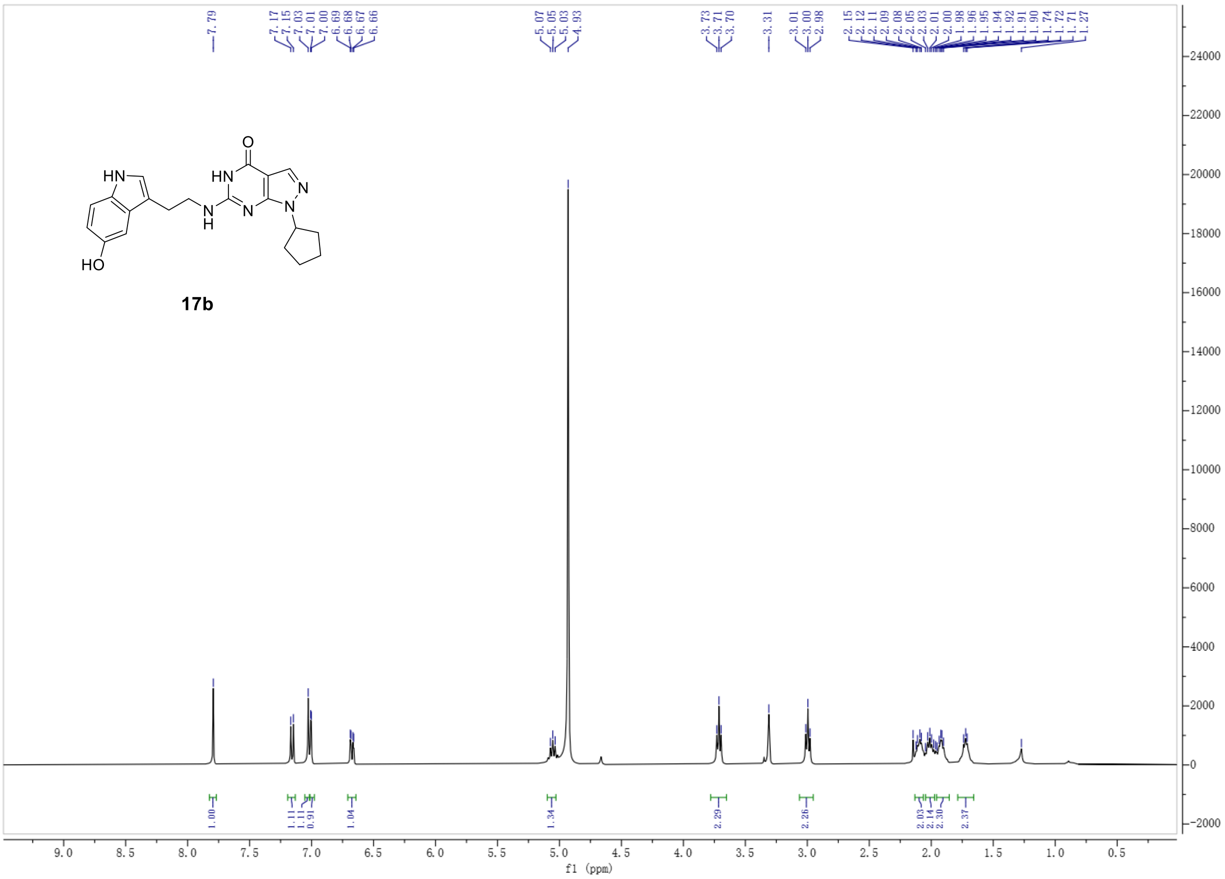
7.79
7.17 7.15 7.03 7.01 7.00 6.69 6.68 6.67 6.66
5.07 5.05 5.03 4.93
3.73 3.71 3.70
3.31
3.01 3.00 2.98
2.15 2.12 2.11 2.09 2.08 2.05 2.03 2.01 2.00 1.98 1.96 1.95 1.94 1.92 1.91 1.90 1.74 1.72 1.71 1.27
1.00 1.11 1.11 0.91 1.04 1.34 2.29 2.26 2.03 2.14 2.30 2.37
HN
HO
O
HN
N
N
N
H
17b
f1 (ppm)

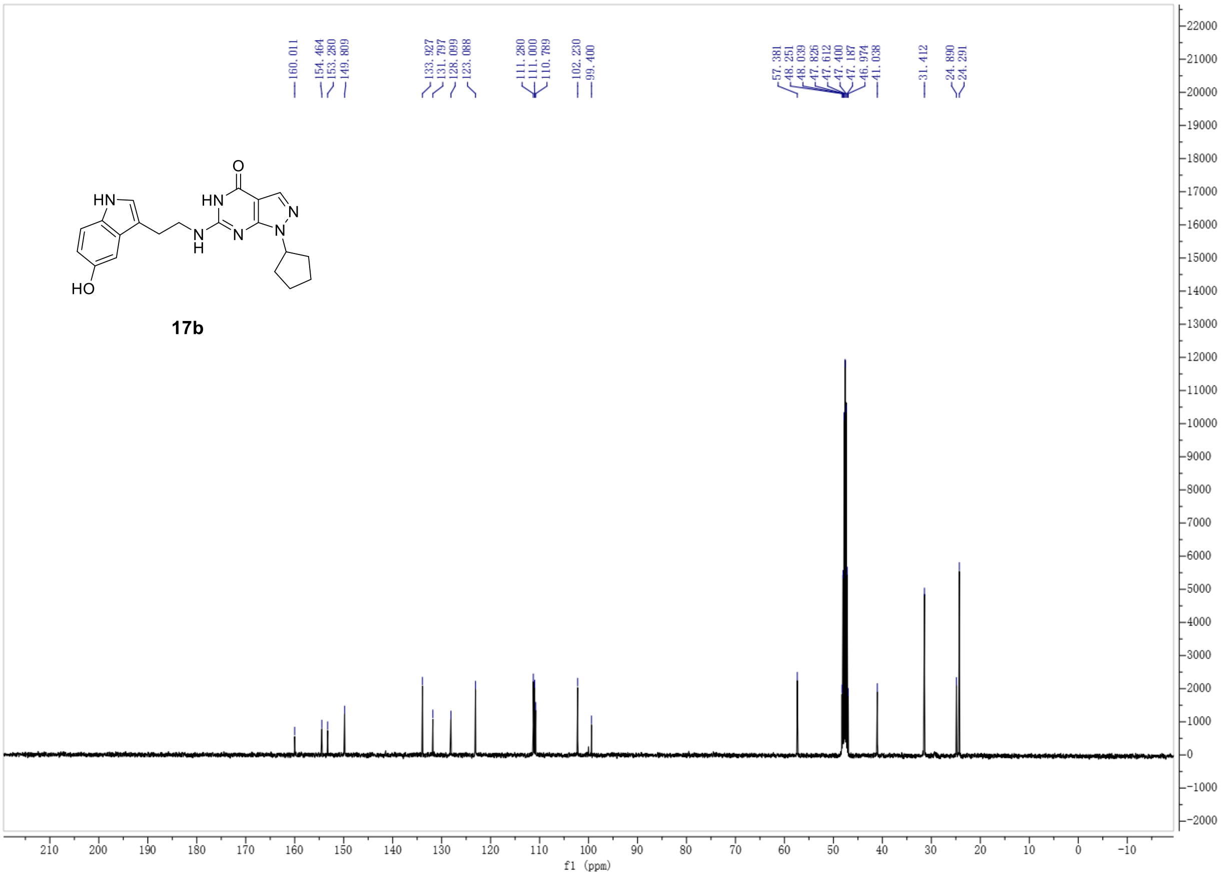
160.011 154.464 153.280 149.809
133.927 131.797 128.099 123.088
111.280 111.000 110.789
102.230 99.400
57.381 48.251 48.039 47.826 47.612 47.400 47.187 46.974 41.038
31.412
24.890 24.291
HN
HO
O
HN
N
N
N
H
17b
f1 (ppm)

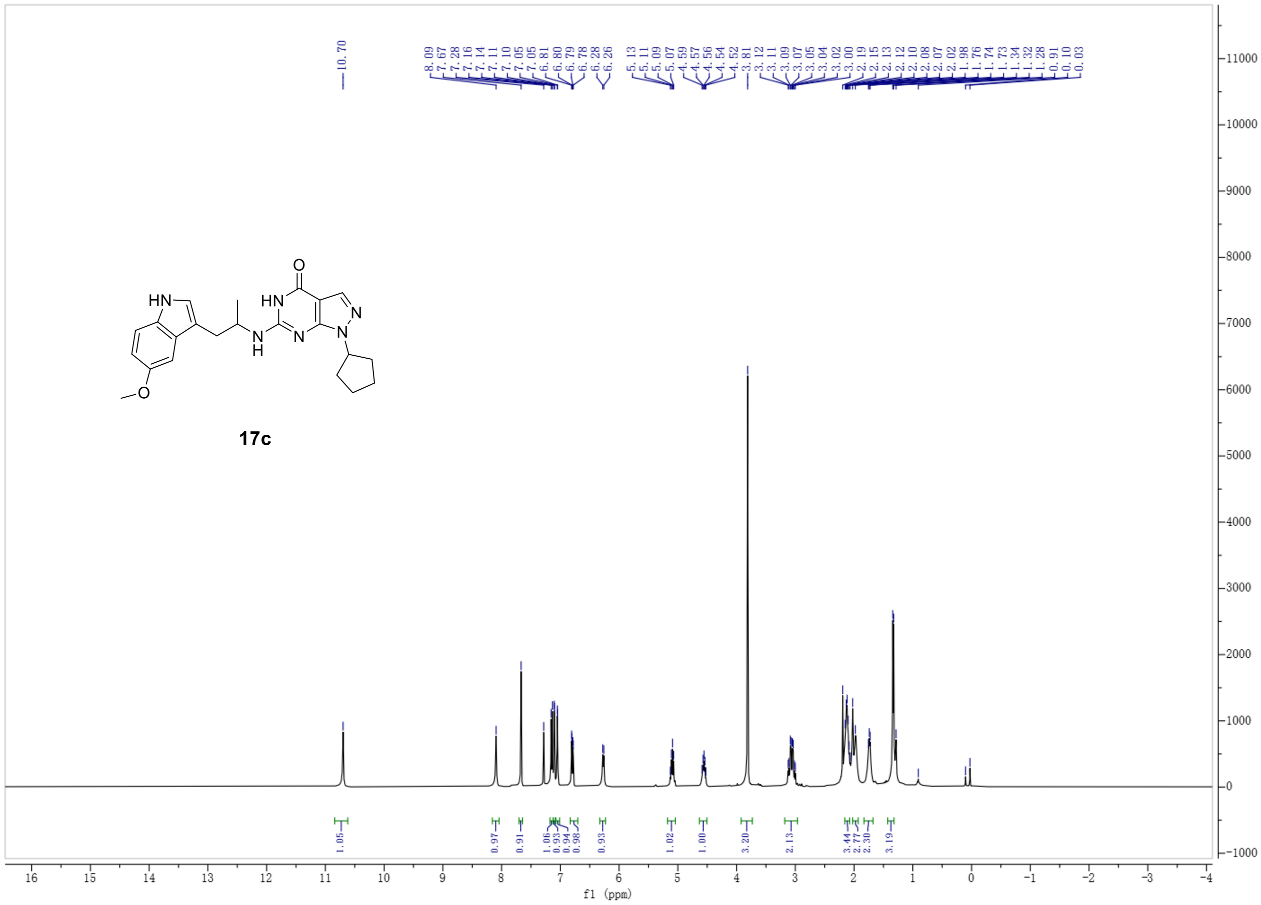
10.70
8.09 7.67 7.28 7.16 7.14 7.11 7.10 7.05 7.05 6.81 6.80 6.79 6.78 6.28 6.26
5.13 5.11 5.09 5.07 4.59 4.57 4.56 4.54 4.52 3.81 3.12 3.11 3.09 3.07 3.05 3.04 3.02 3.00 2.19 2.15 2.13 2.12 2.10 2.08 2.07 2.02 1.98 1.76 1.74 1.34 1.32 1.28 0.91 0.10 0.03
1.05 0.97 0.91 1.06 0.93 0.94 0.98 0.93 1.02 1.00 3.20 2.13 3.44 2.77 2.30 3.19
17c
f1 (ppm)

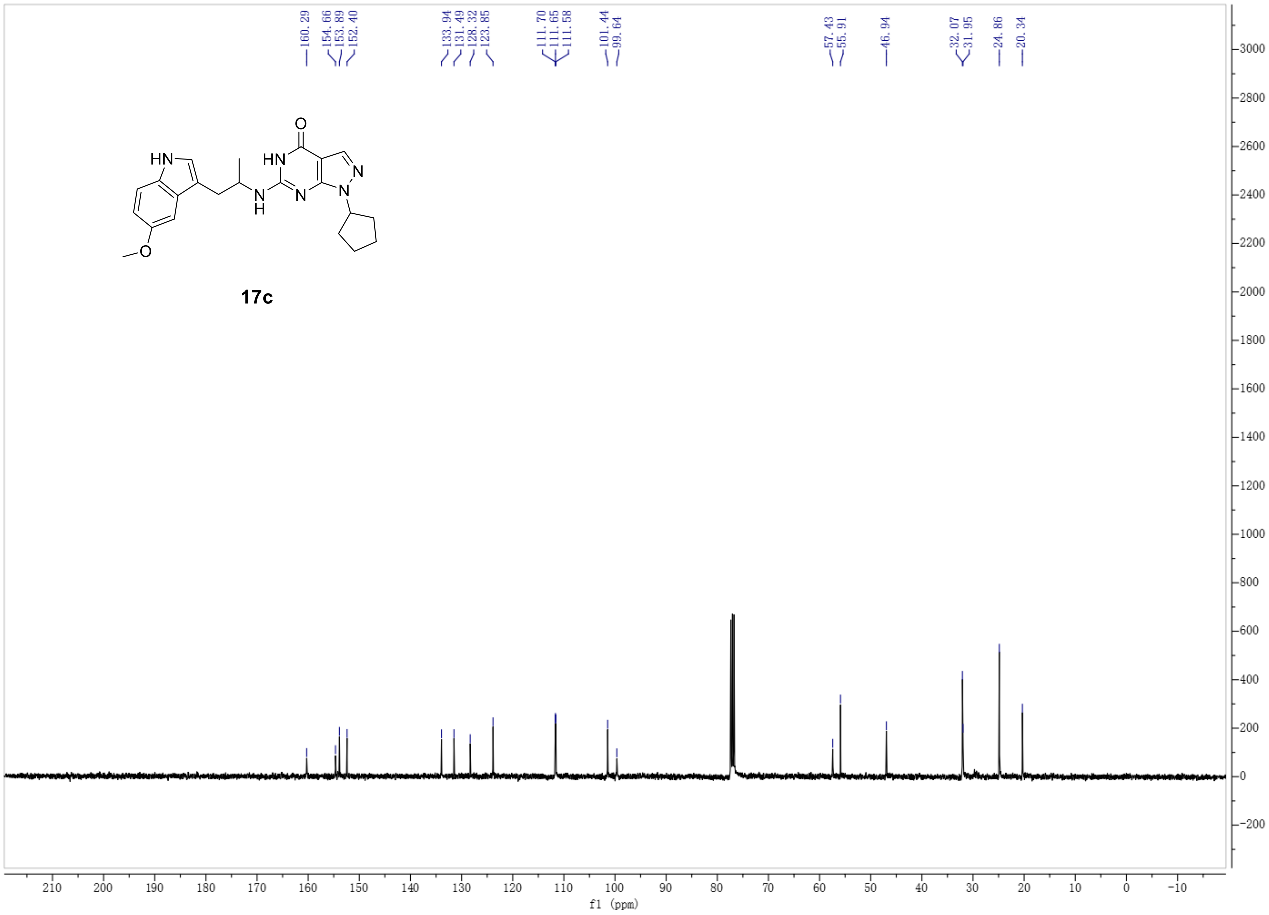
160.29 154.66 153.89 152.40 133.94 131.49 128.32 123.85 111.70 111.65 111.58 101.44 99.64 57.43 55.91 46.94 32.07 31.95 24.86 20.34
17c
f1 (ppm)

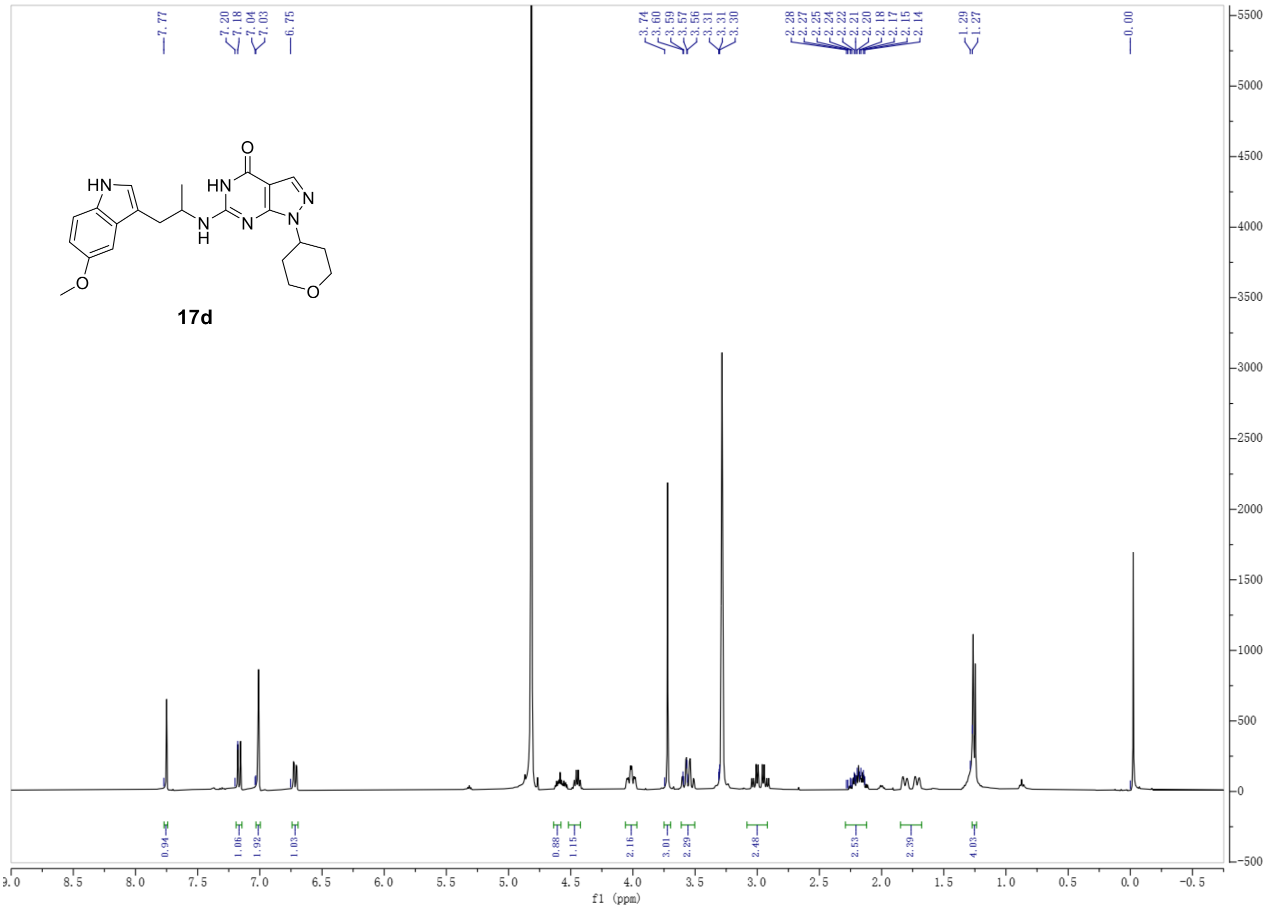
17d
f1 (ppm)

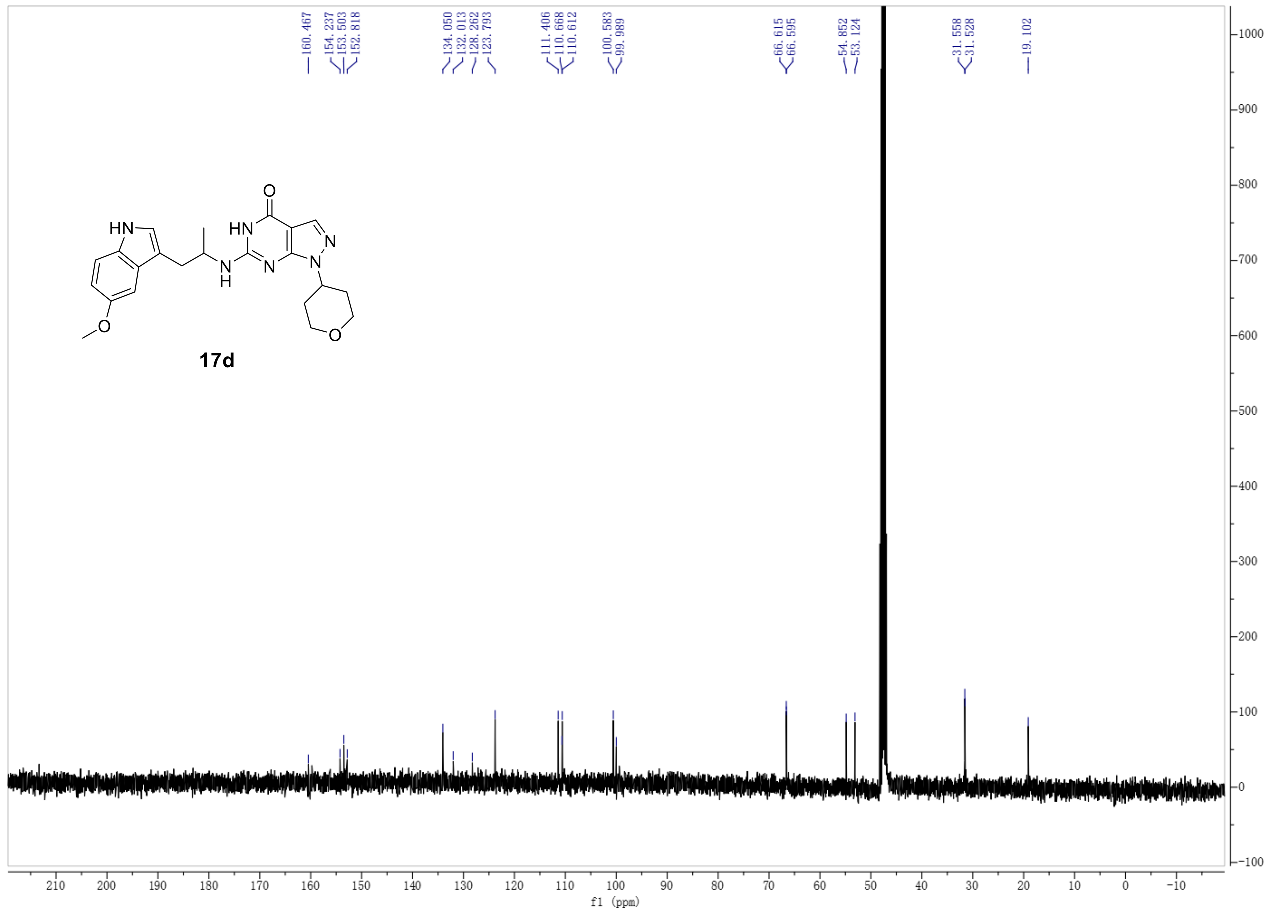
17d
f1 (ppm)

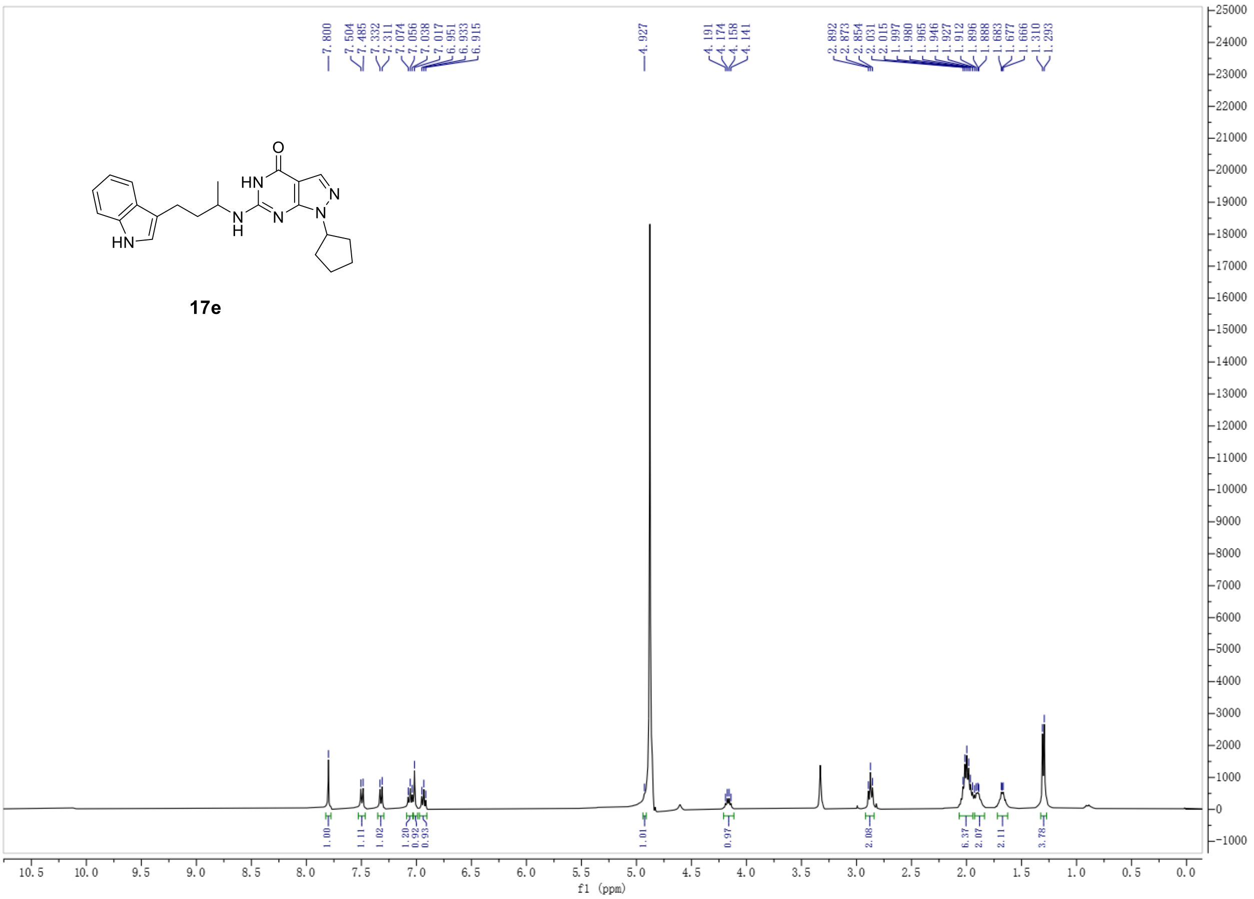
7.800
7.504
7.485
7.332
7.311
7.074
7.056
7.038
7.017
6.951
6.933
6.915
4.927
4.191
4.174
4.158
4.141
2.892
2.873
2.854
2.031
2.015
1.997
1.980
1.965
1.946
1.927
1.912
1.896
1.888
1.683
1.677
1.666
1.310
1.293
f1 (ppm)

17e

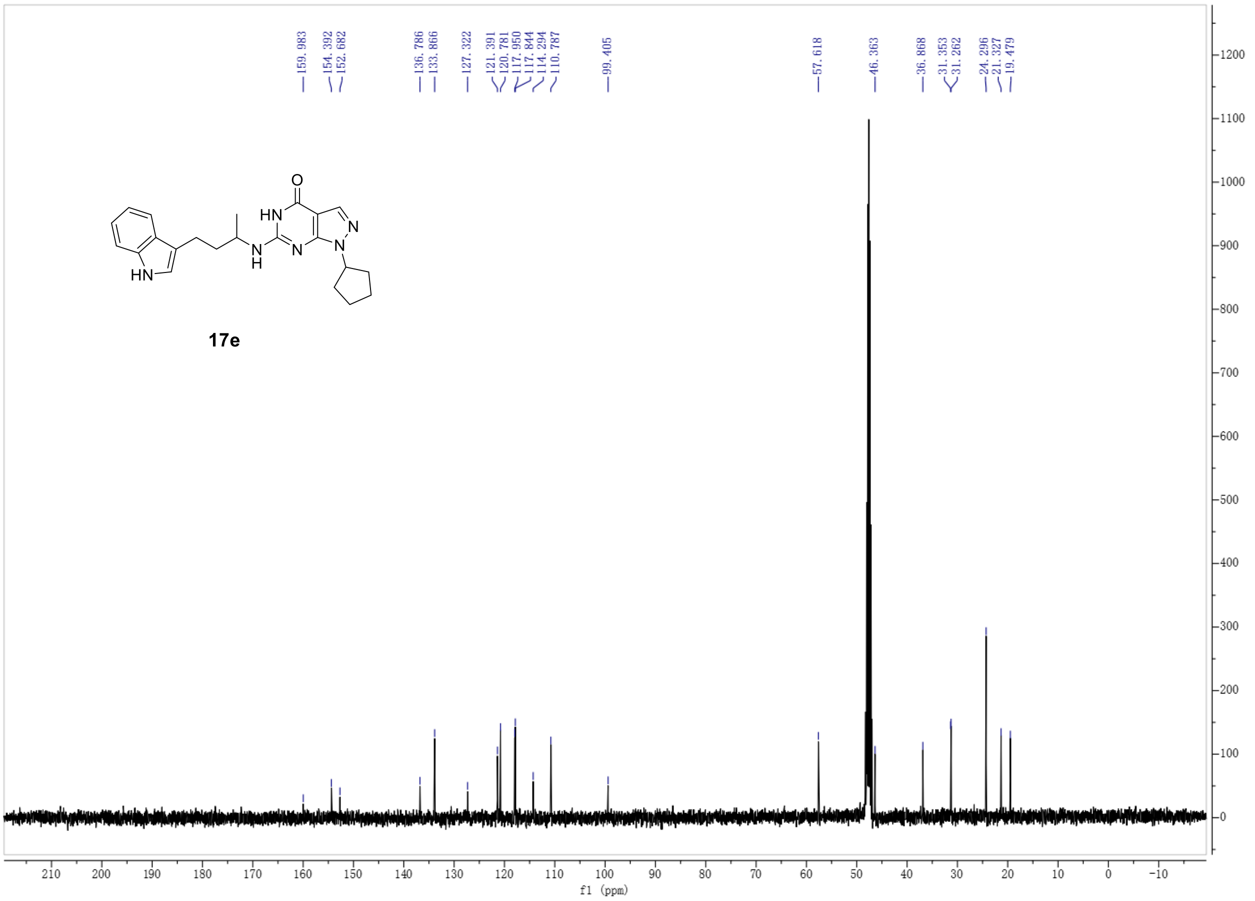
159.983
154.392
152.682
136.786
133.866
127.322
121.391
120.781
117.950
117.844
114.294
110.787
99.405
57.618
46.363
36.868
31.353
31.262
24.296
21.327
19.479
f1 (ppm)

17e

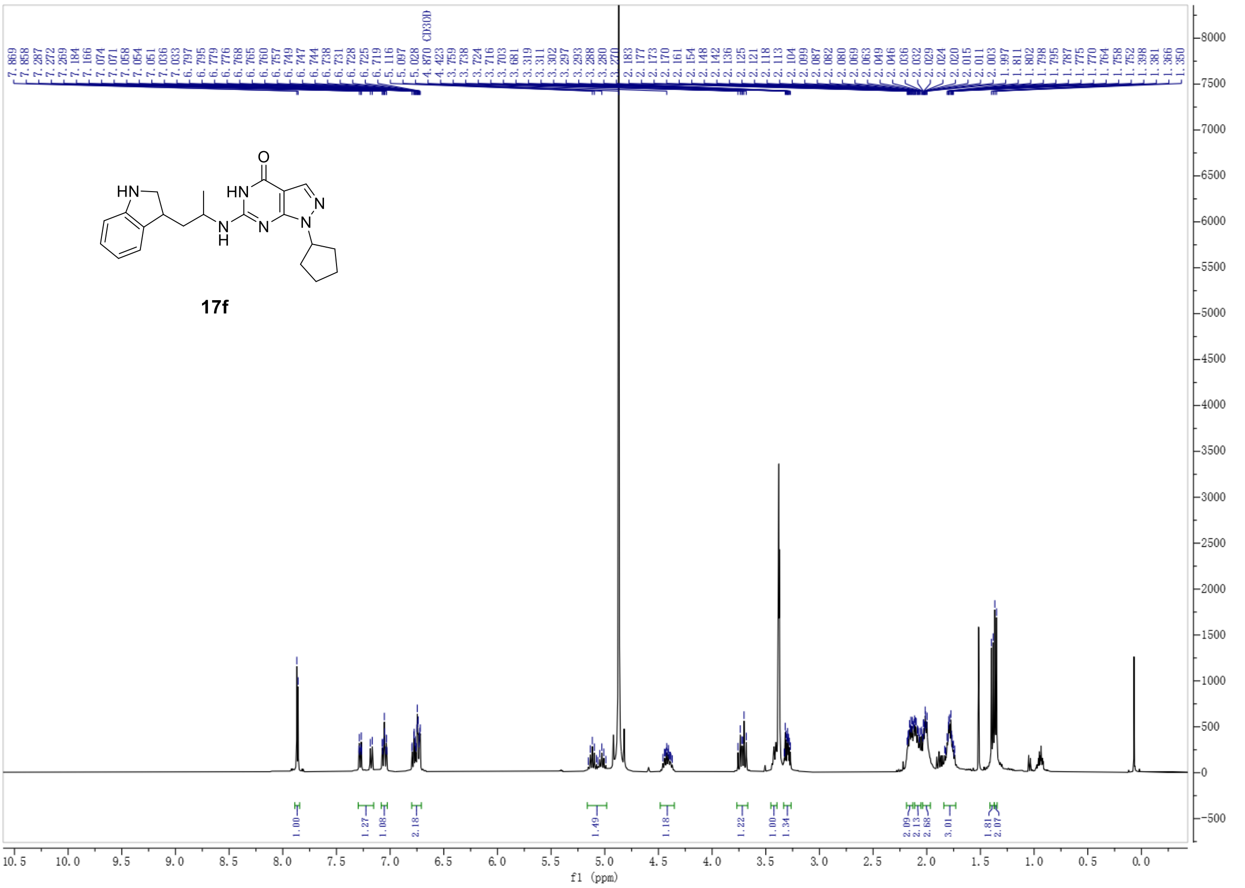
17f
f1 (ppm)

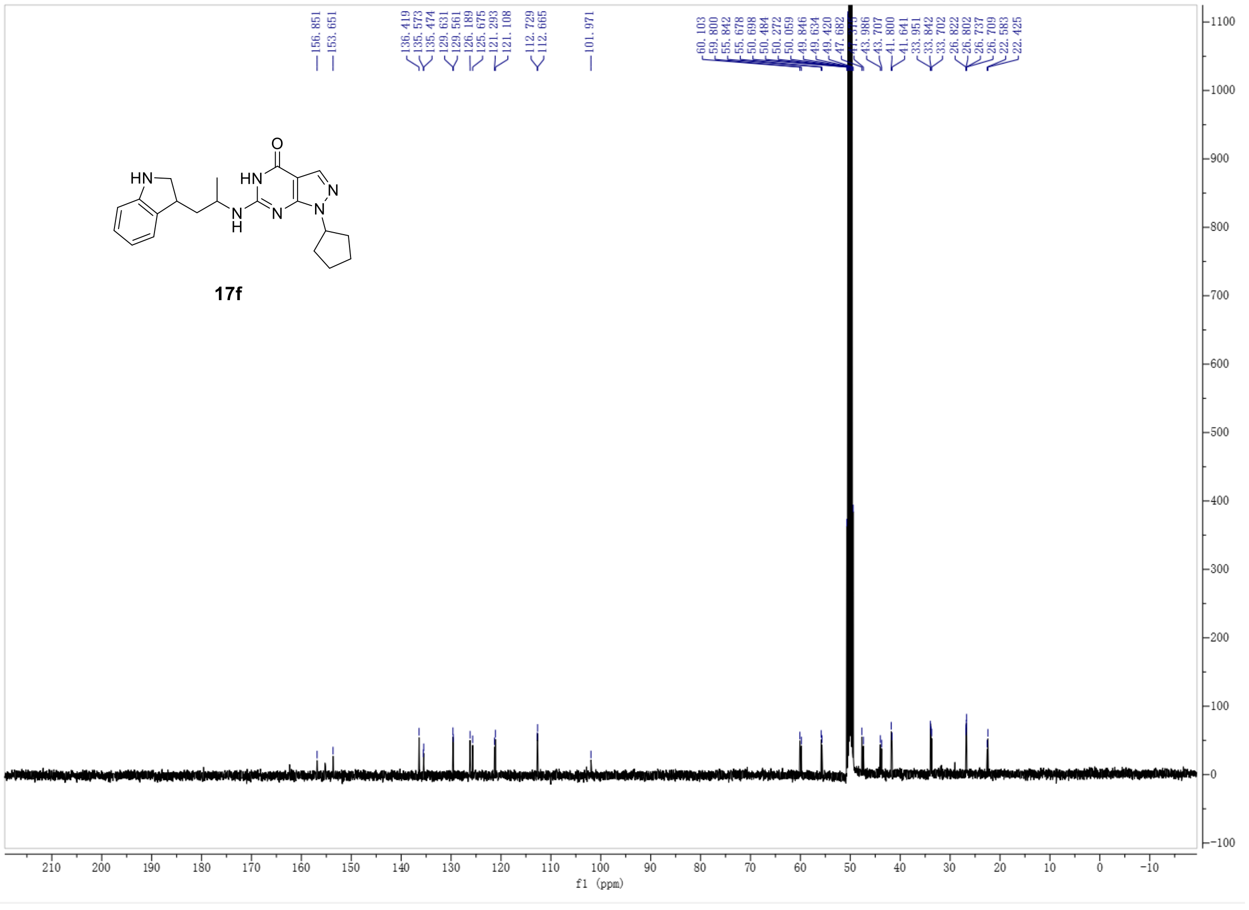
17f
f1 (ppm)

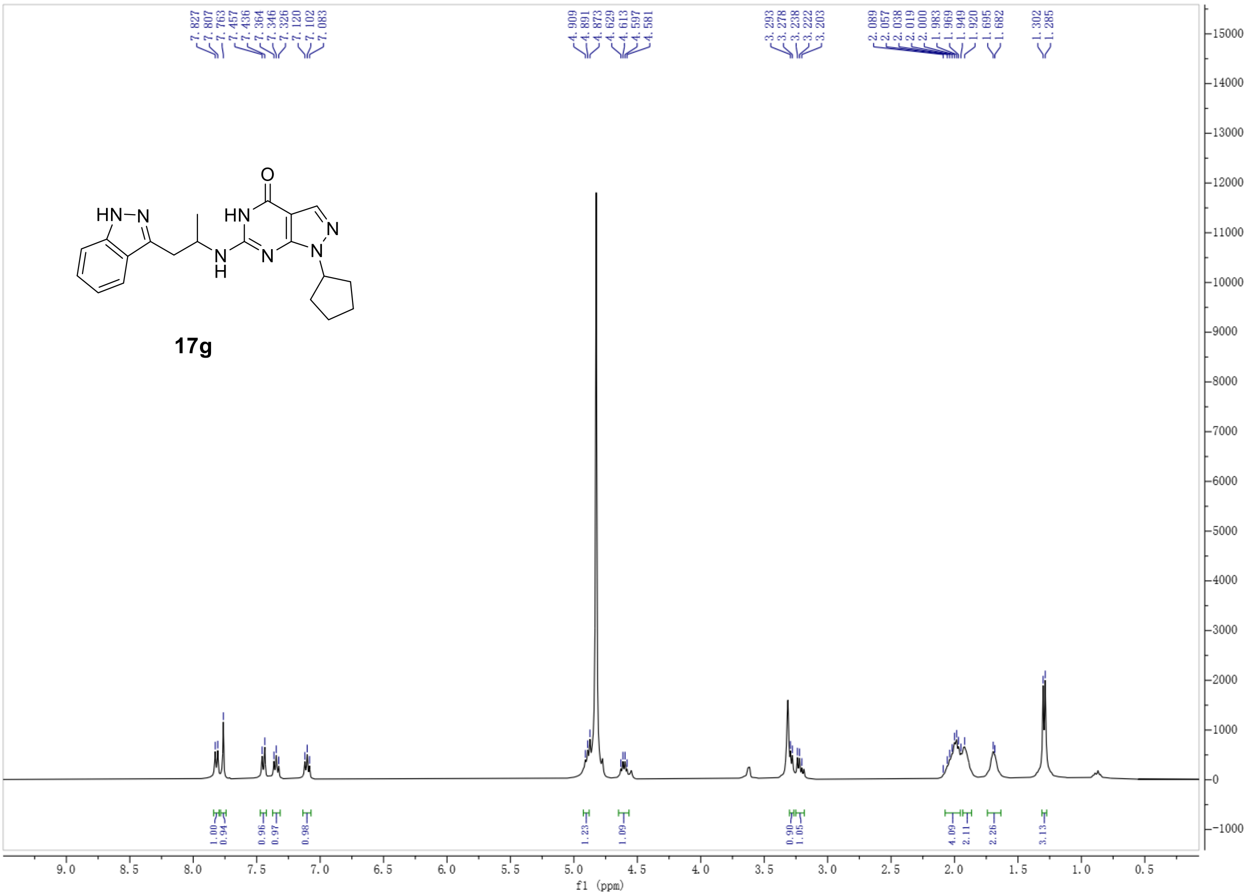
7.827 7.807 7.763 7.457 7.436 7.364 7.346 7.326 7.120 7.102 7.083
4.909 4.891 4.873 4.629 4.613 4.597 4.581
3.293 3.278 3.238 3.222 3.203
2.089 2.057 2.038 2.019 2.000 1.983 1.969 1.949 1.920 1.695 1.682
1.302 1.285
17g
1.00 0.94 0.96 0.97 0.98 1.23 1.09 0.90 1.05 4.09 2.11 2.26 3.13
f1 (ppm)

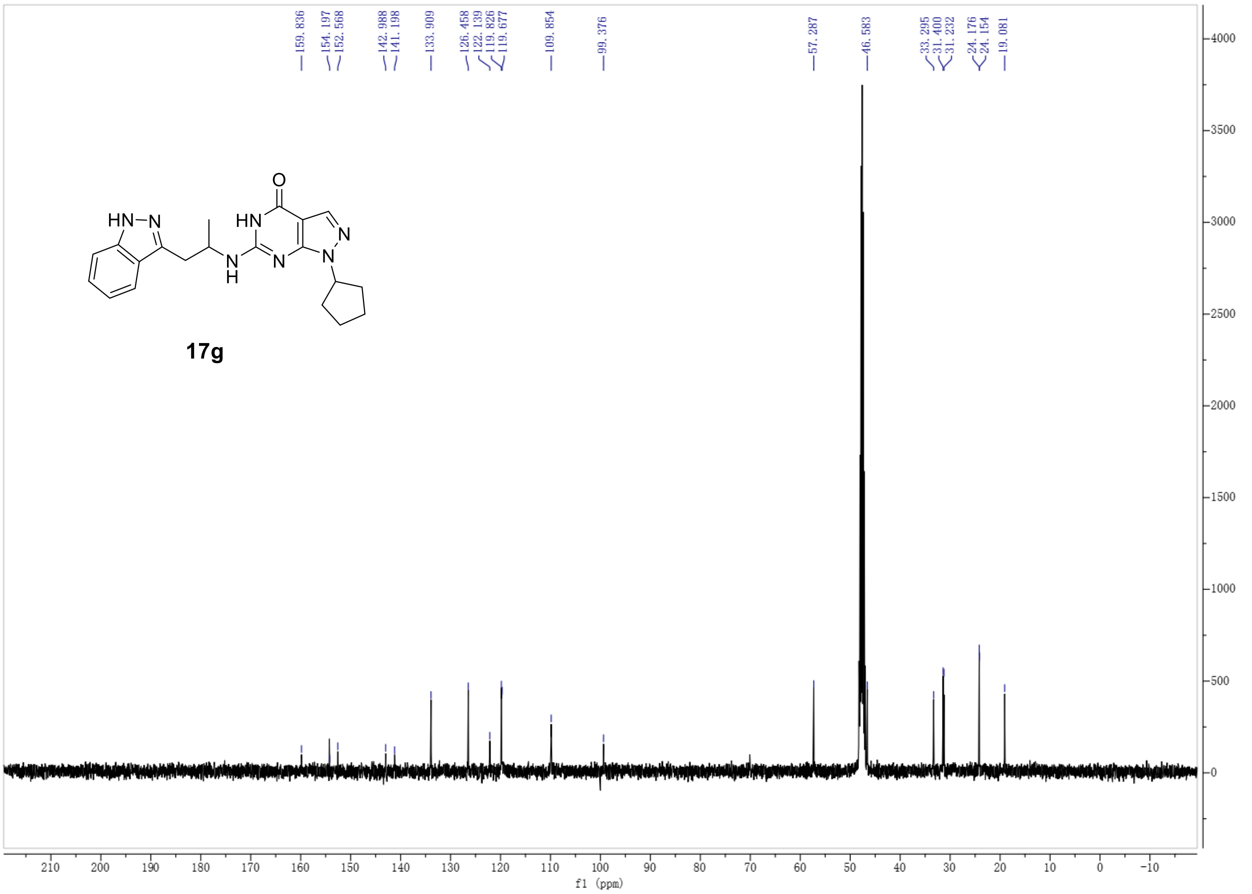
159.836 154.197 152.568 142.988 141.198 133.909 126.458 122.139 119.826 119.677 109.854 99.376
57.287 46.583 33.295 31.400 31.232 24.176 24.154 19.081
17g
f1 (ppm)

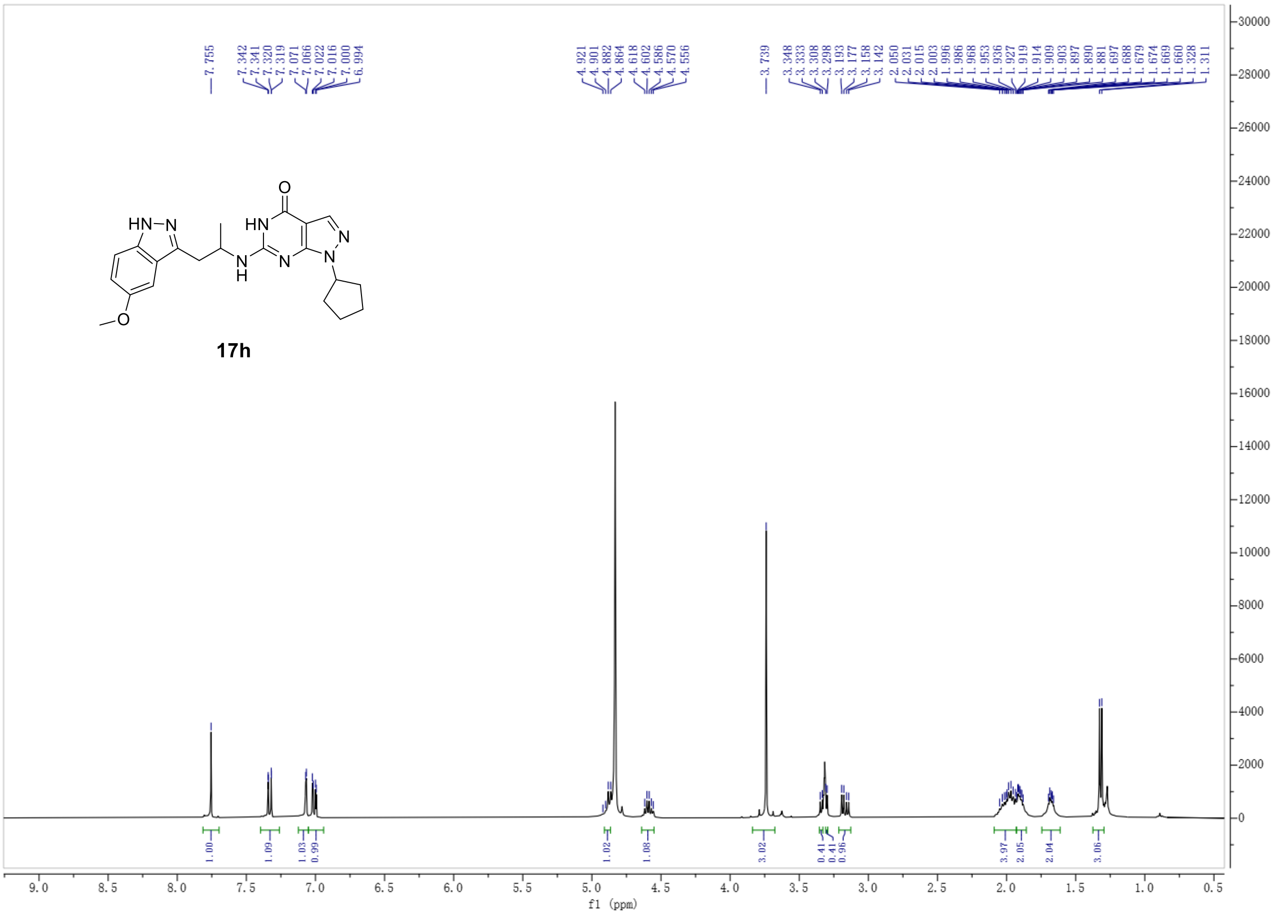
17h
f1 (ppm)

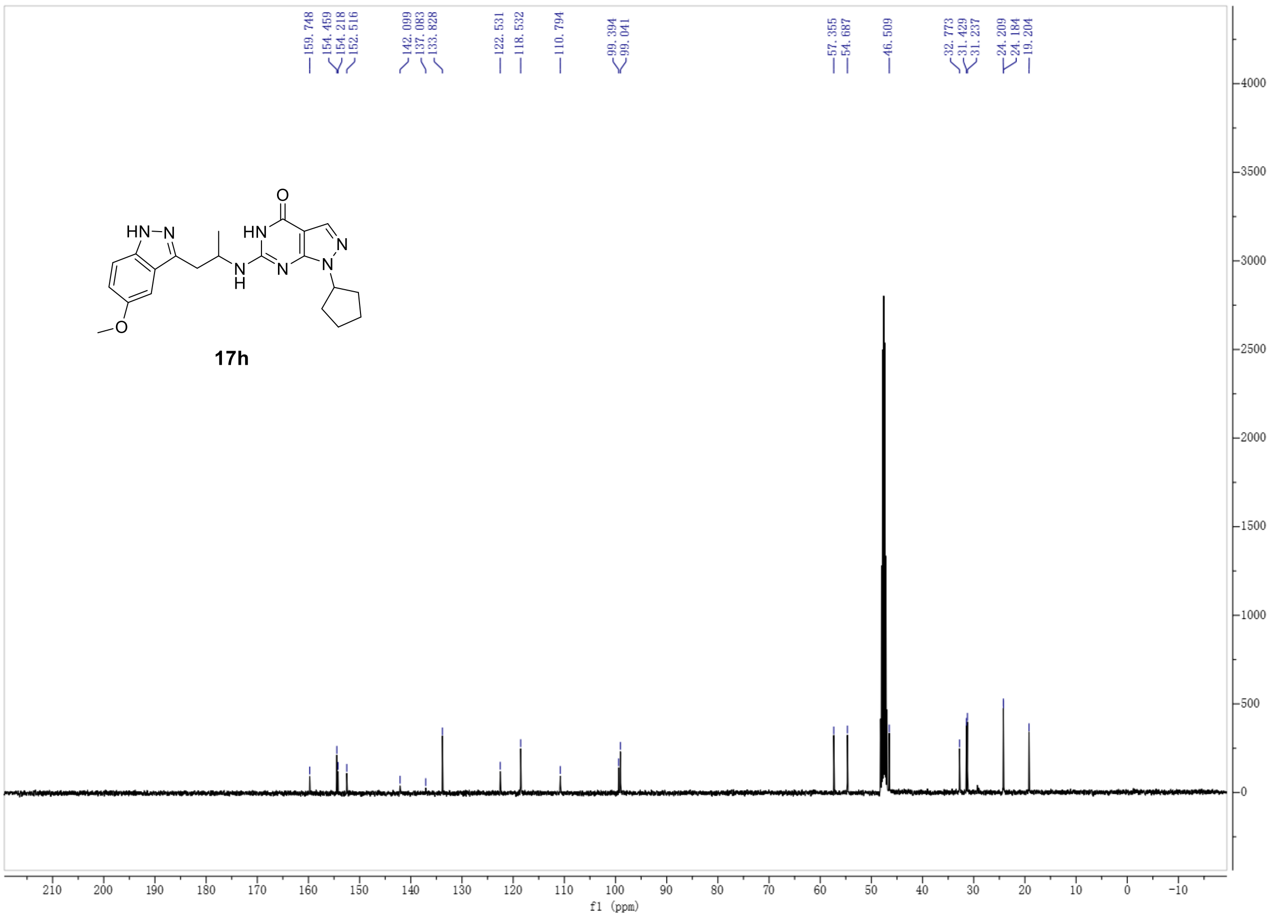
17h
f1 (ppm)

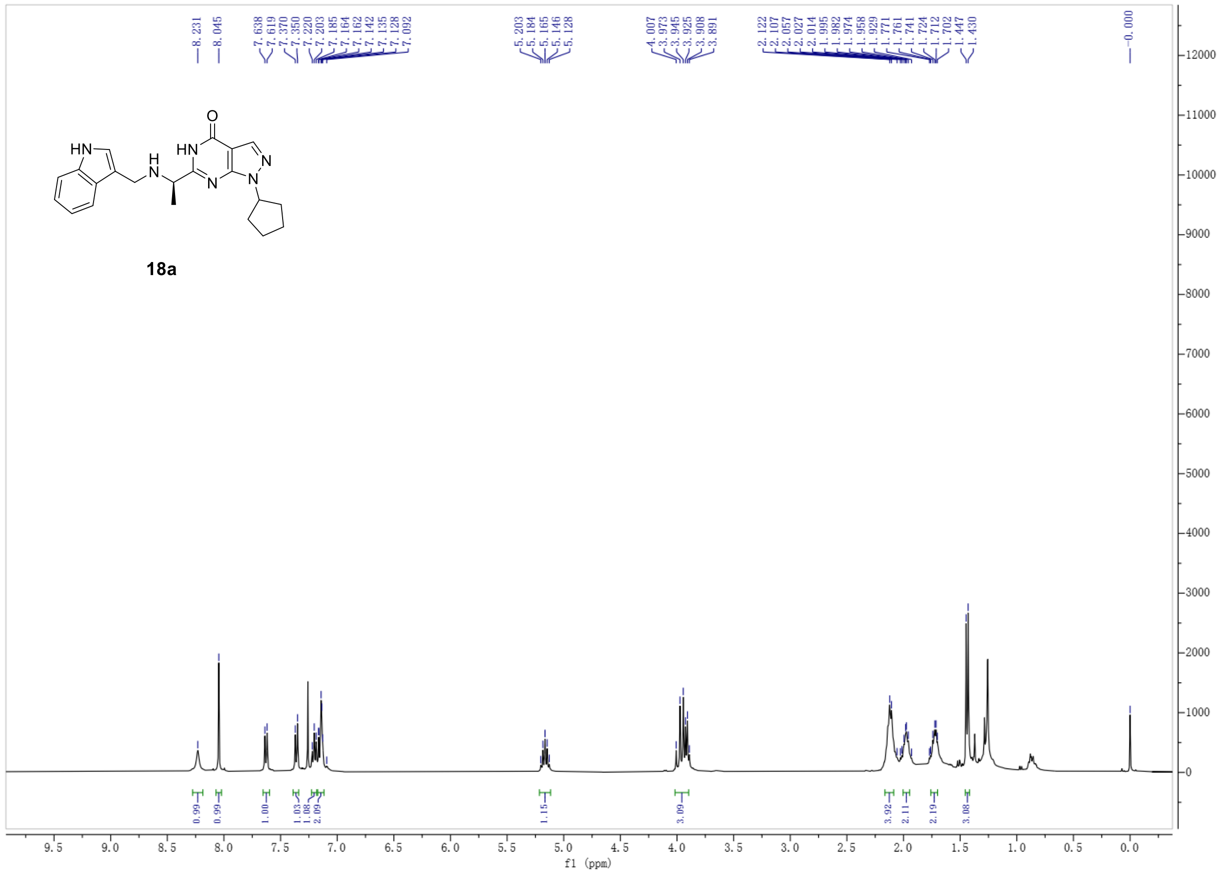

8.231
8.045
7.638
7.619
7.370
7.350
7.220
7.203
7.185
7.164
7.162
7.142
7.135
7.128
7.092
5.203
5.184
5.165
5.146
5.128
4.007
3.973
3.945
3.925
3.908
3.891
2.122
2.107
2.057
2.027
2.014
1.995
1.982
1.974
1.958
1.929
1.771
1.761
1.741
1.724
1.712
1.702
1.447
1.430
0.000
HN
H
N
HN
O
N
N
N
18a
0.99
0.99
1.00
1.03
1.08
2.09
1.15
3.09
3.92
2.11
2.19
3.08
f1 (ppm)

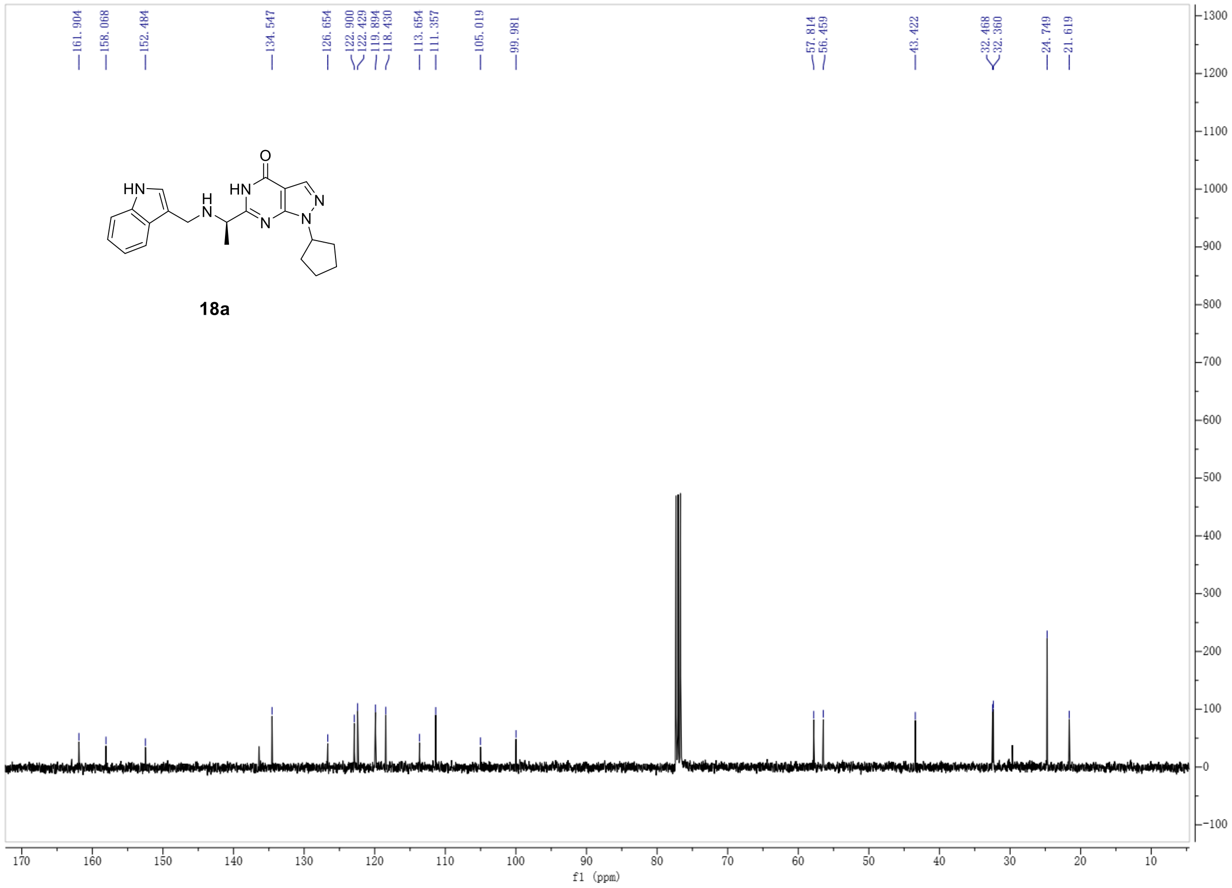

161.904
158.068
152.484
134.547
126.654
122.900
122.429
119.894
118.430
113.654
111.357
105.019
99.981
57.814
56.459
43.422
32.468
32.360
24.749
21.619
HN
H
N
HN
O
N
N
N
18a
f1 (ppm)

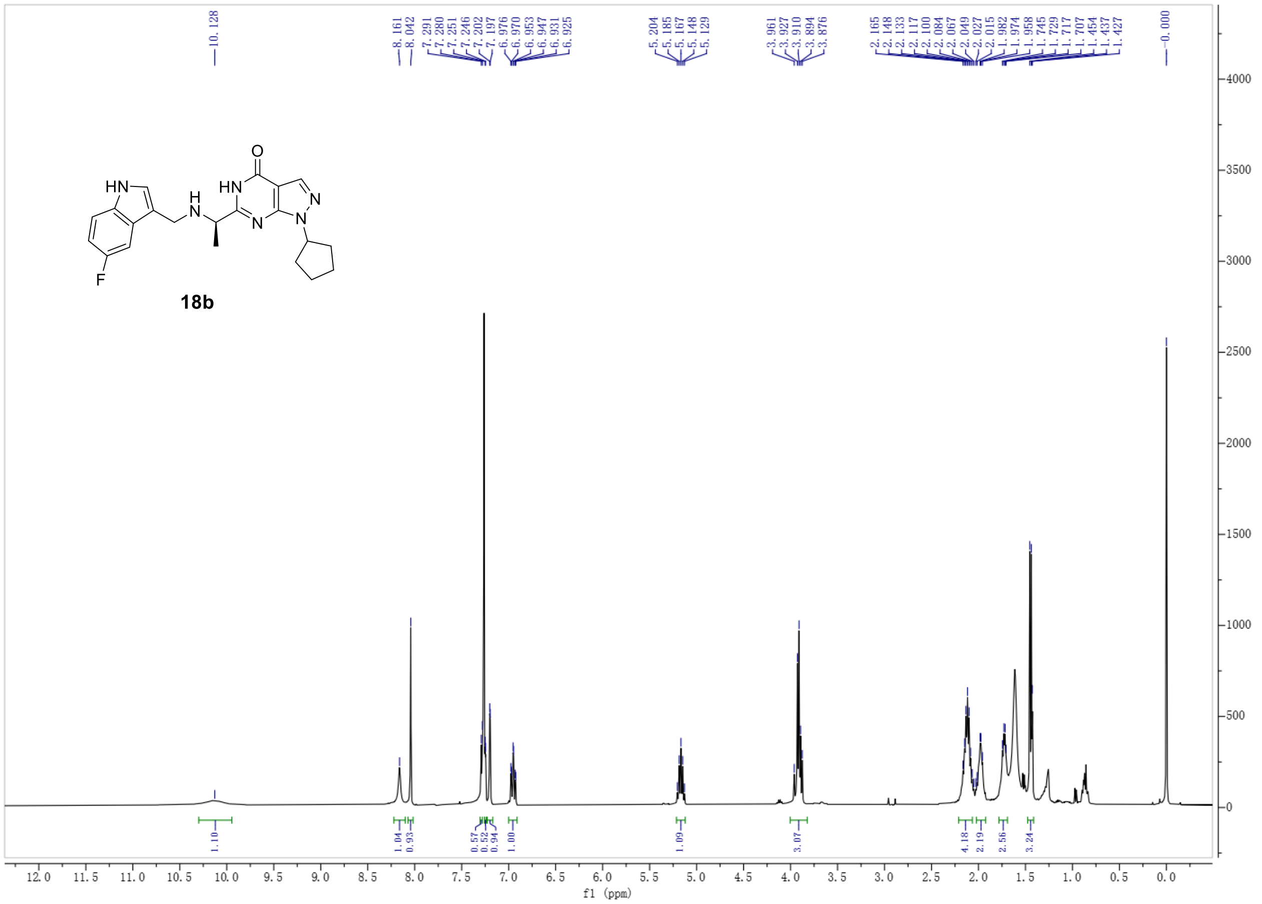
18b
f1 (ppm)

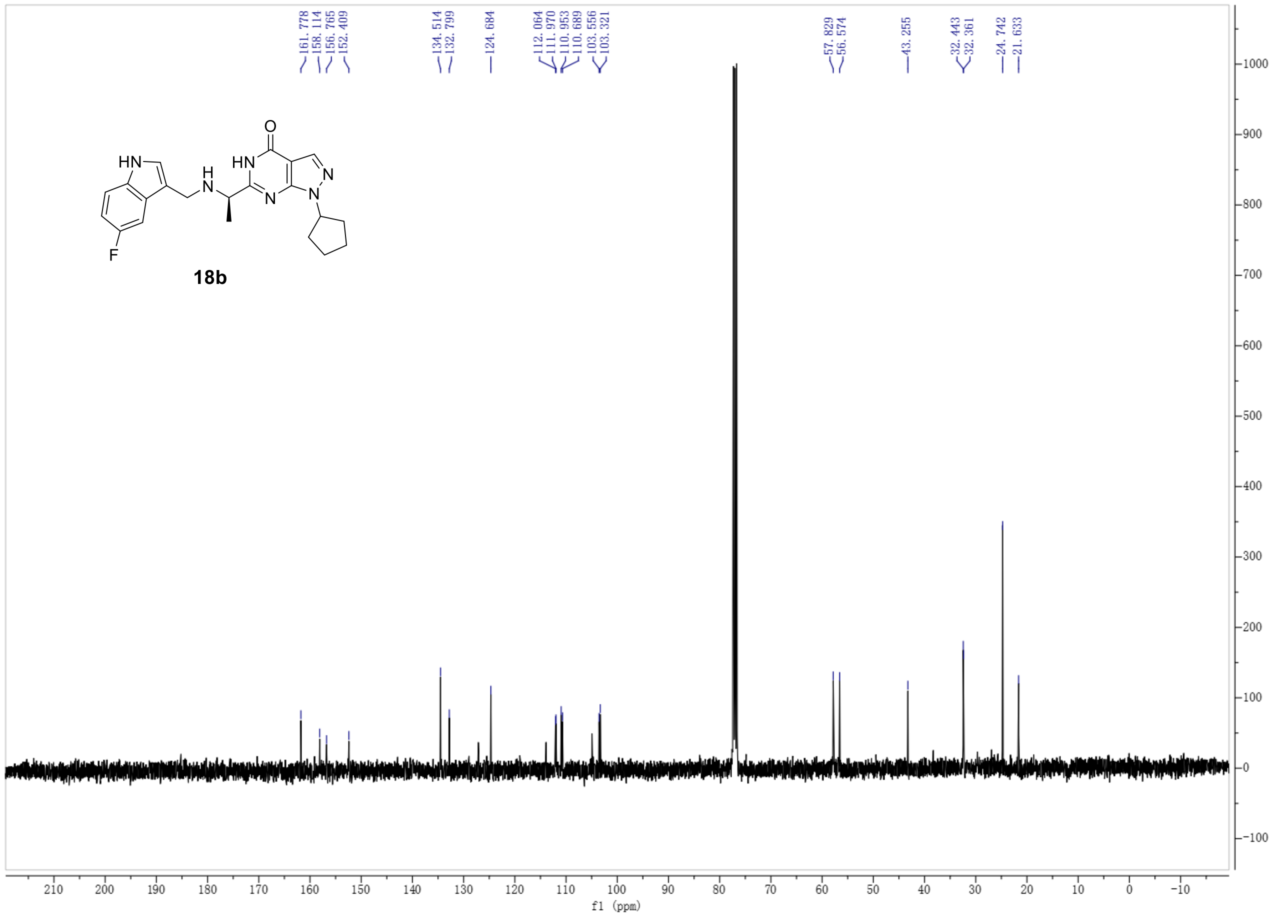
18b
f1 (ppm)

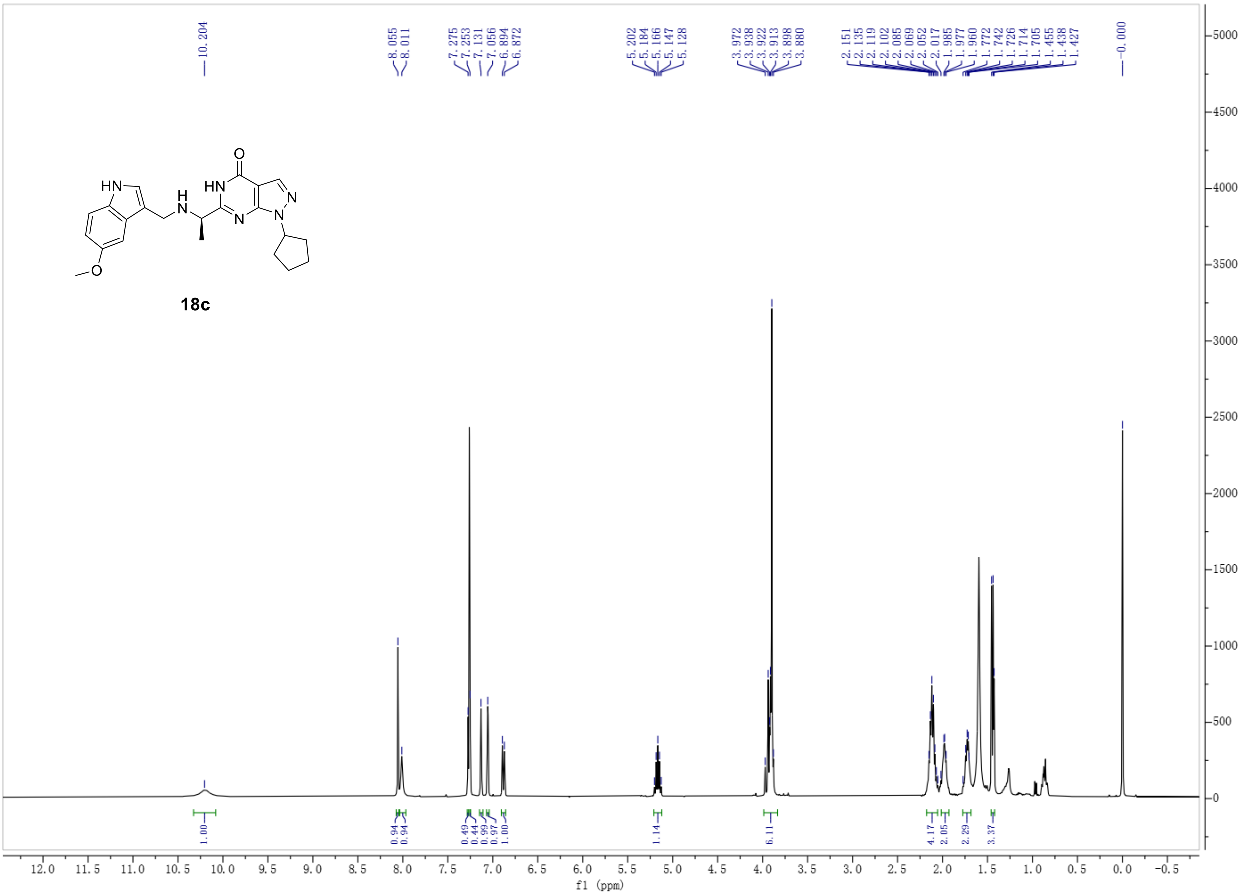
10.204
8.055
8.011
7.275
7.253
7.131
7.056
6.894
6.872
5.202
5.184
5.166
5.147
5.128
3.972
3.938
3.922
3.913
3.898
3.880
2.151
2.135
2.119
2.102
2.085
2.069
2.052
2.017
1.985
1.977
1.960
1.772
1.742
1.726
1.714
1.705
1.455
1.438
1.427
-0.000
18c
f1 (ppm)

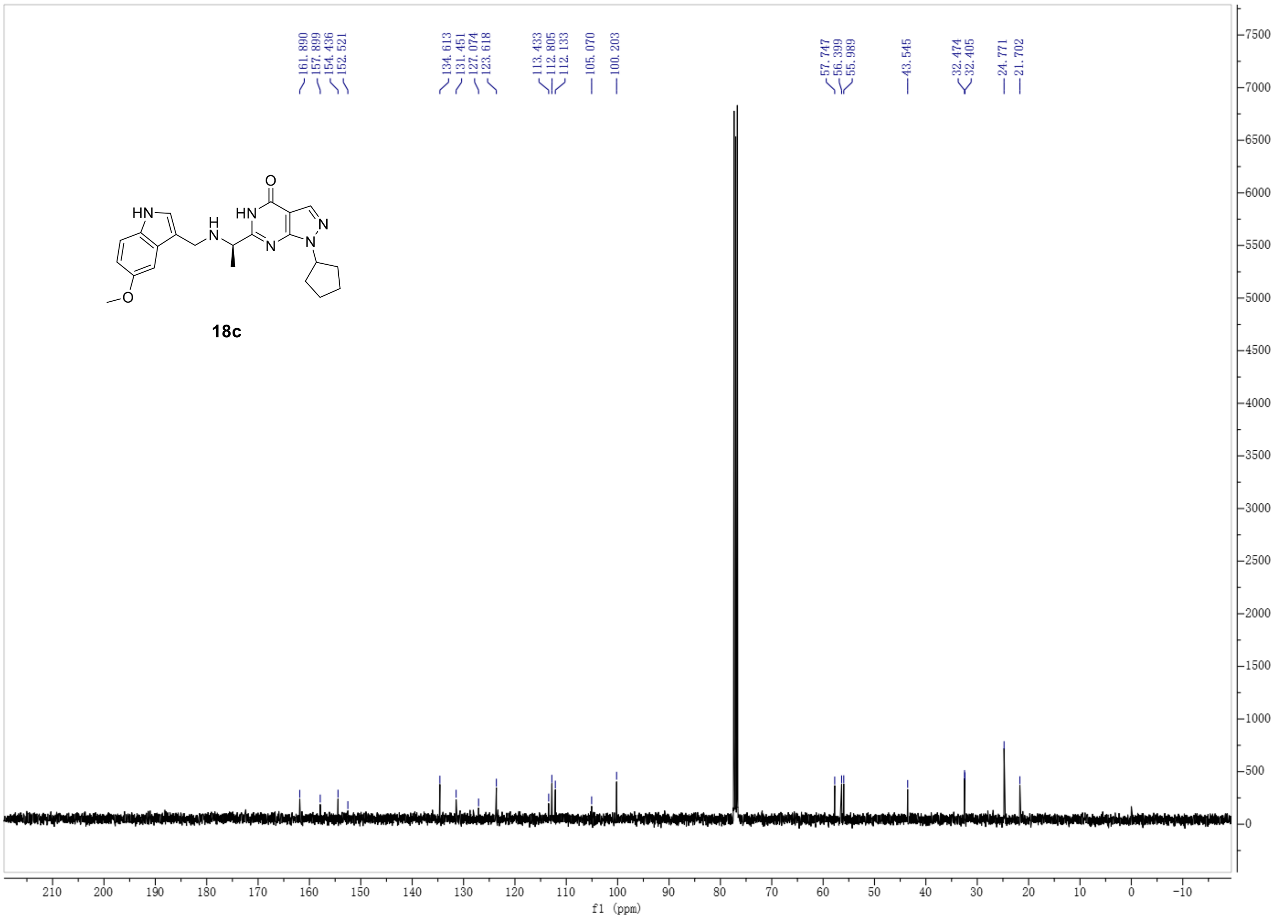
161.890
157.899
154.436
152.521
134.613
131.451
127.074
123.618
113.433
112.805
112.133
105.070
100.203
57.747
56.399
55.989
43.545
32.474
32.405
24.771
21.702
18c
f1 (ppm)

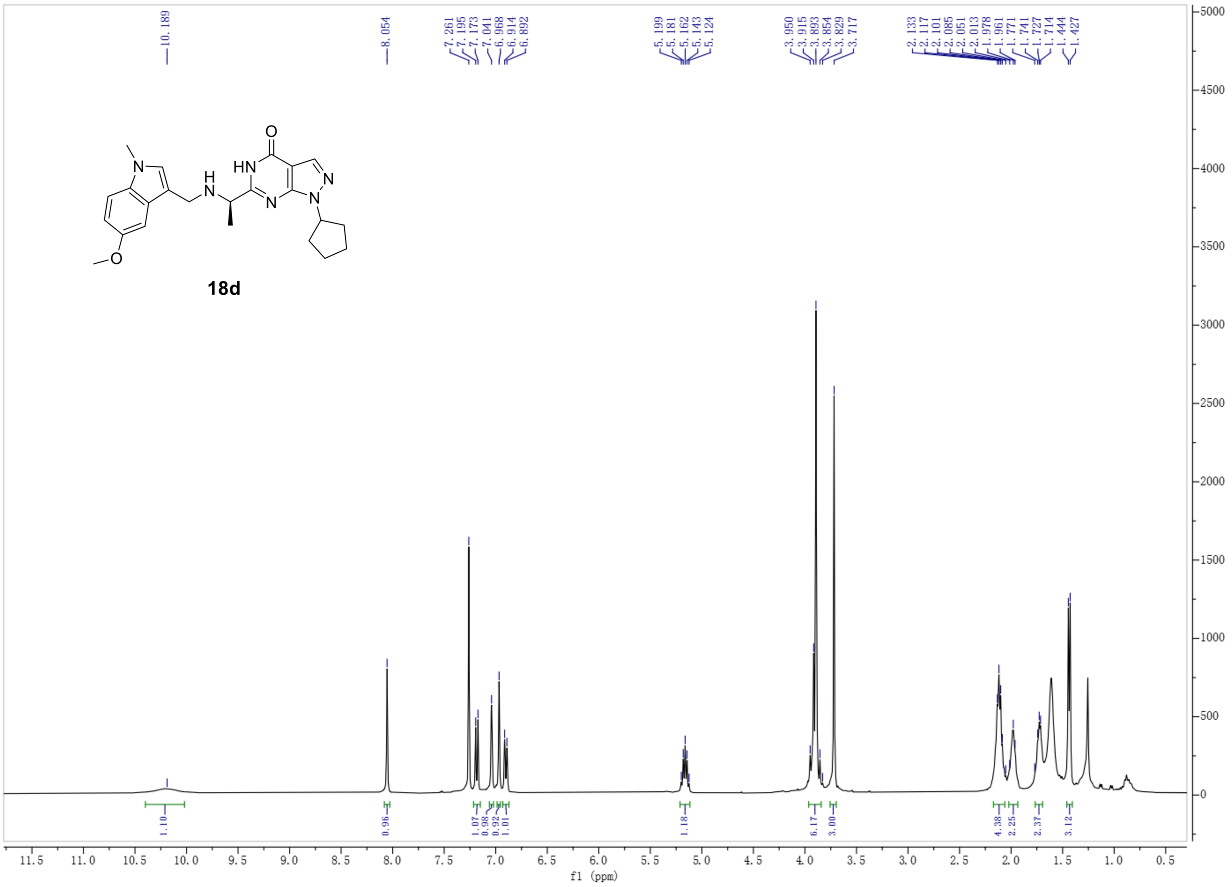
18d
10.189
8.054
7.261 7.195 7.173 7.041 6.968 6.914 6.892
5.199 5.181 5.162 5.143 5.124
3.950 3.915 3.893 3.854 3.829 3.717
2.133 2.117 2.101 2.085 2.051 2.013 1.978 1.961 1.771 1.741 1.727 1.714 1.444 1.427
1.10 0.96 1.07 0.98 0.92 1.01 1.18 6.17 3.00 4.38 2.25 2.37 3.12
f1 (ppm)

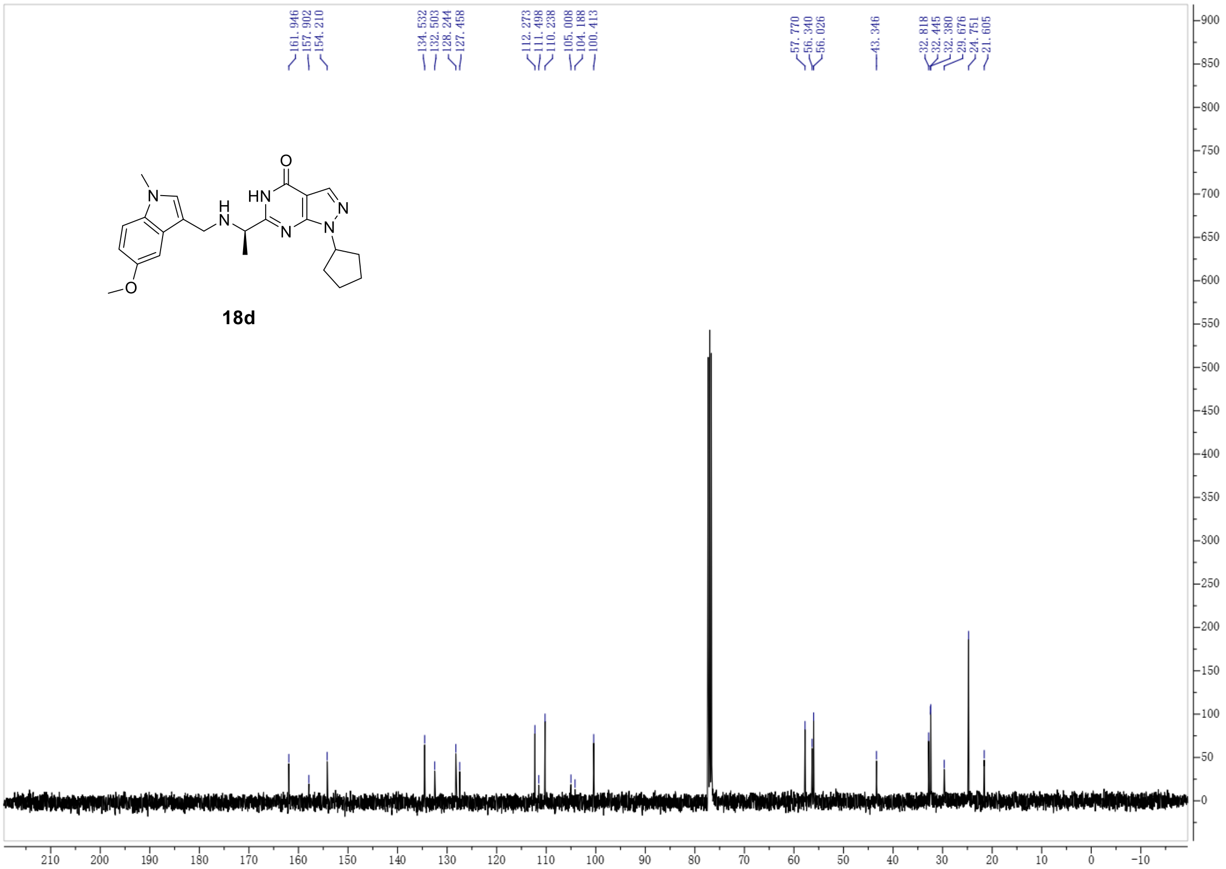
18d
161.946 157.902 154.210
134.532 132.503 128.244 127.458
112.273 111.498 110.238 105.008 104.188 100.413
57.770 56.340 56.026
43.346
32.818 32.445 32.380 29.676 24.751 21.605
f1 (ppm)

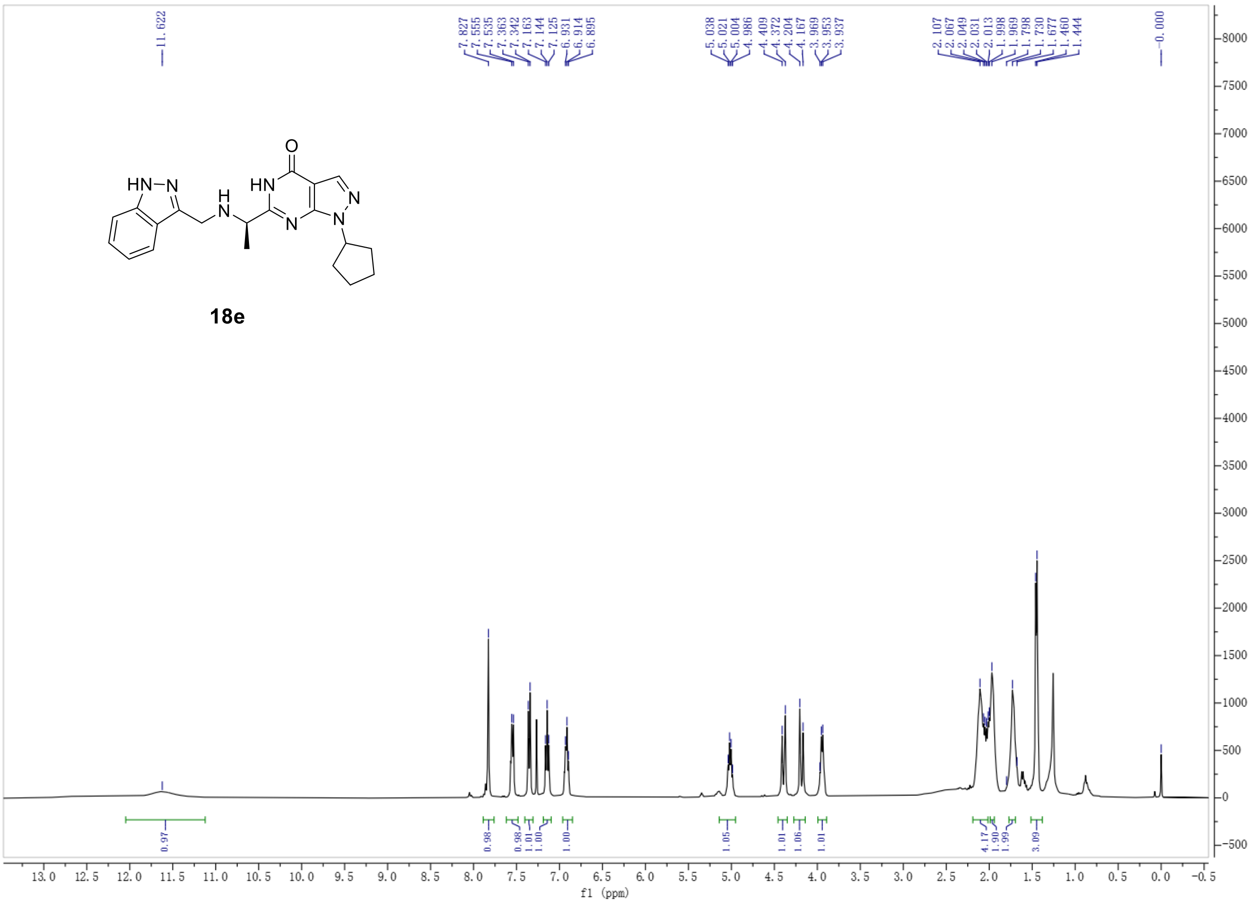
11.622
7.827
7.555
7.535
7.363
7.342
7.163
7.144
7.125
6.931
6.914
6.895
5.038
5.021
5.004
4.986
4.409
4.372
4.204
4.167
3.969
3.953
3.937
2.107
2.067
2.049
2.031
2.013
1.998
1.969
1.798
1.730
1.677
1.460
1.444
0.000
18e
f1 (ppm)

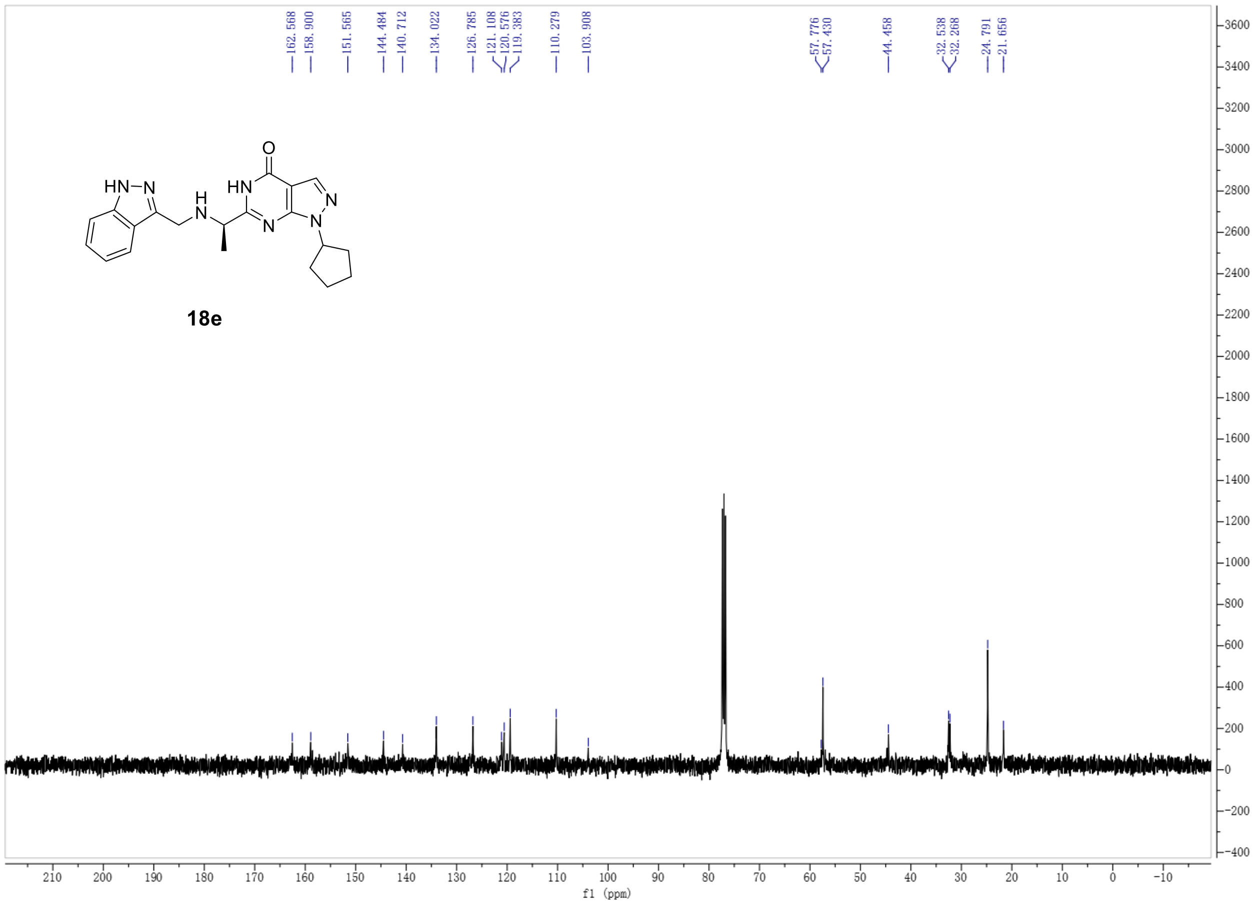
162.568
158.900
151.565
144.484
140.712
134.022
126.785
121.108
120.576
119.383
110.279
103.908
57.776
57.430
44.458
32.538
32.268
24.791
21.656
18e
f1 (ppm)

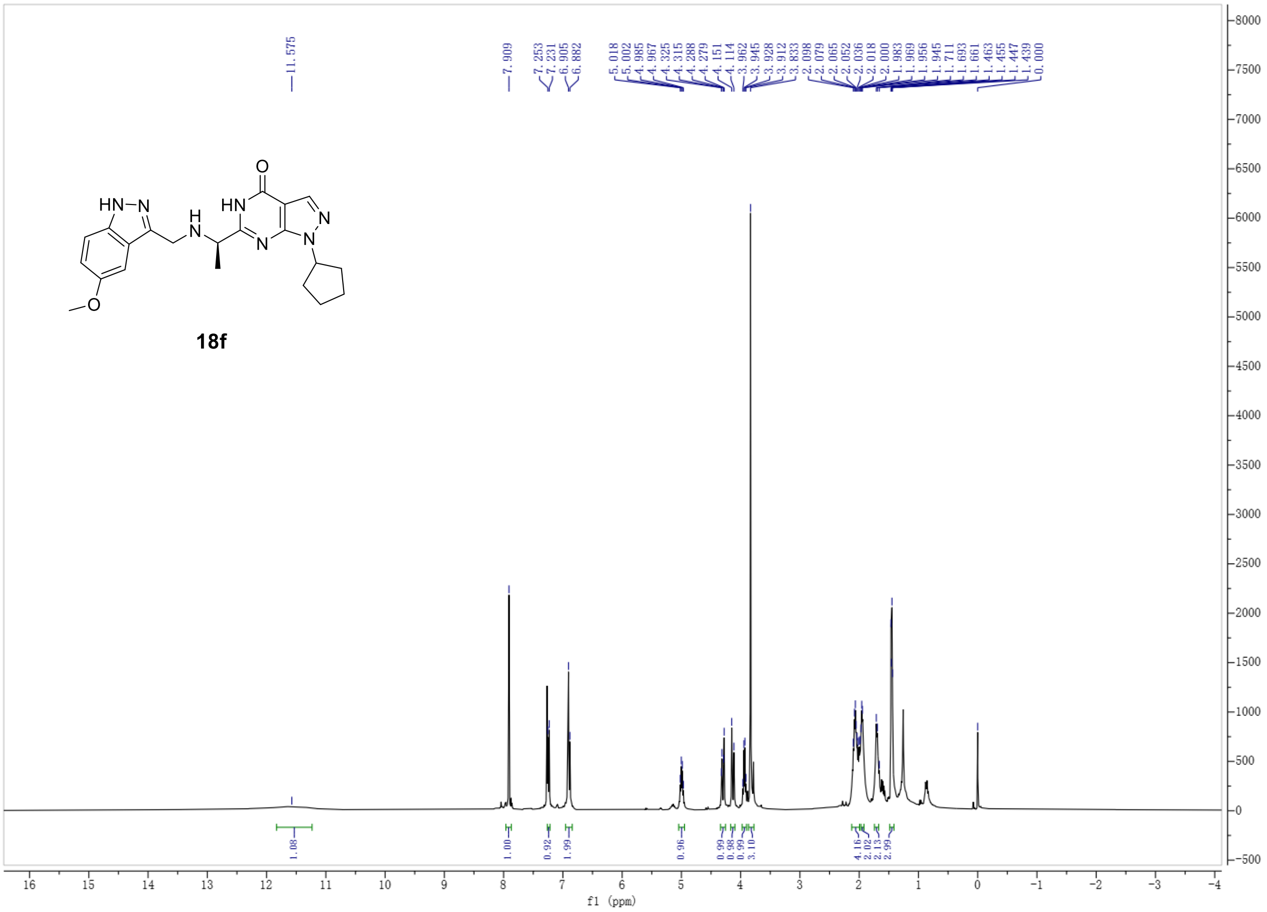

18f

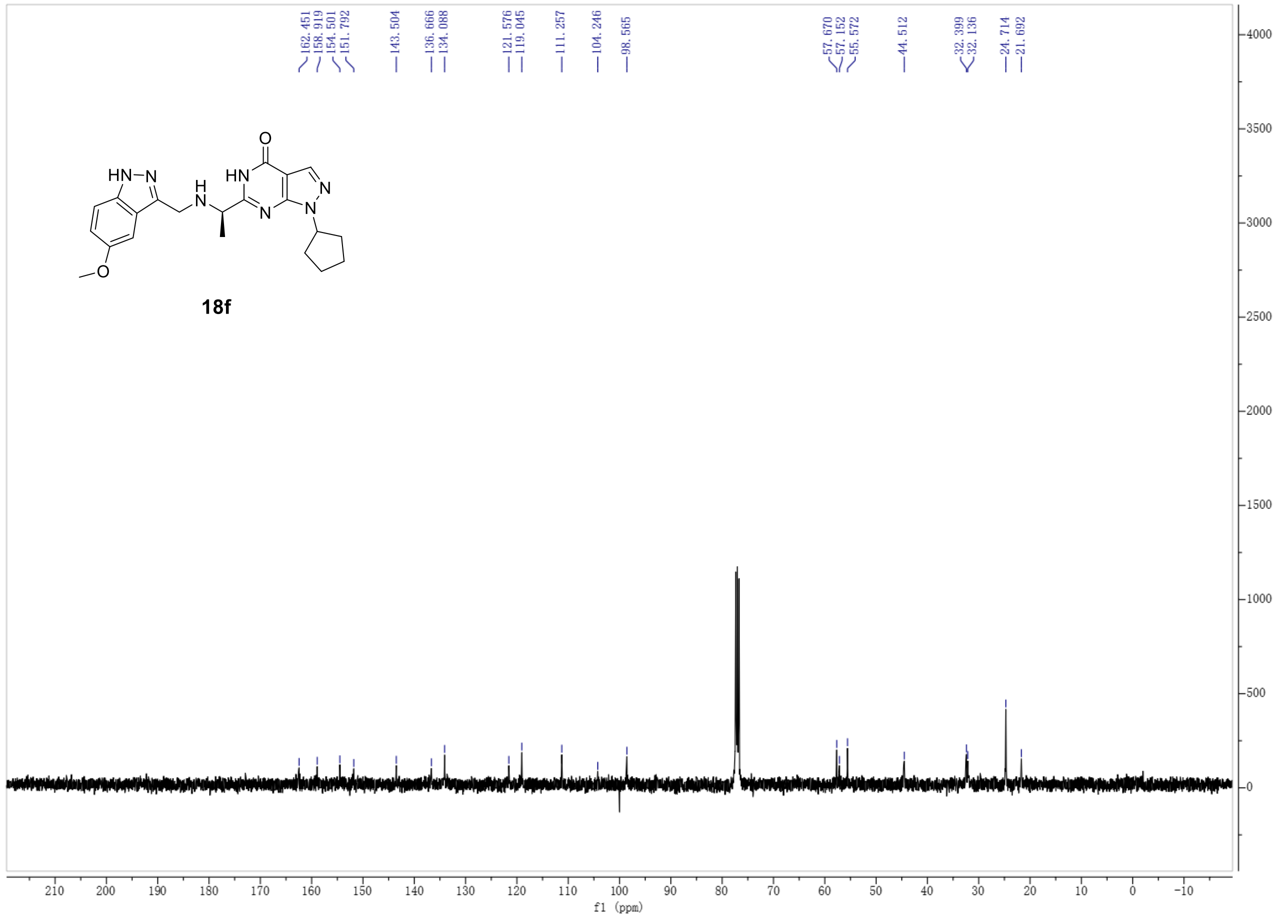

18f

2. The HRMS spectrums of the targeted compounds.

Elmt	Val.	Min	Max	Elmt	Val.	Min	Max	Elmt	Val.	Min	Max	Elmt	Val.	Min	Max	Use Adduct
H	1	0	50	O	2	0	13	P	3	0	0	Cu	2	0	0	H
B	3	0	0	18O	2	0	0	S	2	0	0	Br	1	0	2	Na
C	4	0	50	F	1	0	0	Cl	1	0	0	I	3	0	0	
N	3	0	6	Si	4	0	0	Ni	2	0	0					

Error Margin (ppm):	100	DBE Range:	-2.0 - 1000.0	Electron Ions:	both
HC Ratio:	unlimited	Apply N Rule:	yes	Use MSn Info:	no
Max Isotopes:	all	Isotope RI (%):	1.00	Isotope Res:	10000
MSn Iso RI (%):	75.00	MSn Logic Mode:	AND	Max Results:	500

Event#: 1 MS(E+) Ret. Time : 1.067 - 1.307 -> 1.546 Scan# : 161 - 197 -> 233

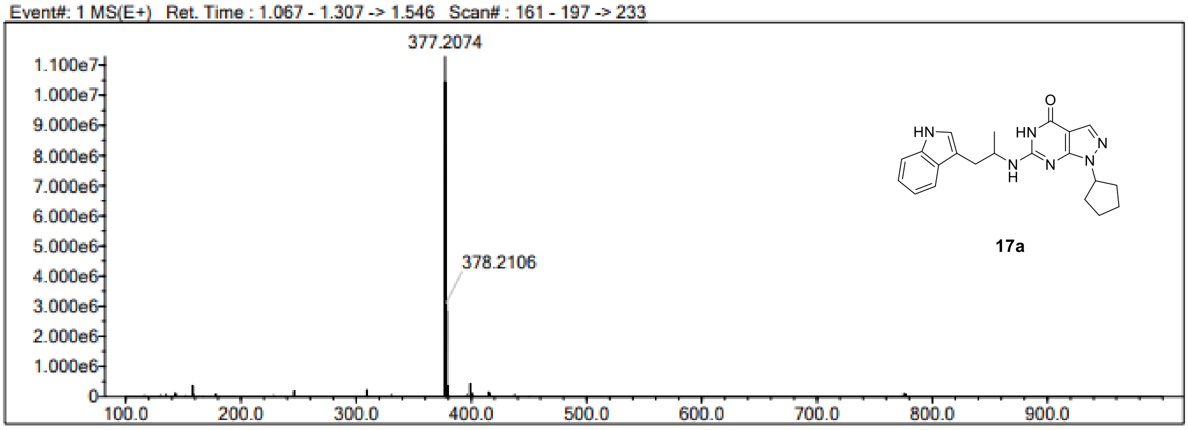

Measured region for 377.2074 m/z

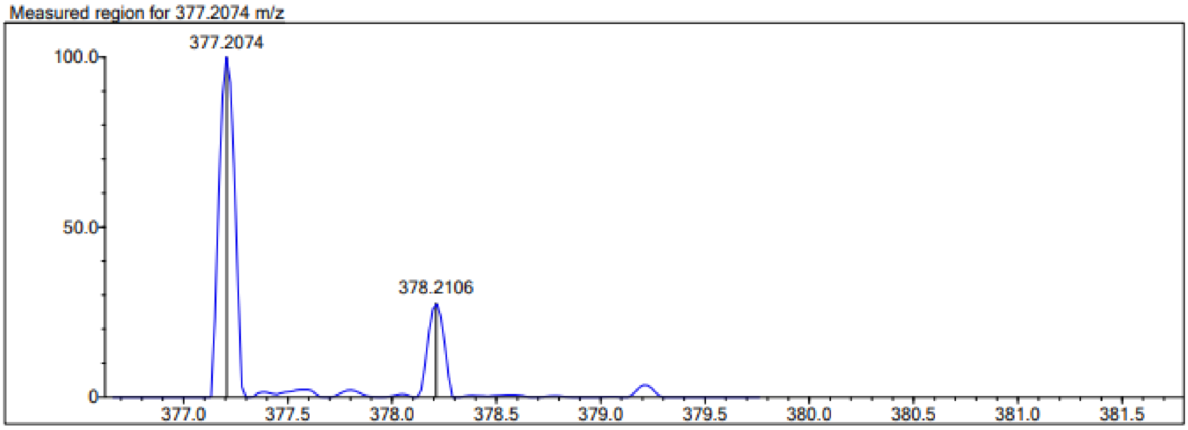

C21 H24 N6 O [M+H]+ : Predicted region for 377.2084 m/z

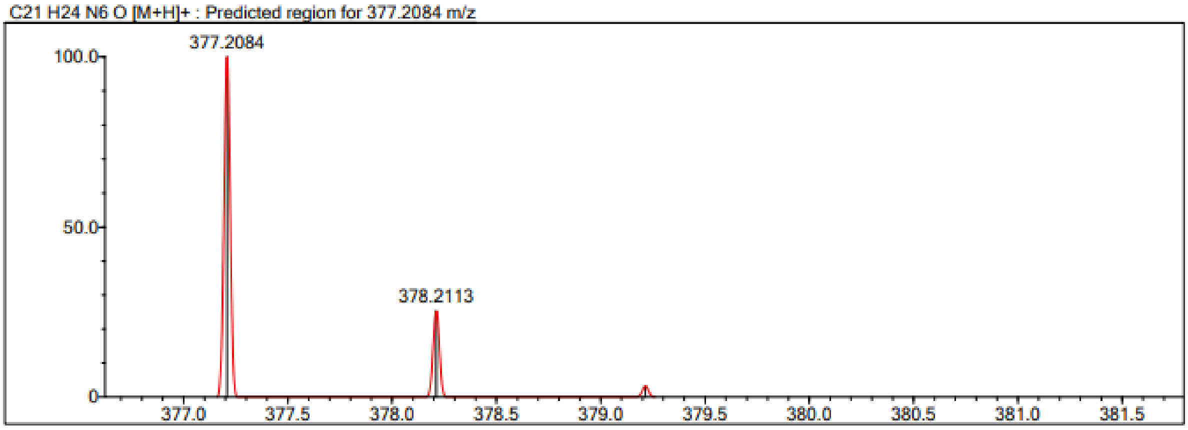

Rank	Score	Formula (M)	Ion	Meas. m/z	Pred. m/z	Df. (mDa)	Df. (ppm)	Iso	DBE
2	73.22	C21 H24 N6 O	[M+H]+	377.2074	377.2084	-1.0	-2.65	76.37	13.0

Elmt	Val.	Min	Max	Elmt	Val.	Min	Max	Elmt	Val.	Min	Max	Elmt	Val.	Min	Max	Use Adduct
H	1	0	27	N	3	0	6	P	3	0	0	Cu	2	0	0	H
B	3	0	0	O	2	0	5	S	2	0	2	Br	1	0	0	Na
C	4	0	28	F	1	0	1	Cl	1	0	1	I	3	0	0	

Error Margin (mDa):	20.0	DBE Range:	-2.0 - 1000.0	Electron Ions:	both
HC Ratio:	unlimited	Apply N Rule:	yes	Use MSn Info:	yes
Max Isotopes:	all	Isotope RI (%):	1.00	Isotope Res:	10000
MSn Iso RI (%):	75.00	MSn Logic Mode:	AND	Max Results:	800

Event#: 1 MS(E+) Ret. Time : 1.622 -> 1.622 Scan# : 325 -> 325

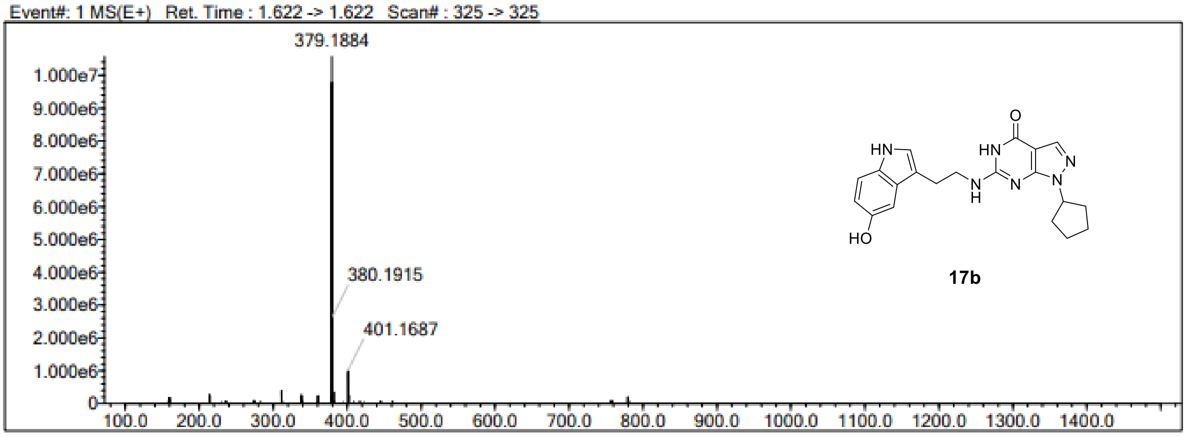

Measured region for 379.1884 m/z

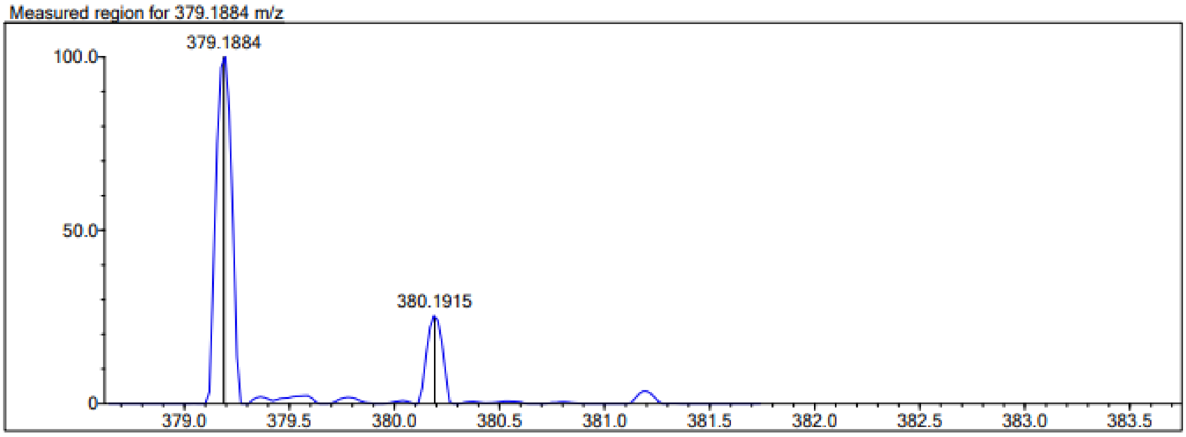

C20 H22 N6 O2 [M+H]+ : Predicted region for 379.1877 m/z

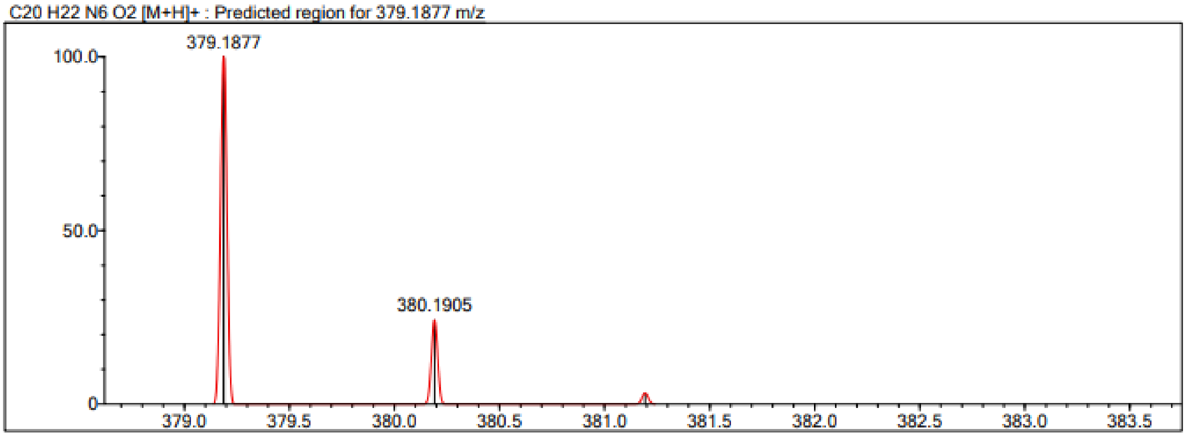

Rank	Score	Formula (M)	Ion	Meas. m/z	Pred. m/z	Df. (mDa)	Df. (ppm)	Iso	DBE
1	89.33	C20 H22 N6 O2	[M+H]+	379.1884	379.1877	0.7	1.85	91.27	13.0

Elmt	Val.	Min	Max	Elmt	Val.	Min	Max	Elmt	Val.	Min	Max	Elmt	Val.	Min	Max	Use Adduct
H	1	0	27	N	3	0	6	P	3	0	0	Cu	2	0	0	H
B	3	0	0	O	2	0	5	S	2	0	2	Br	1	0	0	Na
C	4	0	28	F	1	0	1	Cl	1	0	1	I	3	0	0	

Error Margin (mDa): 20.0
HC Ratio: unlimited
Max Isotopes: all
MSn Iso RI (%): 75.00

DBE Range: -2.0 - 1000.0
Apply N Rule: yes
Isotope RI (%): 1.00
MSn Logic Mode: AND

Electron Ions: both
Use MSn Info: yes
Isotope Res: 10000
Max Results: 800

Event#: 1 MS(E+) Ret. Time : 2.587 -> 2.587 Scan# : 518 -> 518

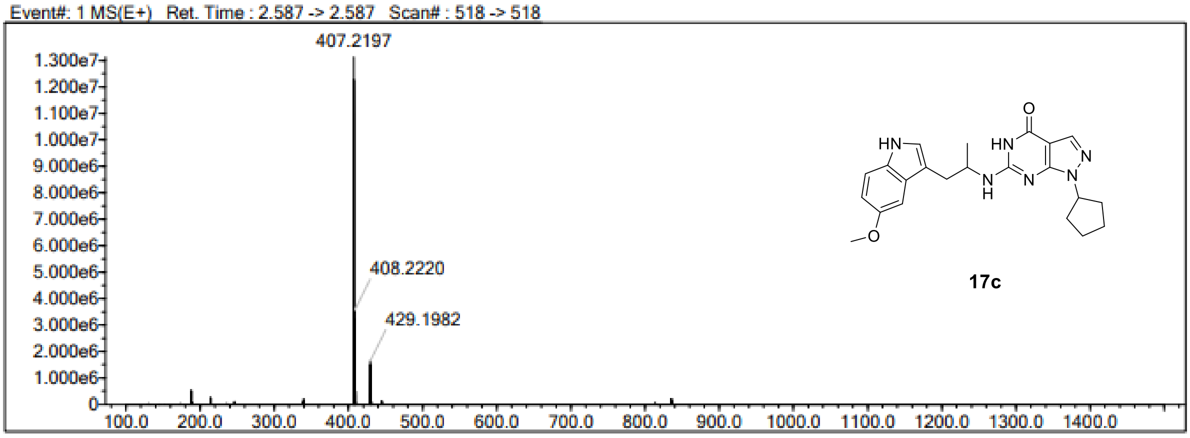

17c

Measured region for 407.2197 m/z

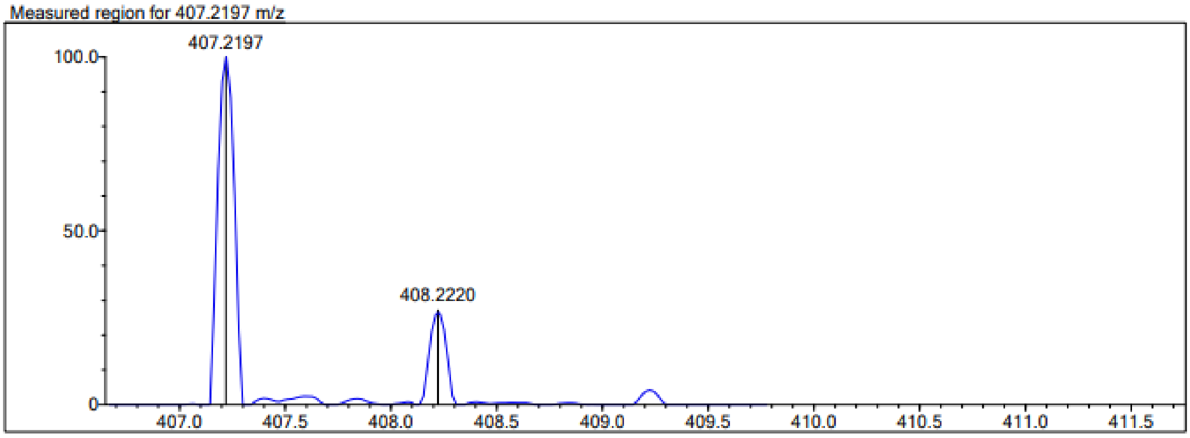

C22 H26 N6 O2 [M+H]+ : Predicted region for 407.2190 m/z

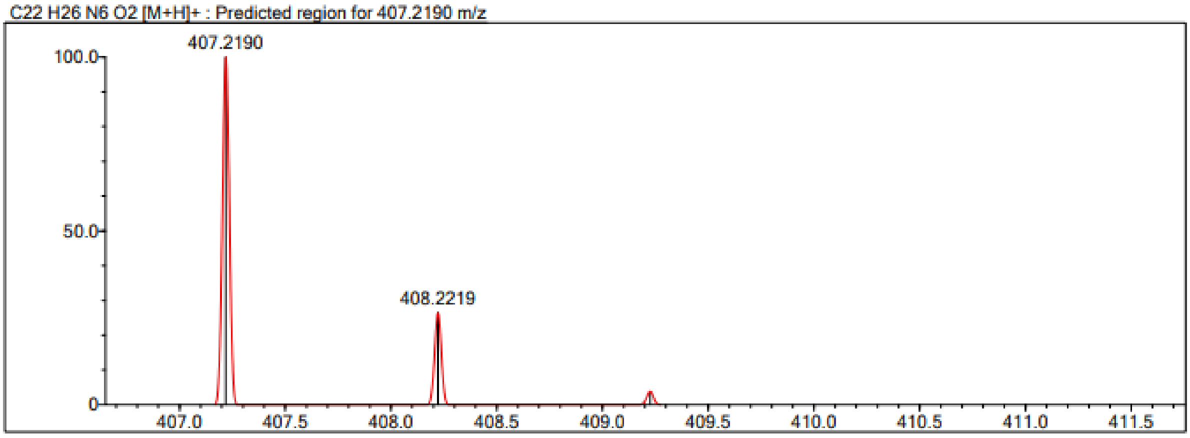

Rank	Score	Formula (M)	Ion	Meas. m/z	Pred. m/z	Df. (mDa)	Df. (ppm)	Iso	DBE
2	80.42	C22 H26 N6 O2	[M+H]+	407.2197	407.2190	0.7	1.72	81.89	13.0

Elmt	Val.	Min	Max	Elmt	Val.	Min	Max	Elmt	Val.	Min	Max	Elmt	Val.	Min	Max	Use Adduct
H	1	0	27	N	3	0	6	P	3	0	0	Cu	2	0	0	H
B	3	0	0	O	2	0	5	S	2	0	2	Br	1	0	0	Na
C	4	0	28	F	1	0	1	Cl	1	0	1	I	3	0	0	

Error Margin (mDa):	20.0	DBE Range:	-2.0 - 1000.0	Electron Ions:	both
HC Ratio:	unlimited	Apply N Rule:	yes	Use MSn Info:	yes
Max Isotopes:	all	Isotope RI (%):	1.00	Isotope Res:	10000
MSn Iso RI (%):	75.00	MSn Logic Mode:	AND	Max Results:	800

Event#: 1 MS(E+) Ret. Time : 1.692 -> 1.692 Scan# : 339 -> 339

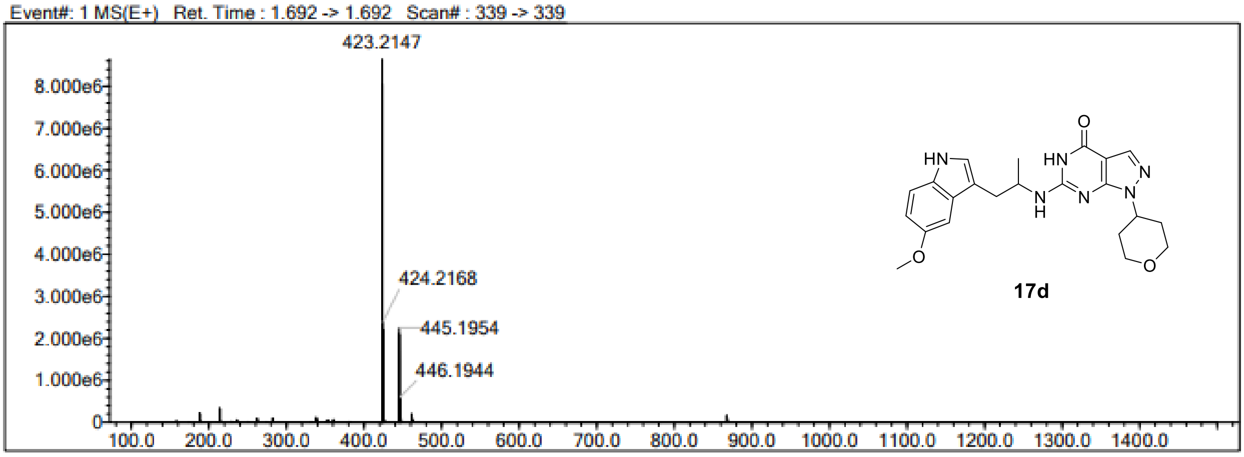

17d

Measured region for 423.2147 m/z

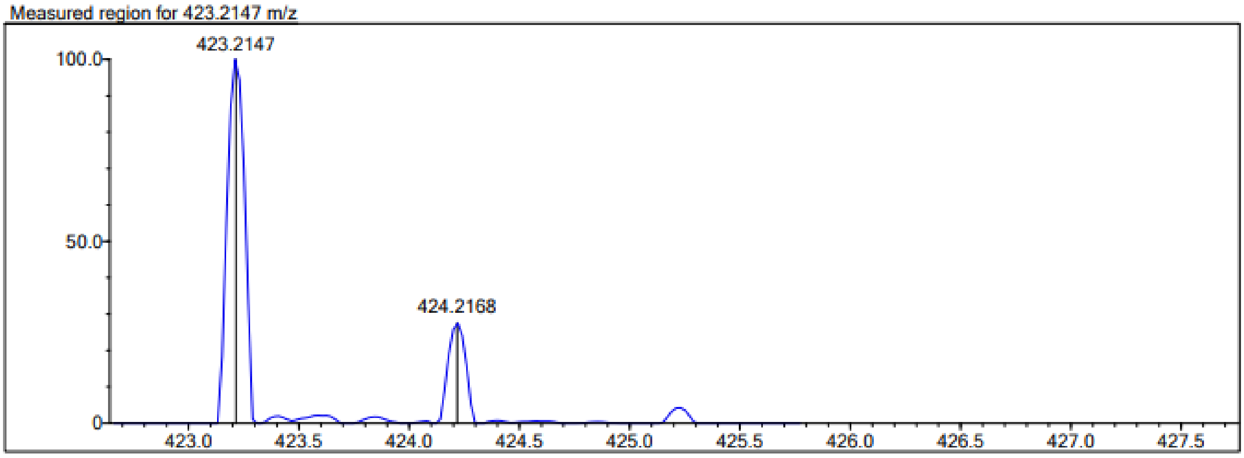

C22 H26 N6 O3 [M+H]+ : Predicted region for 423.2139 m/z

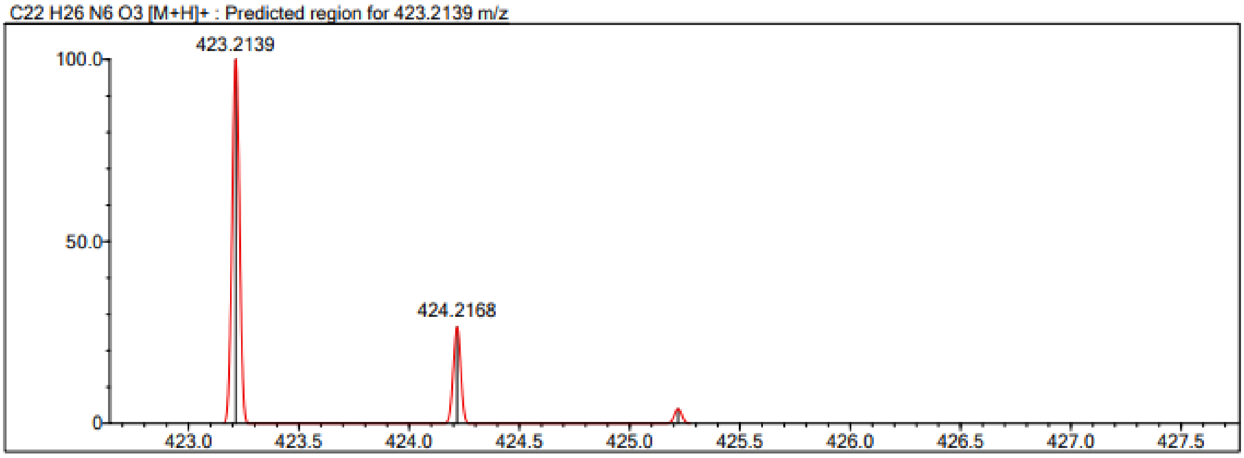

Rank	Score	Formula (M)	Ion	Meas. m/z	Pred. m/z	Df. (mDa)	Df. (ppm)	Iso	DBE
1	93.64	C22 H26 N6 O3	[M+H]+	423.2147	423.2139	0.8	1.89	95.77	13.0

Elmt	Val.	Min	Max	Elmt	Val.	Min	Max	Elmt	Val.	Min	Max	Elmt	Val.	Min	Max	Use Adduct
H	1	0	27	N	3	0	6	P	3	0	0	Cu	2	0	0	H
B	3	0	0	O	2	0	5	S	2	0	2	Br	1	0	0	Na
C	4	0	28	F	1	0	1	Cl	1	0	1	I	3	0	0	

Error Margin (mDa): 20.0
HC Ratio: unlimited
Max Isotopes: all
MSn Iso RI (%): 75.00

DBE Range: -2.0 - 1000.0
Apply N Rule: yes
Isotope RI (%): 1.00
MSn Logic Mode: AND

Electron Ions: both
Use MSn Info: yes
Isotope Res: 10000
Max Results: 800

Event#: 1 MS(E+) Ret. Time : 3.237 -> 3.237 Scan# : 648 -> 648

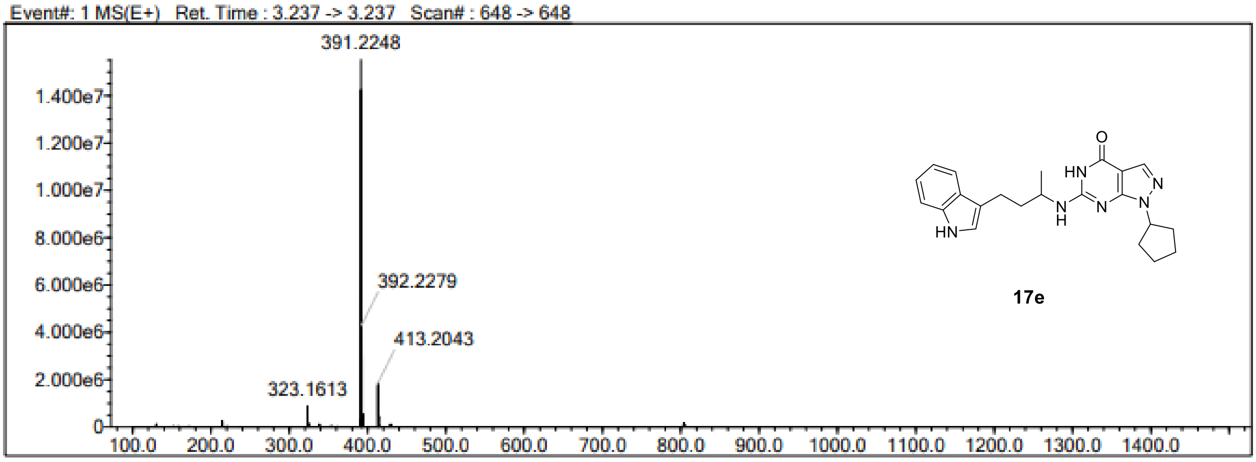

Measured region for 391.2248 m/z

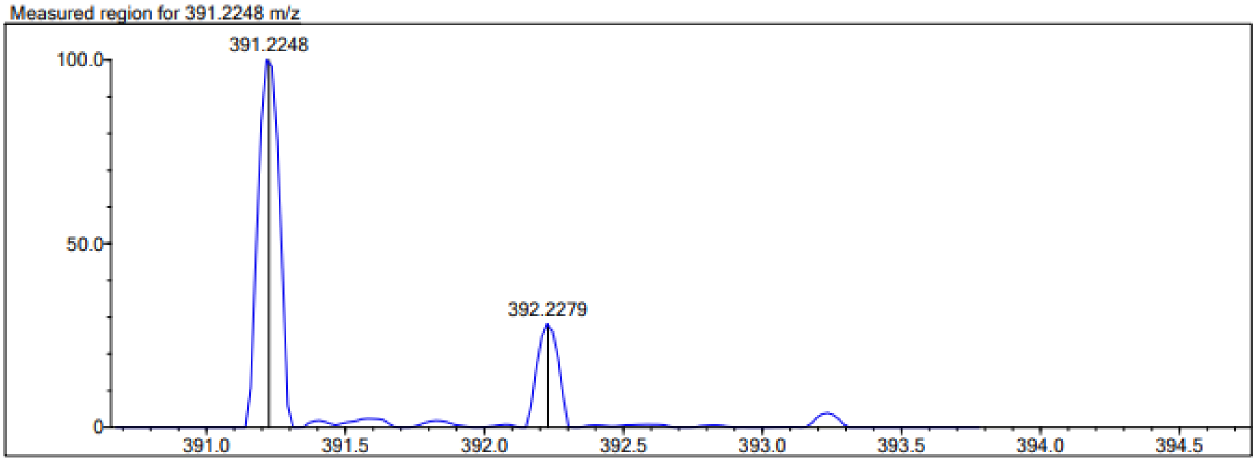

C22 H26 N6 O [M+H]+ : Predicted region for 391.2241 m/z

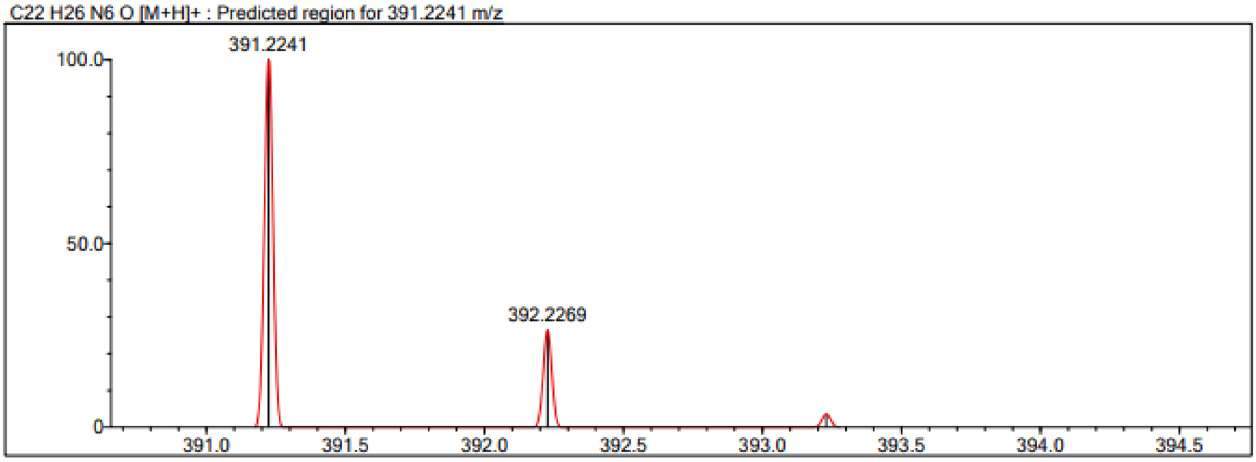

Rank	Score	Formula (M)	Ion	Meas. m/z	Pred. m/z	Df. (mDa)	Df. (ppm)	Iso	DBE
1	88.00	C22 H26 N6 O	[M+H]+	391.2248	391.2241	0.7	1.79	89.78	13.0

Elmt	Val.	Min	Max	Elmt	Val.	Min	Max	Elmt	Val.	Min	Max	Elmt	Val.	Min	Max	Use Adduct
H	1	0	50	O	2	0	13	P	3	0	0	Cu	2	0	0	H
B	3	0	0	18O	2	0	0	S	2	0	0	Br	1	0	2	Na
C	4	0	50	F	1	0	0	Cl	1	0	0	I	3	0	0	
N	3	0	7	Si	4	0	0	Ni	2	0	0					

Error Margin (ppm): 100
HC Ratio: unlimited
Max Isotopes: all
MSn Iso RI (%): 75.00

DBE Range: -2.0 - 1000.0
Apply N Rule: yes
Isotope RI (%): 1.00
MSn Logic Mode: AND

Electron Ions: both
Use MSn Info: no
Isotope Res: 10000
Max Results: 500

Event#: 1 MS(E+) Ret. Time : 1.013 - 1.240 -> 1.379 Scan# : 153 - 187 -> 207

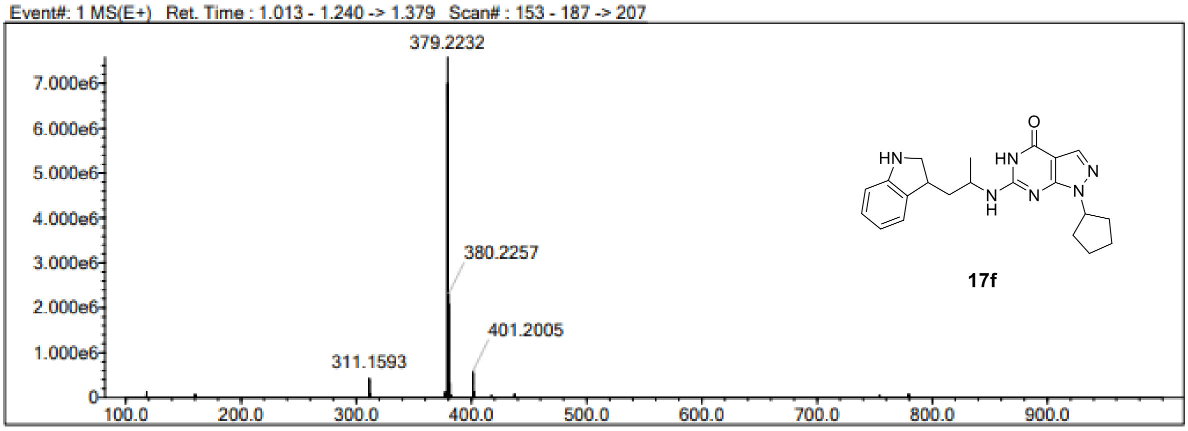

Measured region for 379.2232 m/z

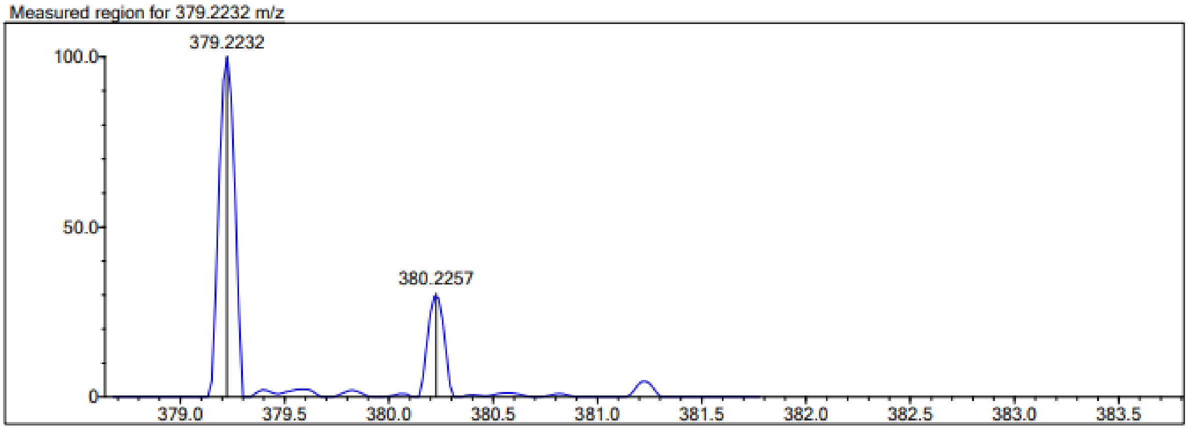

C21 H26 N6 O [M+H]+ : Predicted region for 379.2241 m/z

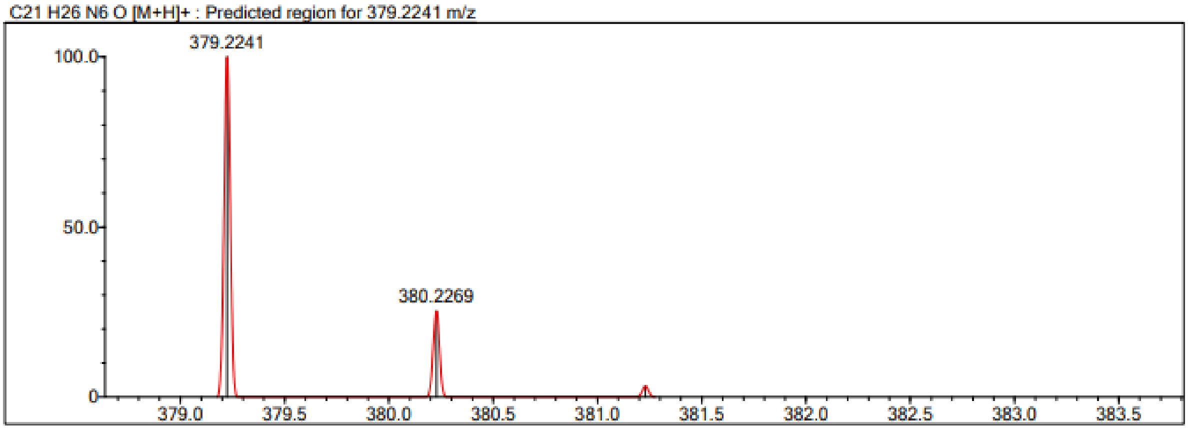

Rank	Score	Formula (M)	Ion	Meas. m/z	Pred. m/z	Df. (mDa)	Df. (ppm)	Iso	DBE
1	72.78	C21 H26 N6 O	[M+H]+	379.2232	379.2241	-0.9	-2.37	75.36	12.0

Elmt	Val.	Min	Max	Elmt	Val.	Min	Max	Elmt	Val.	Min	Max	Elmt	Val.	Min	Max	Use Adduct
H	1	0	50	O	2	0	13	P	3	0	0	Cu	2	0	0	H
B	3	0	0	18O	2	0	0	S	2	0	0	Br	1	0	2	Na
C	4	0	50	F	1	0	0	Cl	1	0	0	I	3	0	0	
N	3	0	7	Si	4	0	0	Ni	2	0	0					

Error Margin (ppm):	100	DBE Range:	-2.0 - 1000.0	Electron Ions:	both
HC Ratio:	unlimited	Apply N Rule:	yes	Use MSn Info:	no
Max Isotopes:	all	Isotope RI (%):	1.00	Isotope Res:	10000
MSn Iso RI (%):	75.00	MSn Logic Mode:	AND	Max Results:	500

Event#: 1 MS(E+) Ret. Time : 1.120 - 1.147 -> 1.290 Scan# : 169 - 173 -> 195

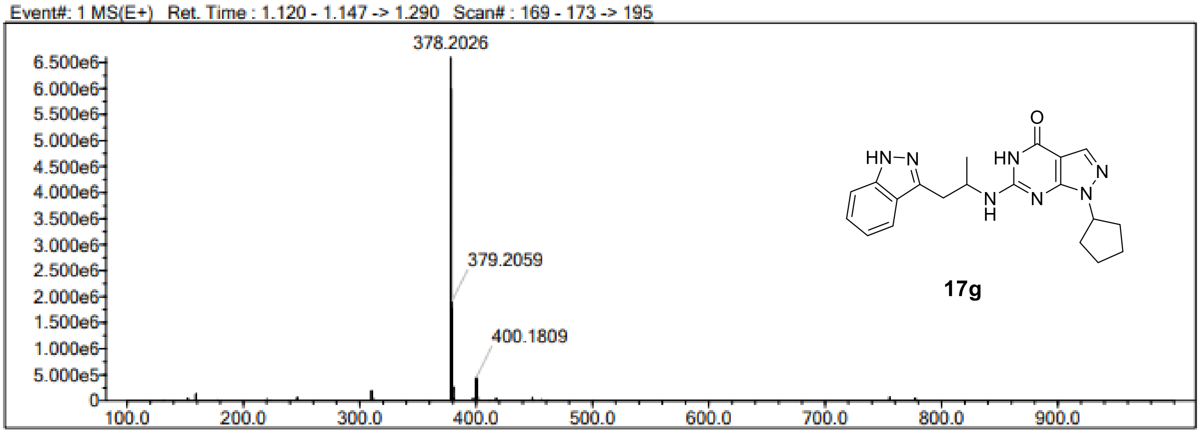

Measured region for 378.2026 m/z

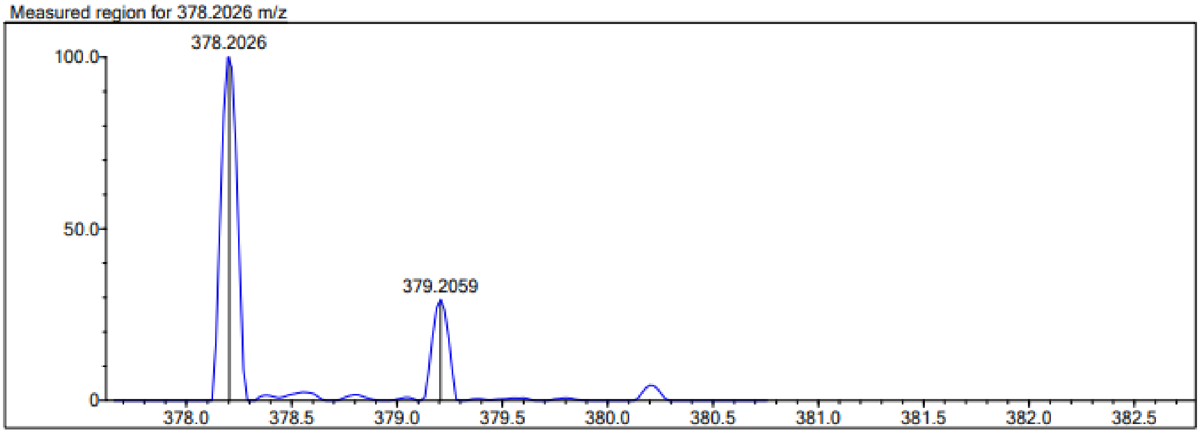

C20 H23 N7 O [M+H]+ : Predicted region for 378.2037 m/z

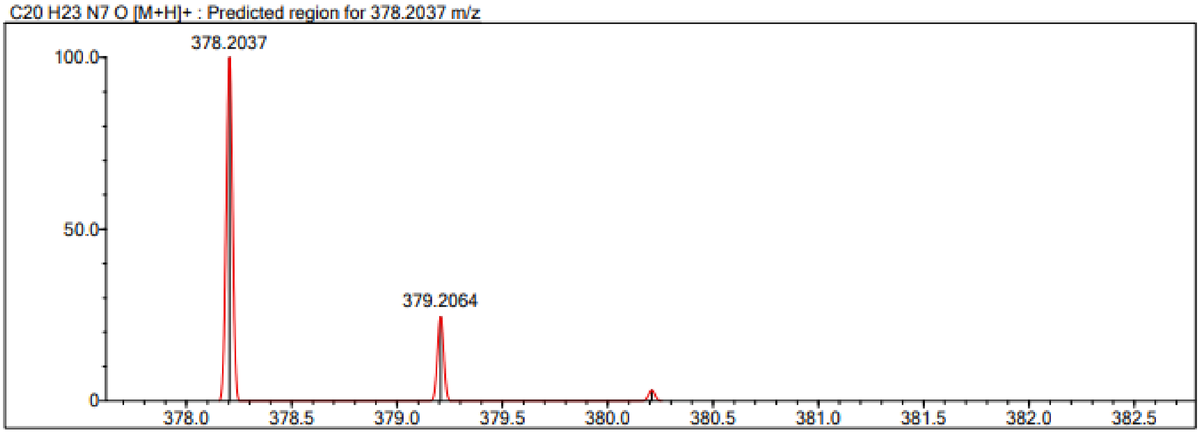

Rank	Score	Formula (M)	Ion	Meas. m/z	Pred. m/z	Df. (mDa)	Df. (ppm)	Iso	DBE
1	76.84	C20 H23 N7 O	[M+H]+	378.2026	378.2037	-1.1	-2.91	80.69	13.0

Elmt	Val.	Min	Max	Elmt	Val.	Min	Max	Elmt	Val.	Min	Max	Elmt	Val.	Min	Max	Use Adduct
H	1	0	50	O	2	0	13	P	3	0	0	Cu	2	0	0	H
B	3	0	0	18O	2	0	0	S	2	0	0	Br	1	0	2	Na
C	4	0	50	F	1	0	1	Cl	1	0	0	I	3	0	0	
N	3	0	7	Si	4	0	0	Ni	2	0	0					

Error Margin (ppm):	100	DBE Range:	-2.0 - 1000.0	Electron Ions:	both
HC Ratio:	unlimited	Apply N Rule:	yes	Use MSn Info:	no
Max Isotopes:	all	Isotope RI (%):	1.00	Isotope Res:	10000
MSn Iso RI (%):	75.00	MSn Logic Mode:	AND	Max Results:	500

Event#: 1 MS(E+) Ret. Time : 0.973 - 1.173 -> 1.409 Scan# : 147 - 177 -> 213

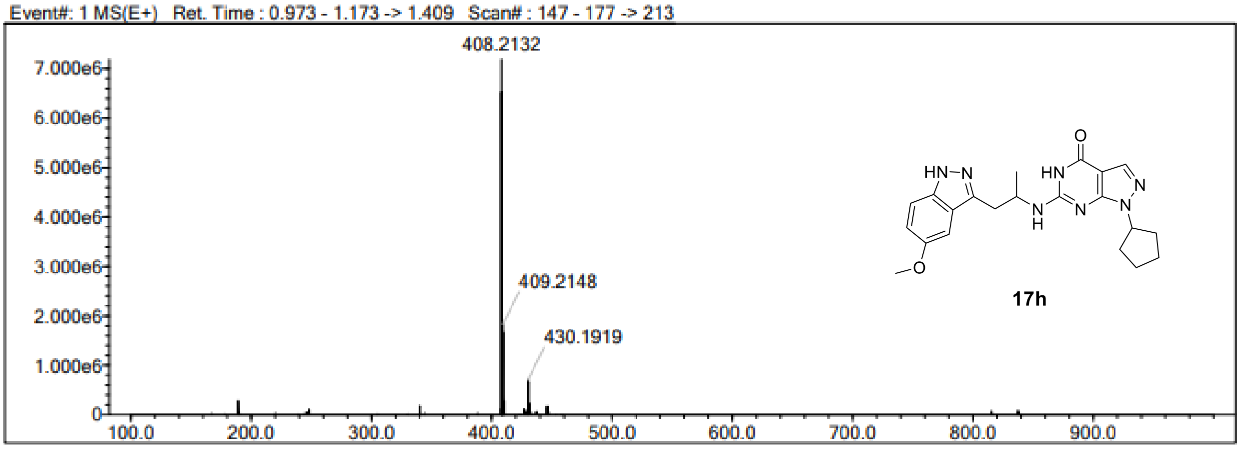

Measured region for 408.2132 m/z

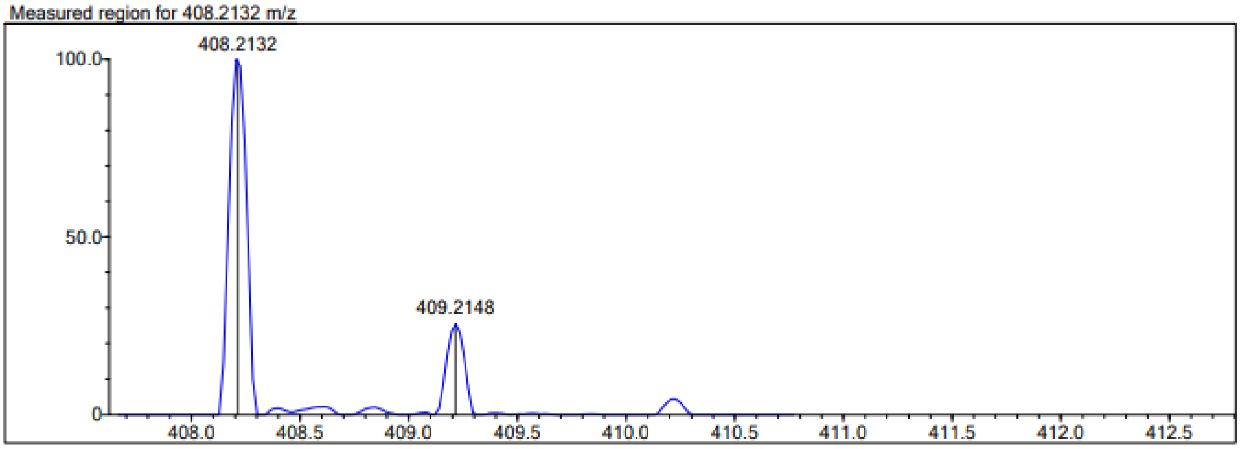

C21 H25 N7 O2 [M+H]+ : Predicted region for 408.2142 m/z

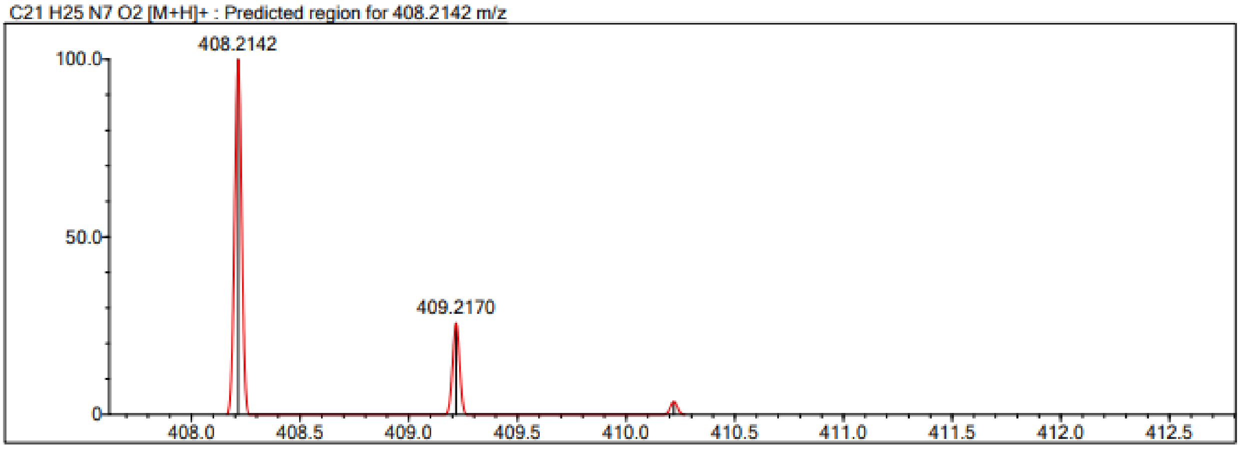

Rank	Score	Formula (M)	Ion	Meas. m/z	Pred. m/z	Df. (mDa)	Df. (ppm)	Iso	DBE
3	86.14	C21 H25 N7 O2	[M+H]+	408.2132	408.2142	-1.0	-2.45	89.38	13.0

Elmt	Val.	Min	Max	Elmt	Val.	Min	Max	Elmt	Val.	Min	Max	Elmt	Val.	Min	Max	Use Adduct
H	1	0	50	O	2	0	13	P	3	0	0	Cu	2	0	0	H
B	3	0	0	18O	2	0	0	S	2	0	0	Br	1	0	2	
C	4	0	50	F	1	0	1	Cl	1	0	0	I	3	0	0	
N	3	0	7	Si	4	0	0	Ni	2	0	0					

Error Margin (ppm):	100	DBE Range:	-2.0 - 1000.0	Electron Ions:	both
HC Ratio:	unlimited	Apply N Rule:	yes	Use MSn Info:	no
Max Isotopes:	all	Isotope RI (%):	1.00	Isotope Res:	10000
MSn Iso RI (%):	75.00	MSn Logic Mode:	AND	Max Results:	500

Event#: 2 MS(E-) Ret. Time : 0.653 -> 0.653 Scan# : 100 -> 100

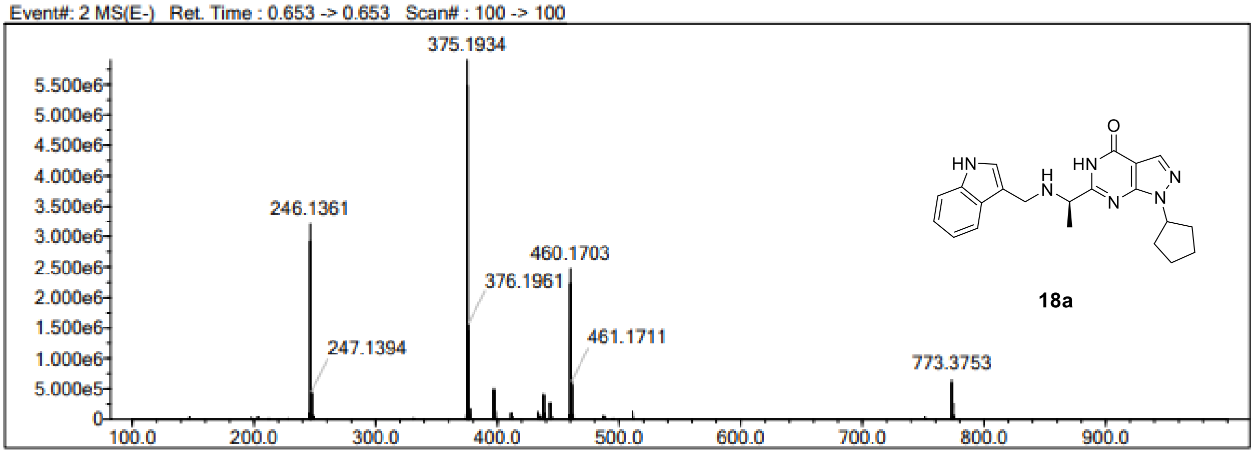

Measured region for 375.1934 m/z

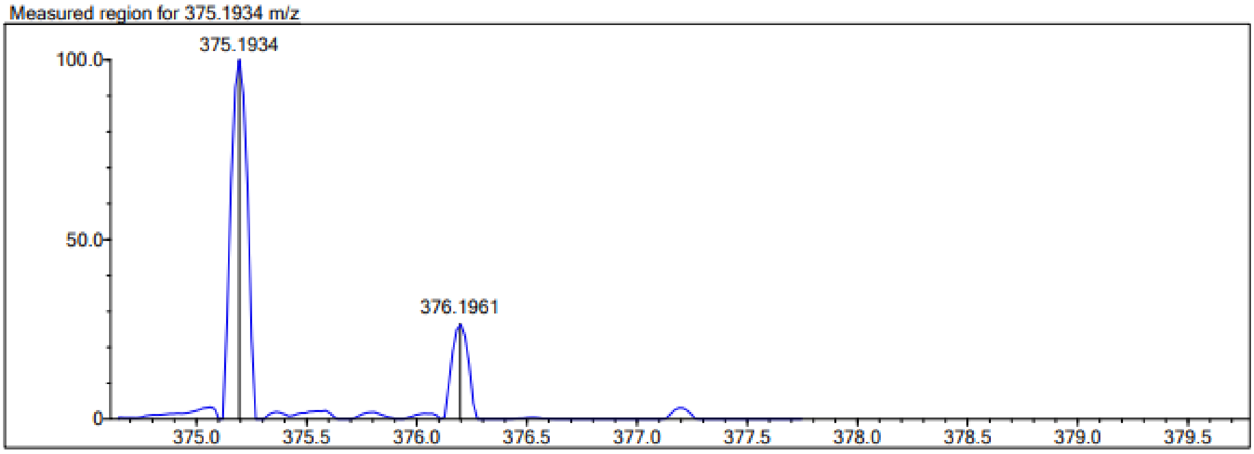

C21 H24 N6 O [M-H]- : Predicted region for 375.1939 m/z

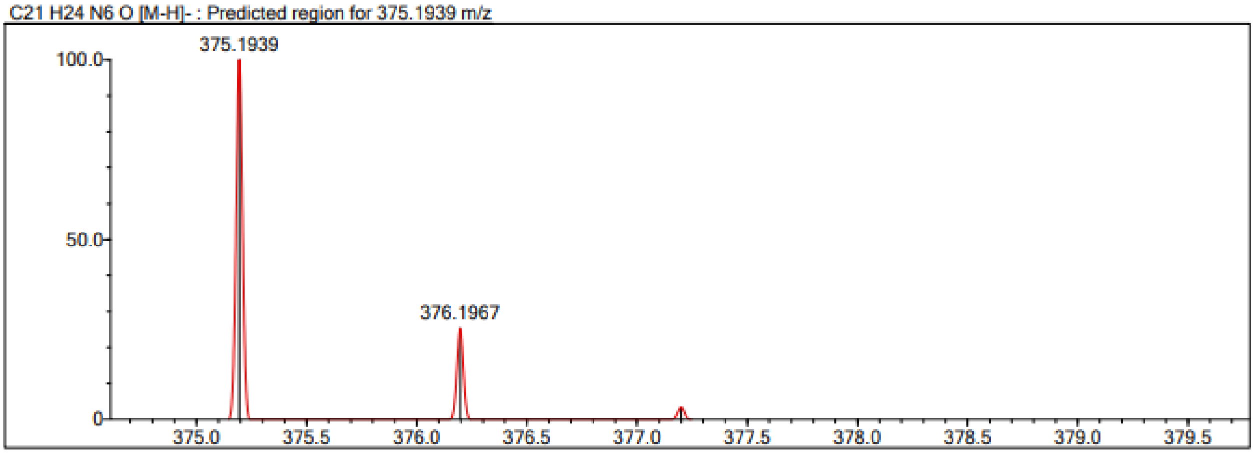

Rank	Score	Formula (M)	Ion	Meas. m/z	Pred. m/z	Df. (mDa)	Df. (ppm)	Iso	DBE
1	89.36	C21 H24 N6 O	[M-H]-	375.1934	375.1939	-0.5	-1.33	90.10	13.0

Elmt	Val.	Min	Max	Elmt	Val.	Min	Max	Elmt	Val.	Min	Max	Elmt	Val.	Min	Max	Use Adduct
H	1	0	50	O	2	0	13	P	3	0	0	Cu	2	0	0	H
B	3	0	0	18O	2	0	0	S	2	0	0	Br	1	0	2	
C	4	0	50	F	1	0	1	Cl	1	0	0	I	3	0	0	
N	3	0	7	Si	4	0	0	Ni	2	0	0					

Error Margin (ppm):	100	DBE Range:	-2.0 - 1000.0	Electron Ions:	both
HC Ratio:	unlimited	Apply N Rule:	yes	Use MSn Info:	no
Max Isotopes:	all	Isotope RI (%):	1.00	Isotope Res:	10000
MSn Iso RI (%):	75.00	MSn Logic Mode:	AND	Max Results:	500

Event#: 2 MS(E-) Ret. Time : 0.653 -> 0.653 Scan# : 100 -> 100

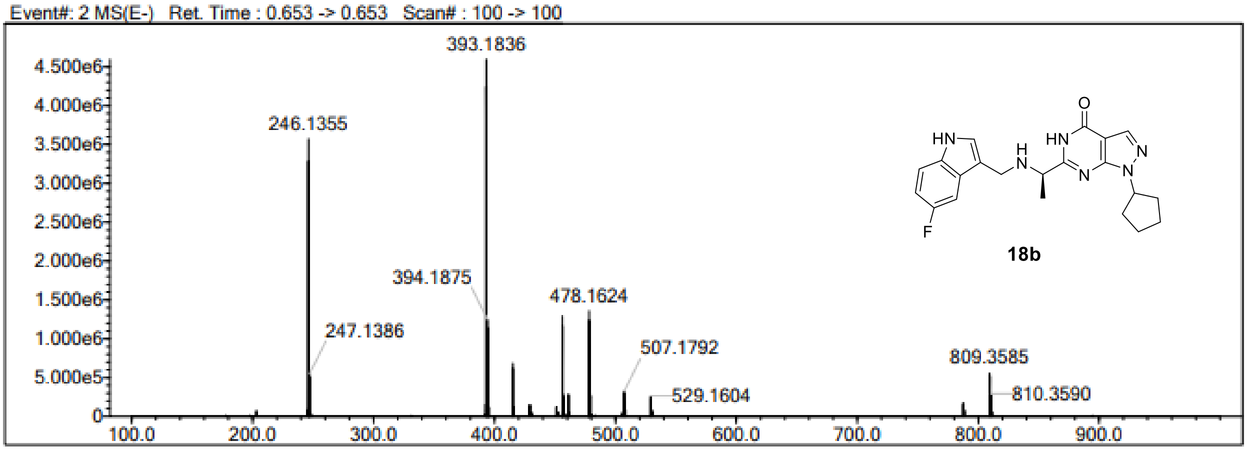

Measured region for 393.1836 m/z

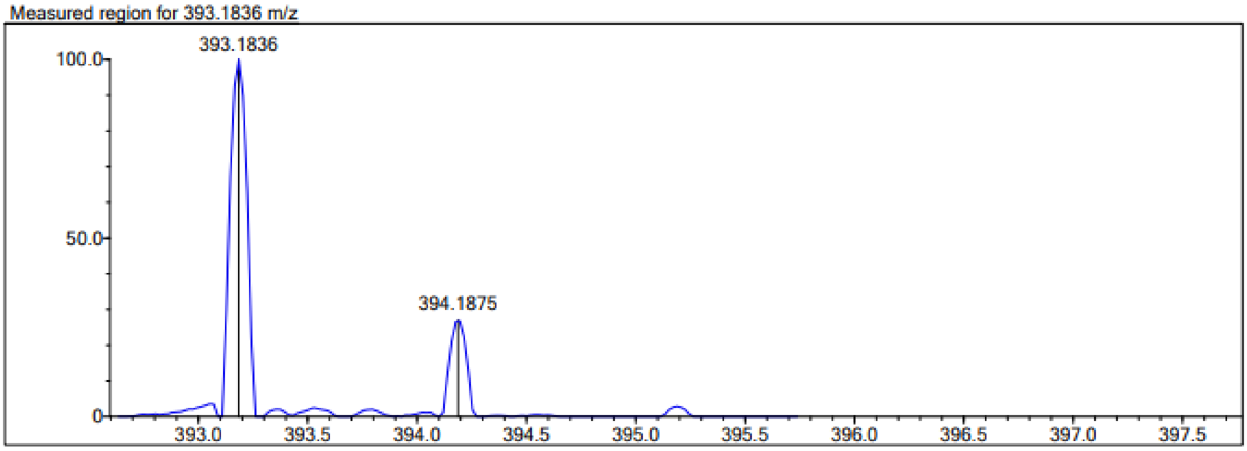

C21 H23 N6 O F [M-H]- : Predicted region for 393.1845 m/z

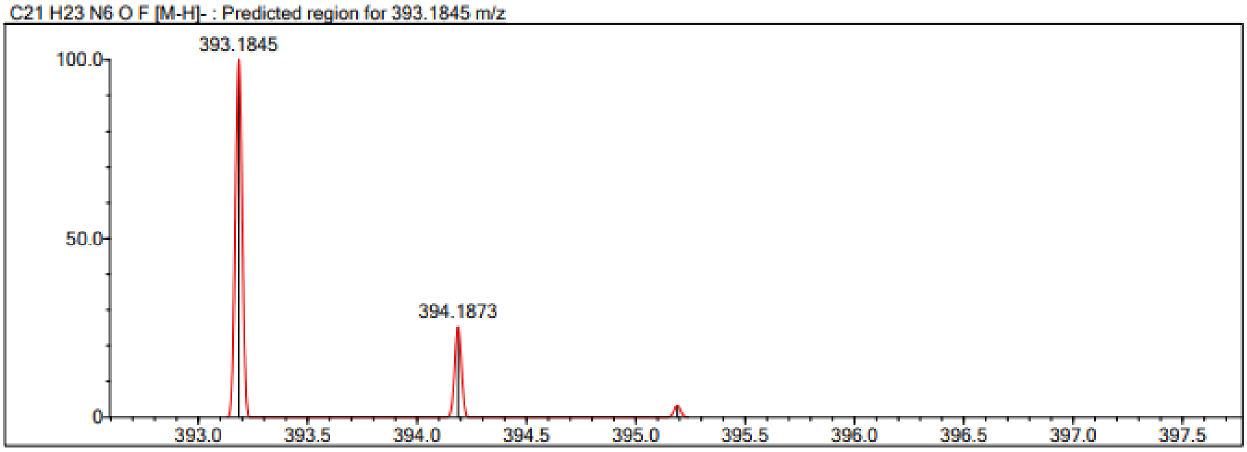

Rank	Score	Formula (M)	Ion	Meas. m/z	Pred. m/z	Df. (mDa)	Df. (ppm)	Iso	DBE
3	80.53	C21 H23 N6 O F	[M-H]-	393.1836	393.1845	-0.9	-2.29	83.21	13.0

Elmt	Val.	Min	Max	Elmt	Val.	Min	Max	Elmt	Val.	Min	Max	Elmt	Val.	Min	Max	Use Adduct
H	1	0	50	O	2	0	13	P	3	0	0	Cu	2	0	0	H
B	3	0	0	18O	2	0	0	S	2	0	0	Br	1	0	2	Na
C	4	0	50	F	1	0	0	Cl	1	0	0	I	3	0	0	
N	3	0	6	Si	4	0	0	Ni	2	0	0					

Error Margin (ppm): 100
HC Ratio: unlimited
Max Isotopes: all
MSn Iso RI (%): 75.00

DBE Range: -2.0 - 1000.0
Apply N Rule: yes
Isotope RI (%): 1.00
MSn Logic Mode: AND

Electron Ions: both
Use MSn Info: no
Isotope Res: 10000
Max Results: 500

Event#: 1 MS(E+) Ret. Time : 0.587 -> 0.587 Scan# : 89 -> 89

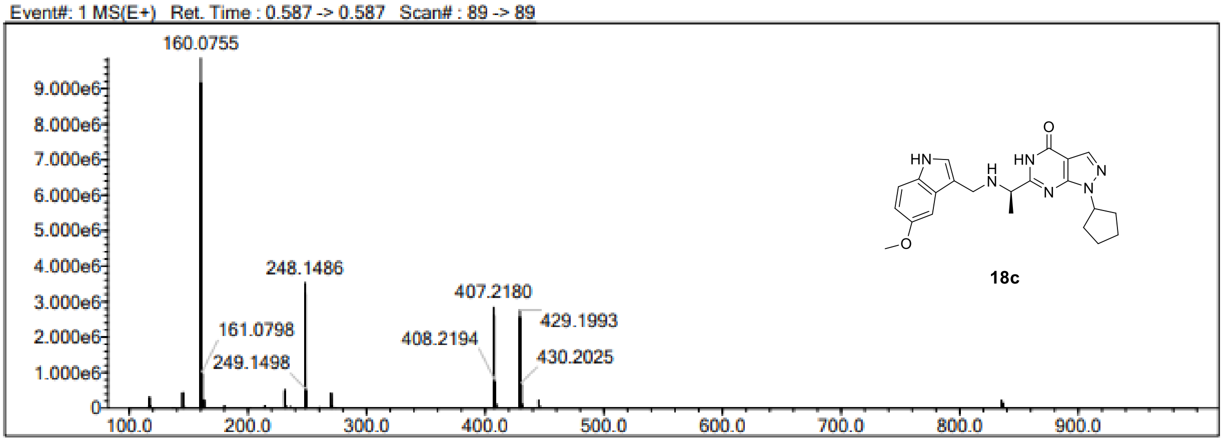

Measured region for 407.2180 m/z

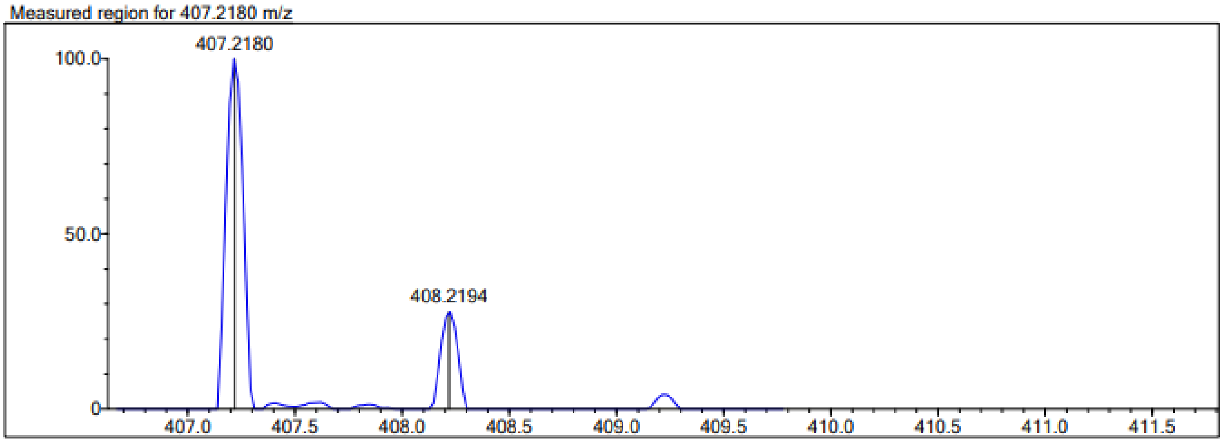

C22 H26 N6 O2 [M+H]+ : Predicted region for 407.2190 m/z

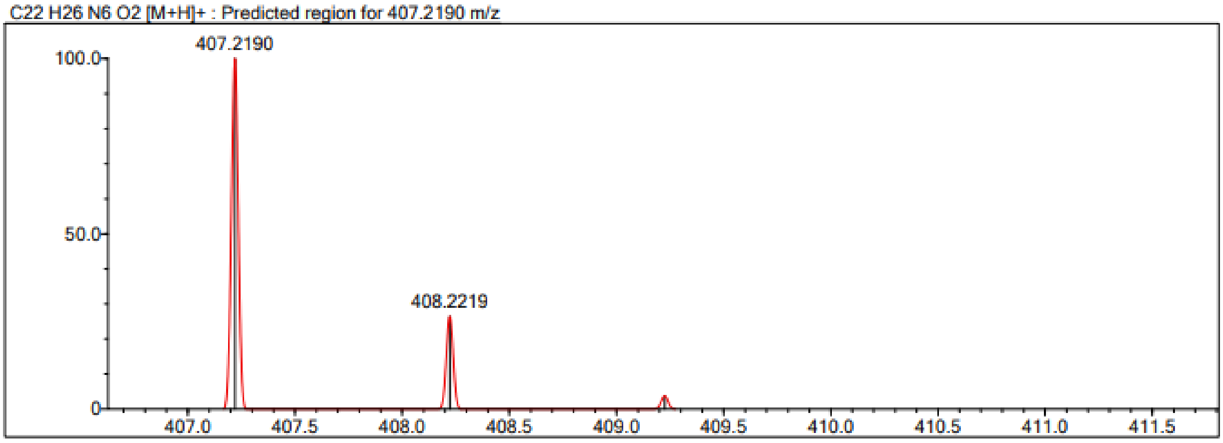

Rank	Score	Formula (M)	Ion	Meas. m/z	Pred. m/z	Df. (mDa)	Df. (ppm)	Iso	DBE
2	90.49	C22 H26 N6 O2	[M+H]+	407.2180	407.2190	-1.0	-2.46	93.92	13.0

Elmt	Val.	Min	Max	Elmt	Val.	Min	Max	Elmt	Val.	Min	Max	Elmt	Val.	Min	Max	Use Adduct
H	1	0	50	O	2	0	13	P	3	0	0	Cu	2	0	0	H
B	3	0	0	18O	2	0	0	S	2	0	0	Br	1	0	2	
C	4	0	50	F	1	0	1	Cl	1	0	0	I	3	0	0	
N	3	0	7	Si	4	0	0	Ni	2	0	0					

Error Margin (ppm): 100
HC Ratio: unlimited
Max Isotopes: all
MSn Iso RI (%): 75.00

DBE Range: -2.0 - 1000.0
Apply N Rule: yes
Isotope RI (%): 1.00
MSn Logic Mode: AND

Electron Ions: both
Use MSn Info: no
Isotope Res: 10000
Max Results: 500

Event#: 2 MS(E-) Ret. Time : 0.693 Scan# : 106

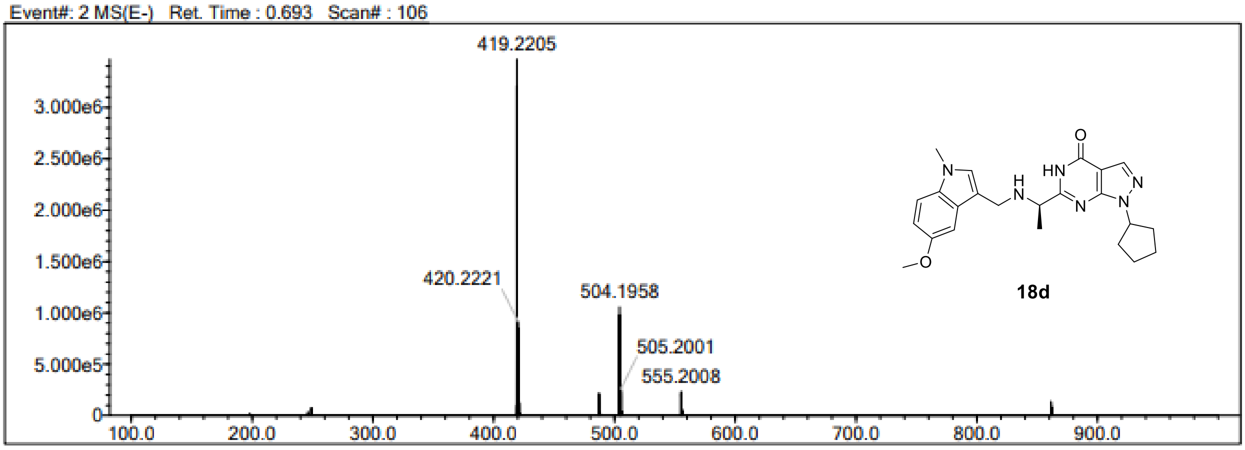

Measured region for 419.2205 m/z

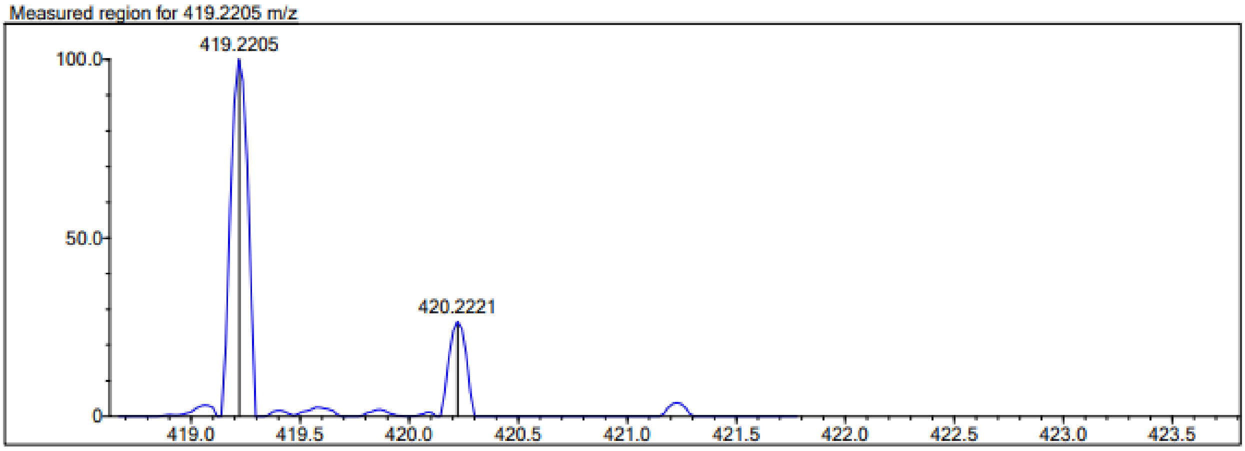

C23 H28 N6 O2 [M-H]- : Predicted region for 419.2201 m/z

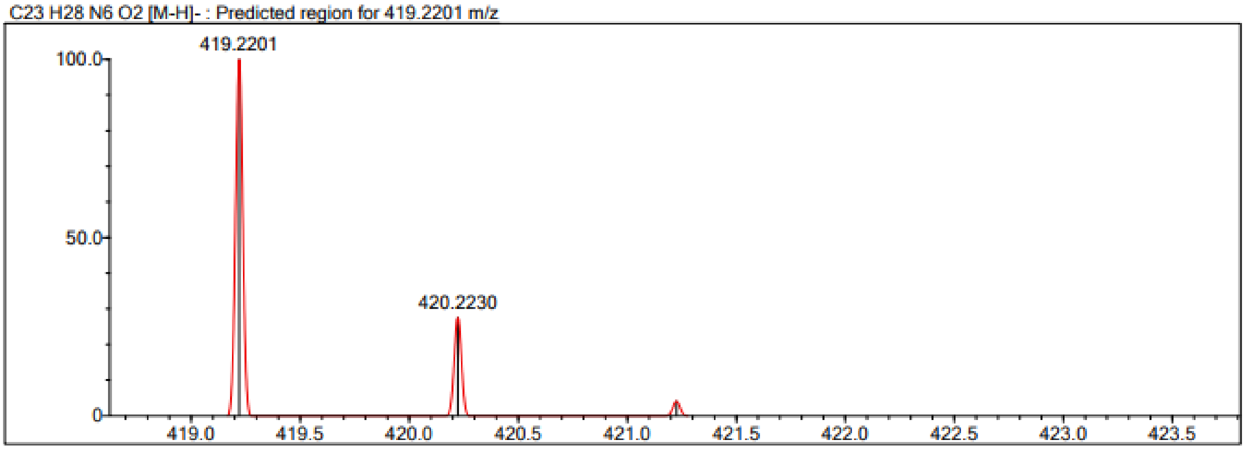

Rank	Score	Formula (M)	Ion	Meas. m/z	Pred. m/z	Df. (mDa)	Df. (ppm)	Iso	DBE
1	87.40	C23 H28 N6 O2	[M-H]-	419.2205	419.2201	0.4	0.95	87.40	13.0

Elmt	Val.	Min	Max	Elmt	Val.	Min	Max	Elmt	Val.	Min	Max	Elmt	Val.	Min	Max	Use Adduct
H	1	0	50	O	2	0	13	P	3	0	0	Cu	2	0	0	H
B	3	0	0	18O	2	0	0	S	2	0	0	Br	1	0	2	
C	4	0	50	F	1	0	1	Cl	1	0	0	I	3	0	0	
N	3	0	7	Si	4	0	0	Ni	2	0	0					

Error Margin (ppm):	100	DBE Range:	-2.0 - 1000.0	Electron Ions:	both
HC Ratio:	unlimited	Apply N Rule:	yes	Use MSn Info:	no
Max Isotopes:	all	Isotope RI (%):	1.00	Isotope Res:	10000
MSn Iso RI (%):	75.00	MSn Logic Mode:	AND	Max Results:	500

Event#: 2 MS(E-) Ret. Time : 0.960 Scan# : 146

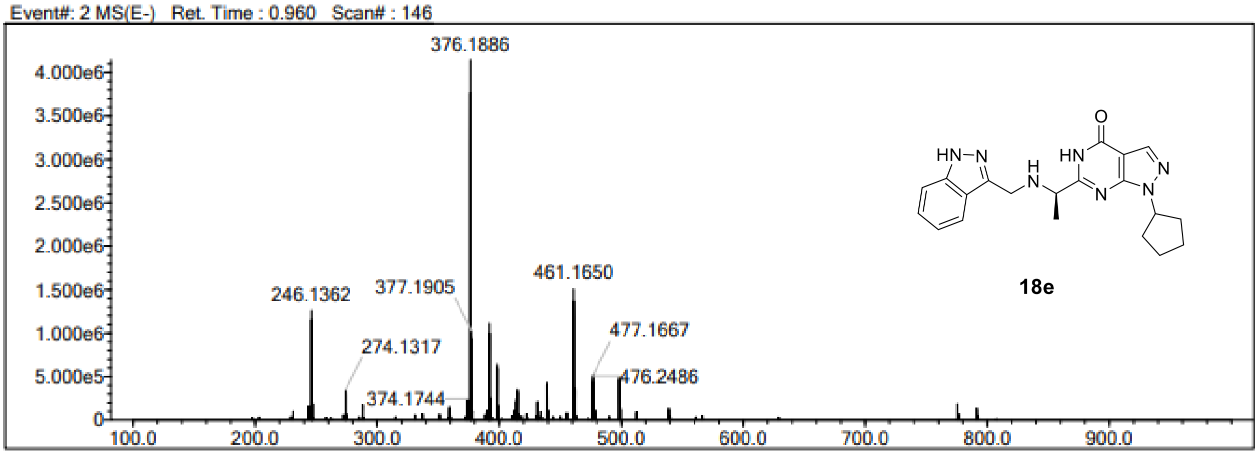

Measured region for 376.1886 m/z

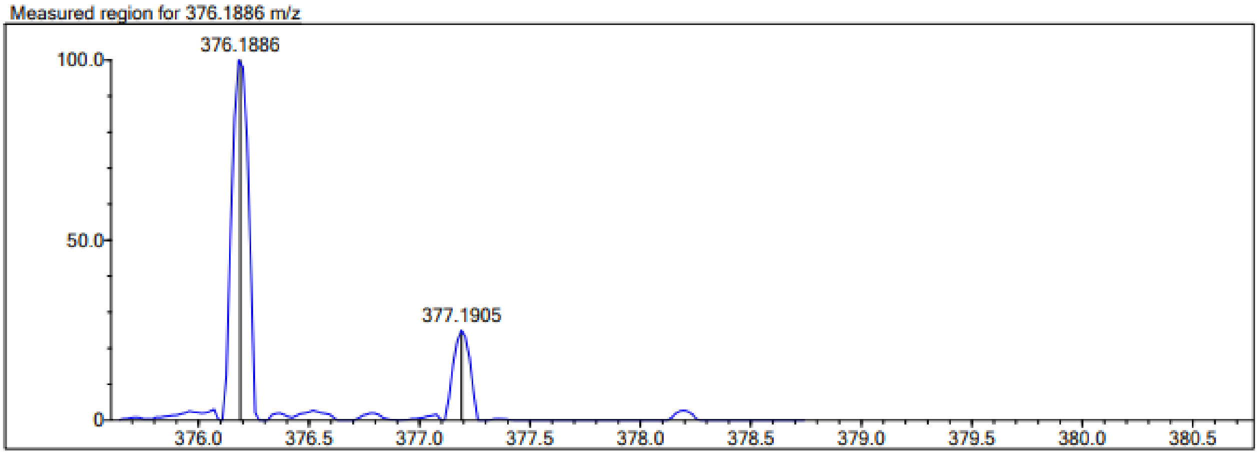

C20 H23 N7 O [M-H]- : Predicted region for 376.1891 m/z

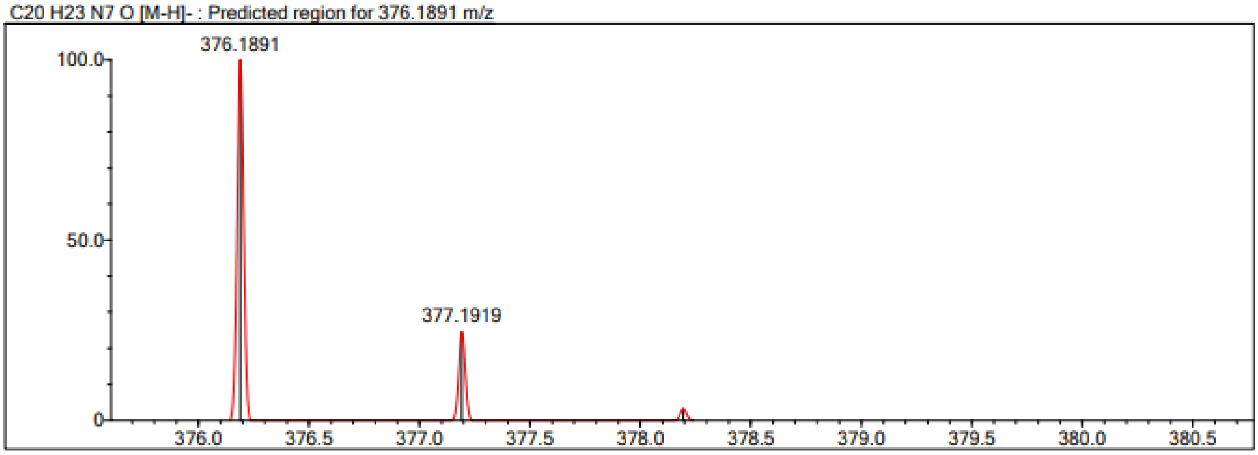

Rank	Score	Formula (M)	Ion	Meas. m/z	Pred. m/z	Df. (mDa)	Df. (ppm)	Iso	DBE
1	86.96	C20 H23 N7 O	[M-H]-	376.1886	376.1891	-0.5	-1.33	87.68	13.0

Elmt	Val.	Min	Max	Elmt	Val.	Min	Max	Elmt	Val.	Min	Max	Elmt	Val.	Min	Max	Use Adduct
H	1	0	50	O	2	0	13	P	3	0	0	Cu	2	0	0	H
B	3	0	0	18O	2	0	0	S	2	0	0	Br	1	0	2	
C	4	0	50	F	1	0	1	Cl	1	0	0	I	3	0	0	
N	3	0	7	Si	4	0	0	Ni	2	0	0					

Error Margin (ppm):	100	DBE Range:	-2.0 - 1000.0	Electron Ions:	both
HC Ratio:	unlimited	Apply N Rule:	yes	Use MSn Info:	no
Max Isotopes:	all	Isotope RI (%):	1.00	Isotope Res:	10000
MSn Iso RI (%):	75.00	MSn Logic Mode:	AND	Max Results:	500

Event#: 2 MS(E-) Ret. Time : 0.867 Scan# : 132

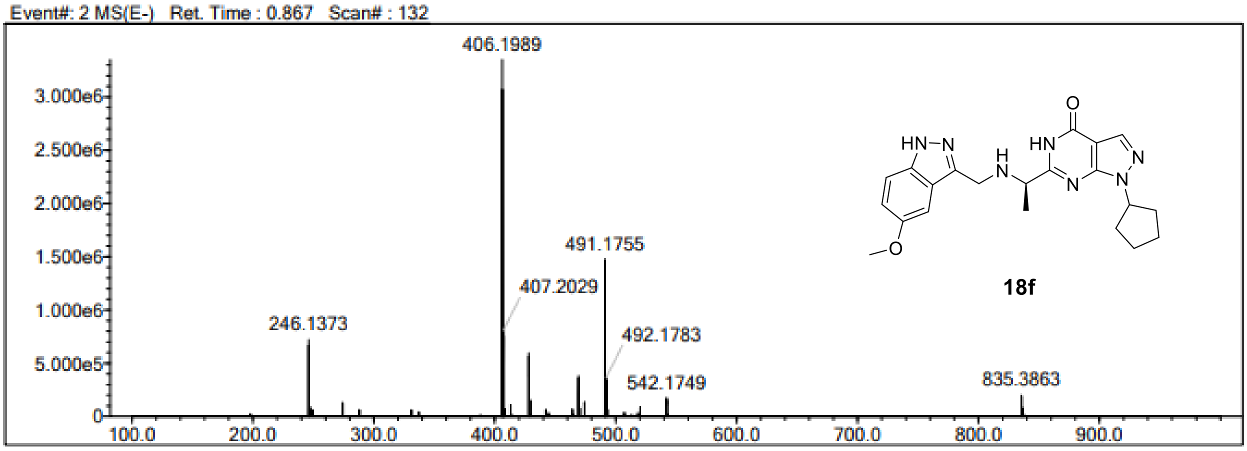

Measured region for 406.1989 m/z

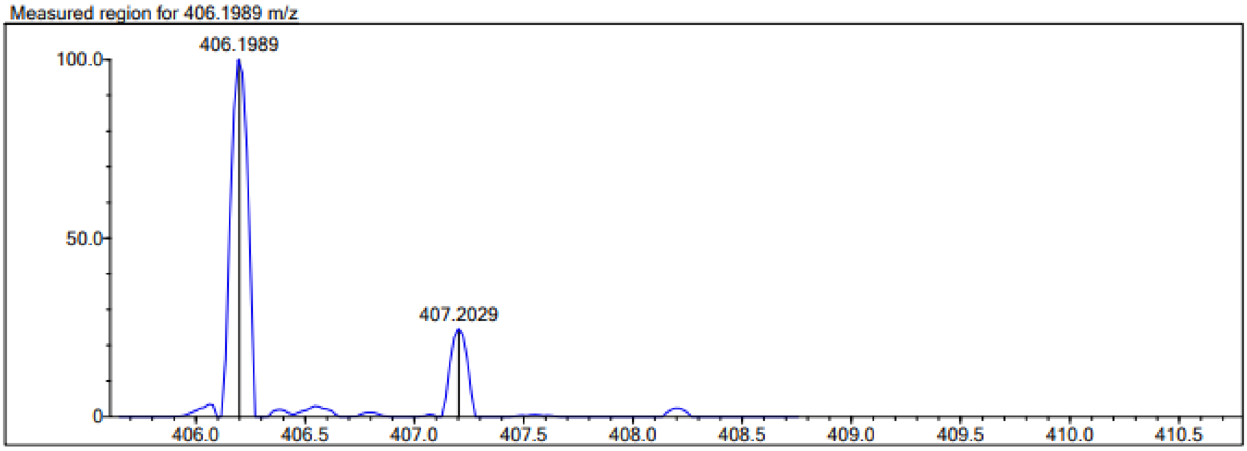

C21 H25 N7 O2 [M-H]- : Predicted region for 406.1997 m/z

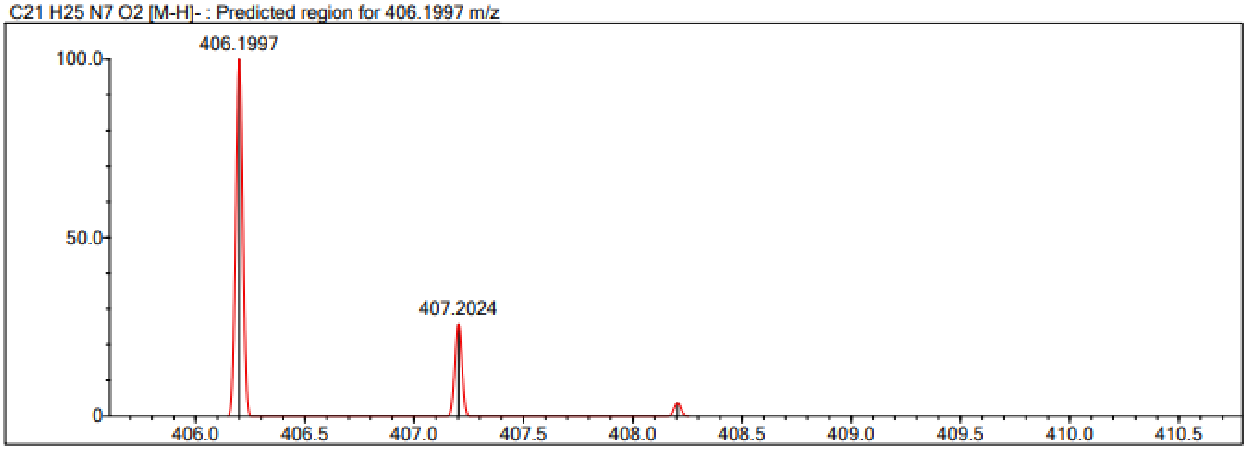

Rank	Score	Formula (M)	Ion	Meas. m/z	Pred. m/z	Df. (mDa)	Df. (ppm)	Iso	DBE
2	87.25	C21 H25 N7 O2	[M-H]-	406.1989	406.1997	-0.8	-1.97	89.42	13.0

3. Molecular dynamics simulations

MD simulations were subsequently performed to more accurately predict the binding patterns of the molecules. The software AMBER 16.0 [1] was used to simulate the interactions between the molecules and PDE9. Partial atomic charges for the docking poses of the molecules were calculated using the Hartree–Fock method at the 6-31G* level with Gaussian 03 [2]. The Antechamber program was then employed to fit the restricted electrostatic potential (RESP) and assign parameters from the general AMBER force field (GAFF). The amber03 force field was used for the protein, while force field parameters for Zn^{2+} and Mg^{2+} were assigned using the 'nonbond model' method [3]. The oxygen atom bridging the two metal ions was treated as a hydroxide ion. An 8 Å TIP3P water box in the shape of a truncated octahedron, along with Na^{+} ions, was added to neutralize the system.

The well-prepared ligands and protein were subjected to MD simulations. An 8 ns MD simulation was performed in the *NPT* ensemble, maintaining a constant pressure of 1 atm and a temperature of 300 K. Periodic boundary conditions were applied, with an 8 Å cutoff for long-range electrostatic interactions using the particle mesh Ewald (PME) method [4]. The SHAKE algorithm [5] was used to constrain all bonds involving hydrogen atoms, allowing a time step of 2 fs. To accelerate the simulations, an Intel Core i7-8700K and an NVIDIA GeForce RTX 2080 GPU were utilized, leveraging GPU capabilities for efficient floating-point calculations. From the final 1 ns of the trajectory, 100 snapshots were extracted for MM-GBSA binding free energy calculations [6], which were conducted with default parameter settings. The entropy contribution of the PDE5-ligand complexes was omitted to expedite the calculations.

In light of the MM-GBSA method, the binding free energy (ΔG_{bind}) can be calculated by the following Eq. 1, where the free energies of complex, receptor and ligand are represented by G_{comp}, G_{rec} and G_{lig}, respectively.

$$\Delta G_{\text{bind}} = G_{\text{comp}} - G_{\text{rec}} - G_{\text{lig}} \qquad (1)$$

The binding free energy of each system was evaluated as the sum of the MM energy (E_{MM}), the solvation free energy (G_{solv}), and the entropy contribution (S), respectively, as described in Eq. 2.

$$\Delta G_{\text{bind}} = \Delta E_{\text{MM}} + \Delta G_{\text{solv}} - T\Delta S \qquad (2)$$

ΔE_{MM} is the gas phase interaction energy, which can be decomposed into $E_{\text{MM,comp}}$, $E_{\text{MM,rec}}$ and $E_{\text{MM,lig}}$. Solvation free energy is evaluated by the sum of the electrostatic solvation free energy (ΔG_{PB}) and nonpolar solvation free energy (ΔG_{np}), resulting in Eq. 3.

$$\Delta G_{\text{solv}} = \Delta G_{\text{PB}} + \Delta G_{\text{np}} \qquad (3)$$

ΔG_{PB} was calculated by the Poisson Boltzmann (PB) Eq, whereas ΔG_{np} was calculated according to Eq. 4. The default parameters were adopted, with $\gamma = 0.0072$ kcal/(Å^2) and $b = 0$ kcal/mol.

$$\Delta G_{\text{np}} = \gamma\ \text{SASA} + b \qquad (4)$$

For a compromise between efficiency and accuracy, the entropy contribution term ($-T\Delta S$) was omitted for ΔG_{bind} in Eq. 2, since the calculations of the entropy contribution are extremely time-consuming for large protein-ligand systems.

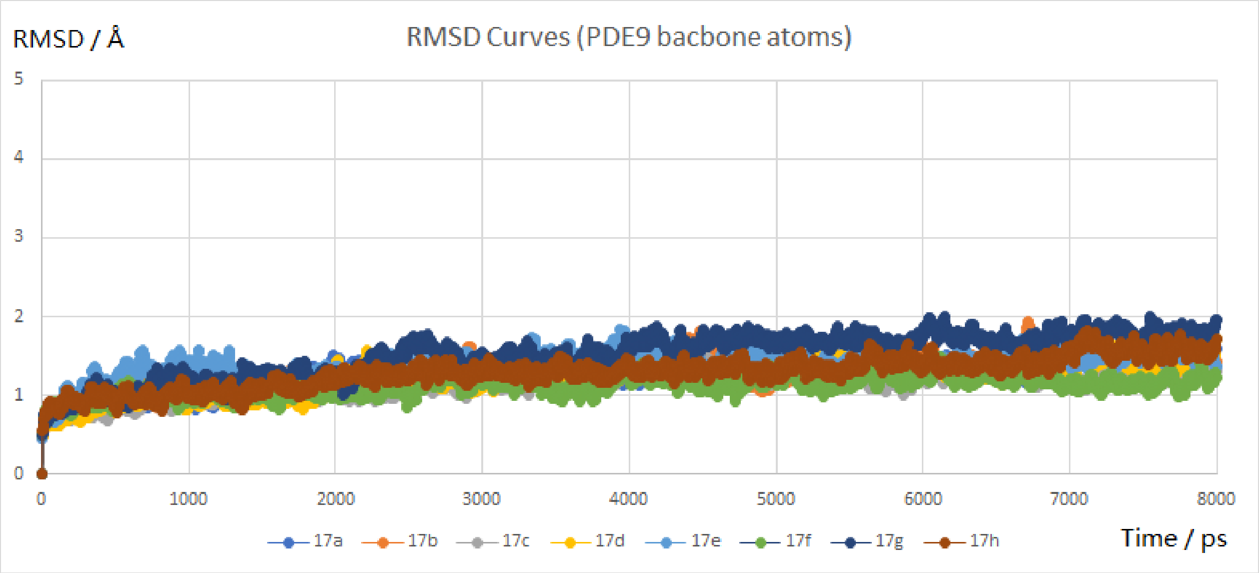

Figure S1. RMSD curves of backbone atoms for PDE9-inhibitor complexes.

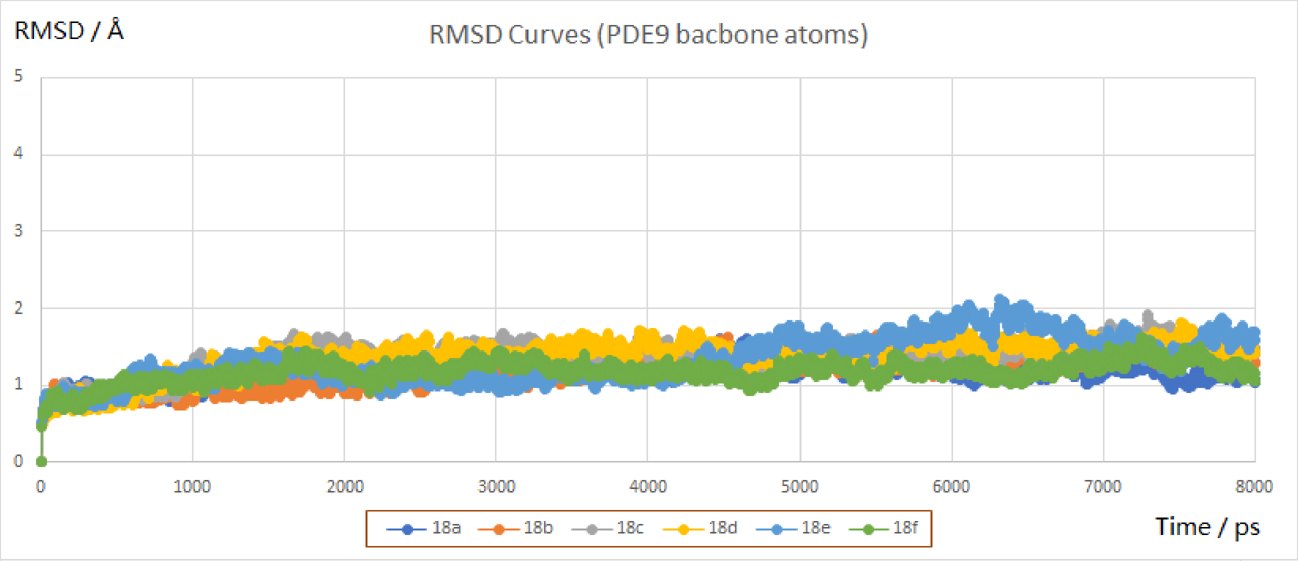

Figure S2. RMSD curves of backbone atoms for PDE9-inhibitor complexes.

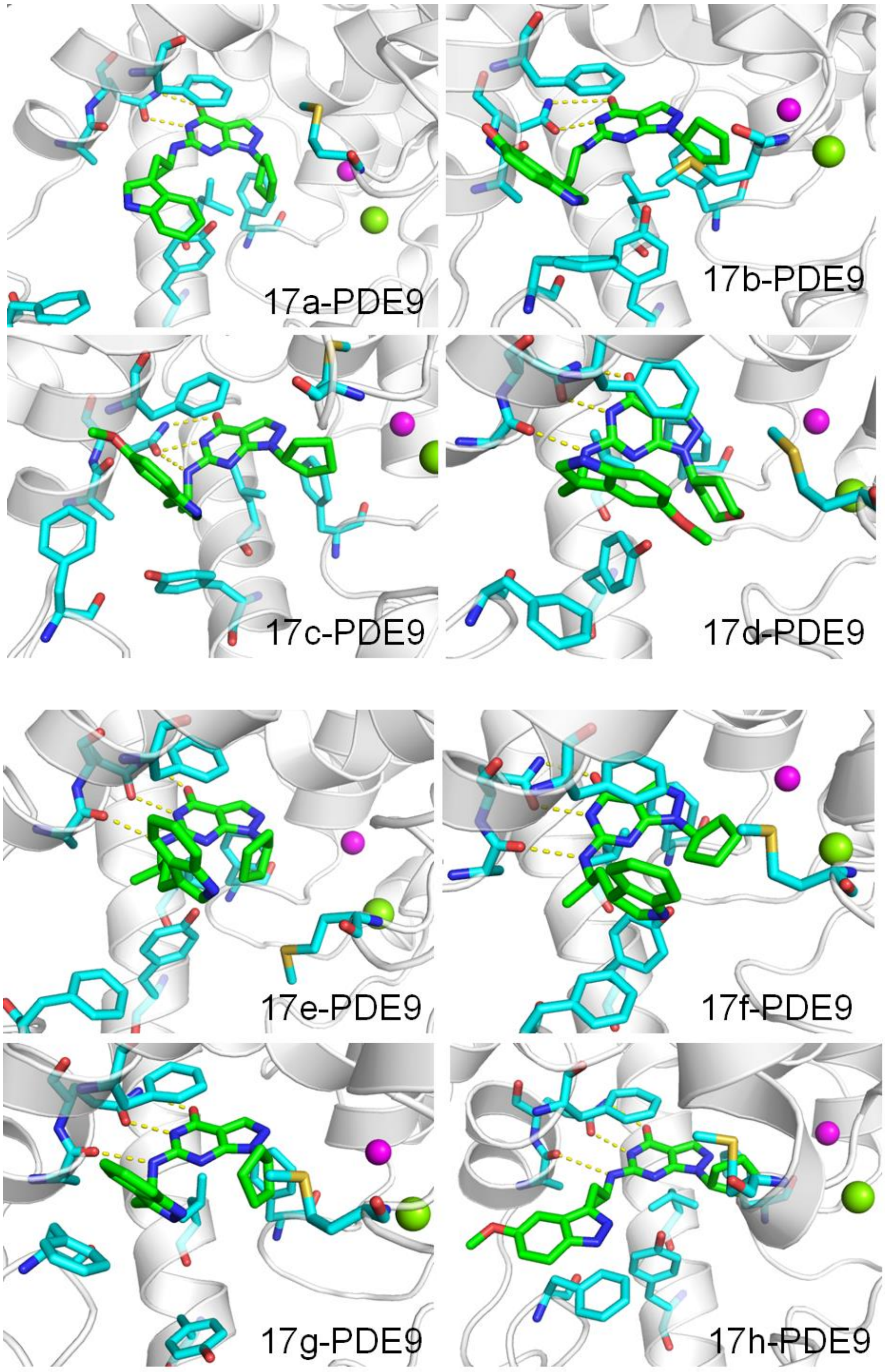

Figure S3. Binding patterns of each PDE9-inhibitor complex predicted by molecular dynamics simulations.

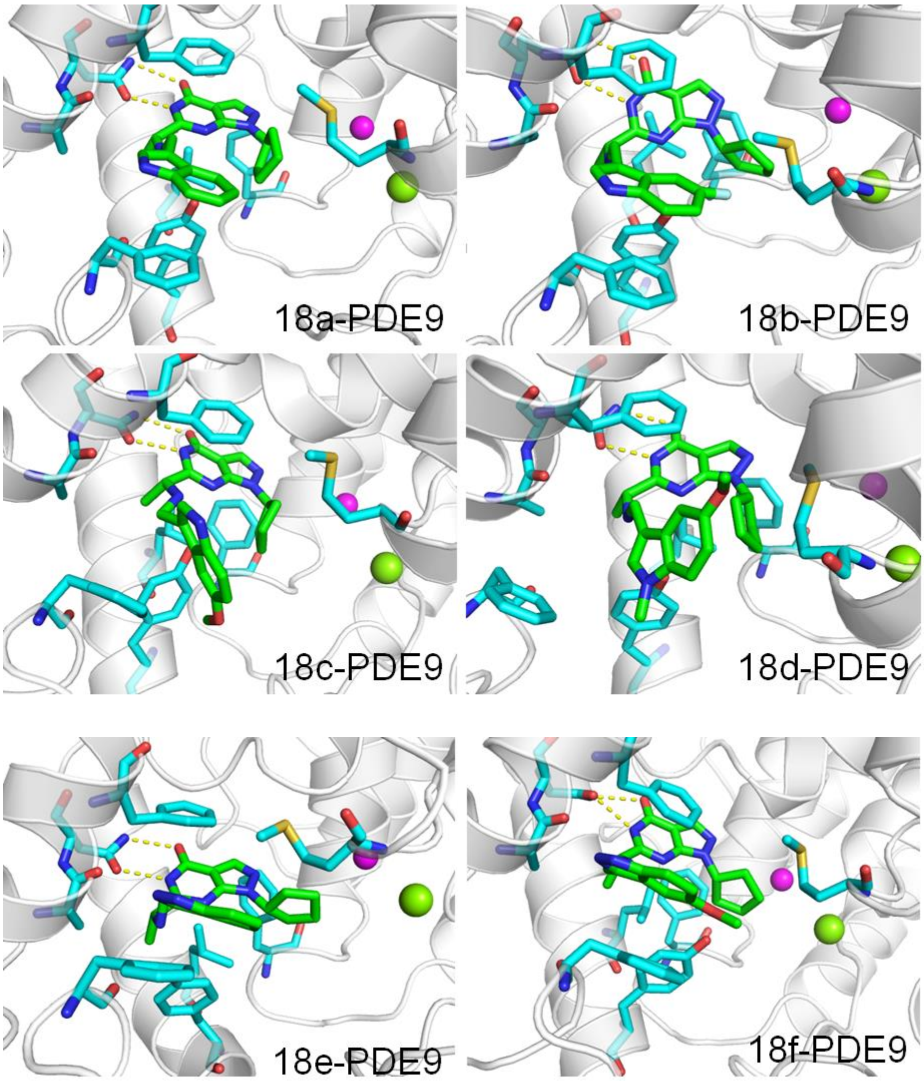

Figure S4. Binding patterns of each PDE9-inhibitor complex predicted by molecular dynamics simulations.

4. Enzymatic assays against PDE9

The assay buffer contains 50 mM Tris-HCl (pH 8.0), 10 mM $MgCl_2$ or 4 mM $MnCl_2$, 1 mM DTT, and 10-20 nM ^{3}H-cGMP (15000−30000 cpm/assay, $CPM_{control}$). The reaction was carried out at room temperature for 15 min and then terminated by addition of 0.2 M ZnSO4. The reaction product ^{3}H-GMP was precipitated out by 0.2 N $BaSO_4$, whereas unreacted ^{3}H-cGMP remained in the supernatant. The radioactivity in the supernatant was measured in 2.5 mL of Ultima Gold liquid scintillation cocktails (PerkinElmer) by a PerkinElmer 2910 liquid scintillation counter (CPM_{enzyme}). For the measurement of IC_{50} of inhibitors, at least seven concentrations of inhibitors were used in the presence of ^{3}H-cGMP. The enzyme concentration in a range of 10− 100 ng/mL, which hydrolyzed 50- 70% of the substrate, was chosen for each inhibitory assay. The radioactivity was recorded as $CPM_{inhibitor}$. The IC_{50} values for PDE9 were calculated using a nonlinear regression analysis.

$$Inhibition = \left(1 - \frac{1 - \frac{CPM_{inhibitor}}{CPM_{control}}}{1 - \frac{CPM_{enzyme}}{CPM_{control}}}\right) \times 100\%$$

Compound **17b**

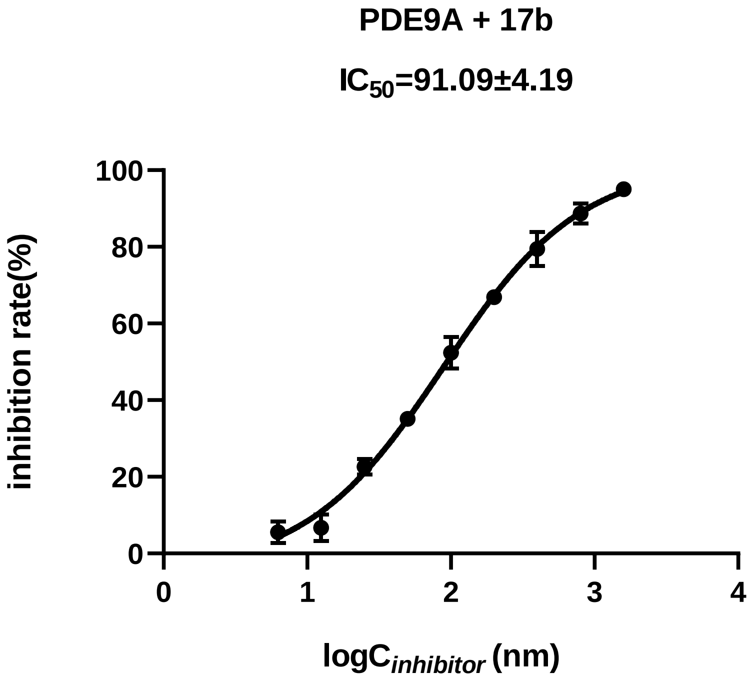

Concentration (nM)	$CPM_{inhibitor}$	CPM_{enzyme}	$CPM_{control}$	$\log c_{inhibitor}$	Inhibition
1600	18488	8760.2	19006.2	3.20412	94.94%
800	17656	8760.2	19006.2	2.90309	86.82%
400	16579	8760.2	19006.2	2.60206	76.31%
200	15529.2	8760.2	19006.2	2.30103	66.06%
100	13824.2	8760.2	19006.2	2	49.42%
50	12248.2	8760.2	19006.2	1.69897	34.04%
25	10933	8760.2	19006.2	1.39794	21.21%
12.5	9193	8760.2	19006.2	1.09691	4.22%
6.25	9383	8760.2	19006.2	0.79588	6.08%

Concentration (nM)	$CPM_{inhibitor}$	CPM_{enzyme}	$CPM_{control}$	$\log c_{inhibitor}$	Inhibition
1600	18508	8760.2	19006.2	3.20412	95.14%
800	18039	8760.2	19006.2	2.90309	90.56%
400	17222	8760.2	19006.2	2.60206	82.59%
200	15694	8760.2	19006.2	2.30103	67.67%
100	14425.4	8760.2	19006.2	2	55.29%
50	12476	8760.2	19006.2	1.69897	36.27%
25	11231	8760.2	19006.2	1.39794	24.11%
12.5	9706.2	8760.2	19006.2	1.09691	9.23%
6.25	9699.2	8760.2	19006.2	0.79588	9.16%

Compound **17d**

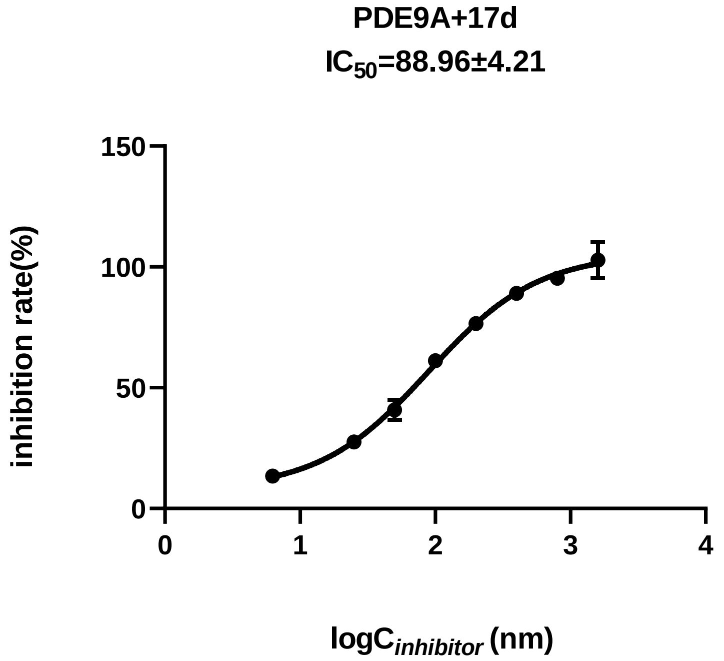

Concentration (nM)	$CPM_{inhibitor}$	CPM_{enzyme}	$CPM_{control}$	$\log c_{inhibitor}$	Inhibition
1600	18173.6	8760.2	18411.2	3.20412	97.54%
800	17861	8760.2	18411.2	2.90309	94.30%
400	17221	8760.2	18411.2	2.60206	87.67%
200	16060.2	8760.2	18411.2	2.30103	75.64%
100	14638.4	8760.2	18411.2	2	60.91%
50	12407.2	8760.2	18411.2	1.69897	37.79%
25	11537	8760.2	18411.2	1.39794	28.77%
12.5	11338	8760.2	18411.2	1.09691	26.71%
6.25	10087.2	8760.2	18411.2	0.79588	13.75%

Concentration (nM)	$CPM_{inhibitor}$	CPM_{enzyme}	$CPM_{control}$	$\log c_{inhibitor}$	Inhibition
1600	19190	8760.2	18411.2	3.20412	108.07%
800	18067	8760.2	18411.2	2.90309	96.43%
400	17499	8760.2	18411.2	2.60206	90.55%
200	16249	8760.2	18411.2	2.30103	77.60%
100	14702	8760.2	18411.2	2	61.57%
50	12982.2	8760.2	18411.2	1.69897	43.75%
25	11312.6	8760.2	18411.2	1.39794	26.45%
12.5	12106	8760.2	18411.2	1.09691	34.67%
6.25	10015.2	8760.2	18411.2	0.79588	13.00%

Compound **18c**

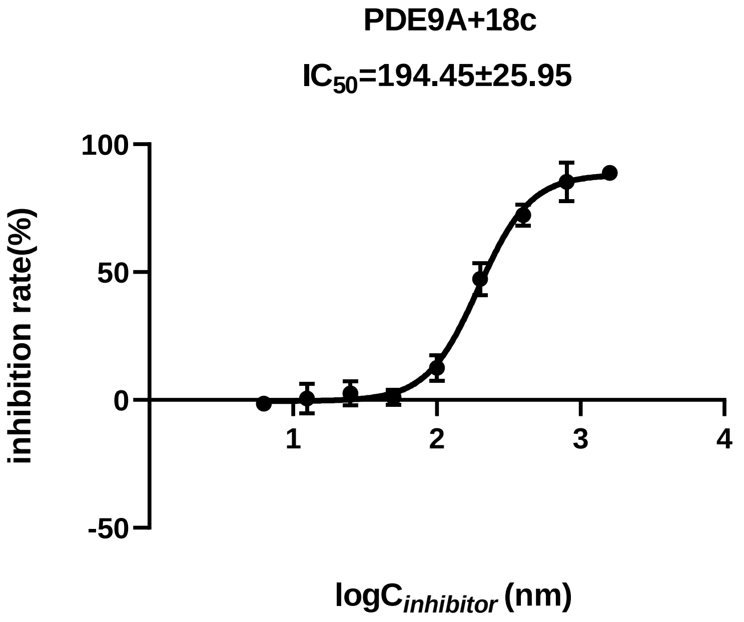

Concentration (nM)	$CPM_{inhibitor}$	CPM_{enzyme}	$CPM_{control}$	$\log c_{inhibitor}$	Inhibition
1600	17958.4	7609.2	19458	3.20412	87.34%
800	17091	7609.2	19458	2.90309	80.02%
400	15830	7609.2	19458	2.60206	69.38%
200	13739	7609.2	19458	2.30103	51.73%
100	9521	7609.2	19458	2	16.13%
50	7983	7609.2	19458	1.69897	3.15%
25	8314	7609.2	19458	1.39794	5.95%
12.5	7179	7609.2	19458	1.09691	-3.63%
6.25	7513	7609.2	19458	0.79588	-0.81%

Concentration (nM)	$CPM_{inhibitor}$	CPM_{enzyme}	$CPM_{control}$	$\log c_{inhibitor}$	Inhibition
1600	18300	7609.2	19458	3.20412	90.23%
800	18346.2	7609.2	19458	2.90309	90.62%
400	16533	7609.2	19458	2.60206	75.31%
200	12692	7609.2	19458	2.30103	42.90%
100	8681.2	7609.2	19458	2	9.05%
50	7475	7609.2	19458	1.69897	-1.13%
25	7512	7609.2	19458	1.39794	-0.82%
12.5	8158	7609.2	19458	1.09691	4.63%
6.25	7373.2	7609.2	19458	0.79588	-1.99%

Compound **18d**

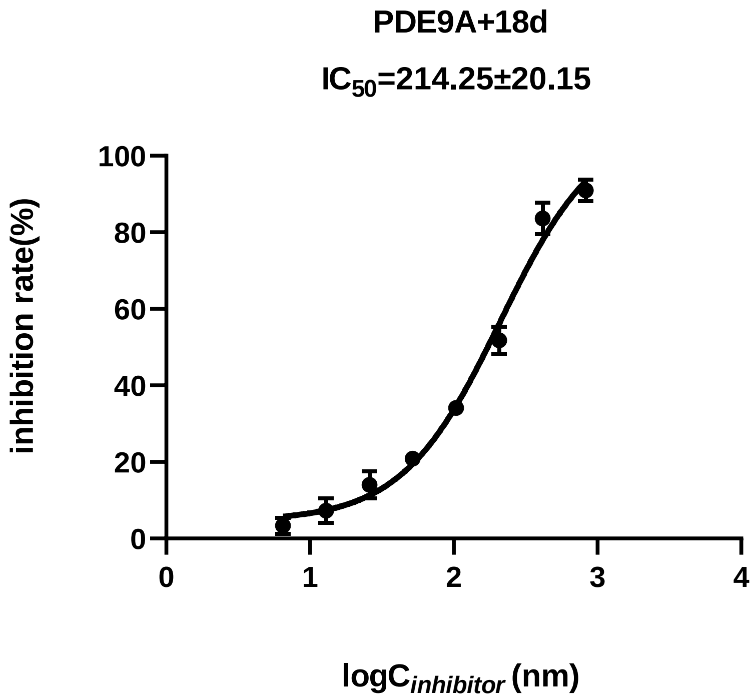

Concentration (nM)	$CPM_{inhibitor}$	CPM_{enzyme}	$CPM_{control}$	$\log c_{inhibitor}$	Inhibition
830.4	17815.2	7466	19100	2.919287	88.96%
415.2	16862.2	7466	19100	2.618257	80.76%
207.6	13204	7466	19100	2.317227	49.32%
103.8	11516	7466	19100	2.016197	34.81%
51.9	9984	7466	19100	1.715167	21.64%
25.95	8808	7466	19100	1.414137	11.54%
12.975	8583	7466	19100	1.113107	9.60%
6.4875	7677	7466	19100	0.812077	1.81%

Concentration (nM)	$CPM_{inhibitor}$	CPM_{enzyme}	$CPM_{control}$	$\log c_{inhibitor}$	Inhibition
830.4	18280	7466	19100	2.919287	92.95%
415.2	17535	7466	19100	2.618257	86.55%
207.6	13780.4	7466	19100	2.317227	54.28%
103.8	11352	7466	19100	2.016197	33.40%
51.9	9814	7466	19100	1.715167	20.18%
25.95	9396.2	7466	19100	1.414137	16.59%
12.975	8047	7466	19100	1.113107	4.99%
6.4875	8031	7466	19100	0.812077	4.86%

5. HPLC spectrums for the purity of representative target compounds

Compound 17b: Hypersil BDS C18 (0.46 × 15 cm); UV detection at 254 nm; elution, MeOH/H2O = 95/5; T = 25℃ ; flow rate = 1.0 mL/min. Purity = 96.62%.

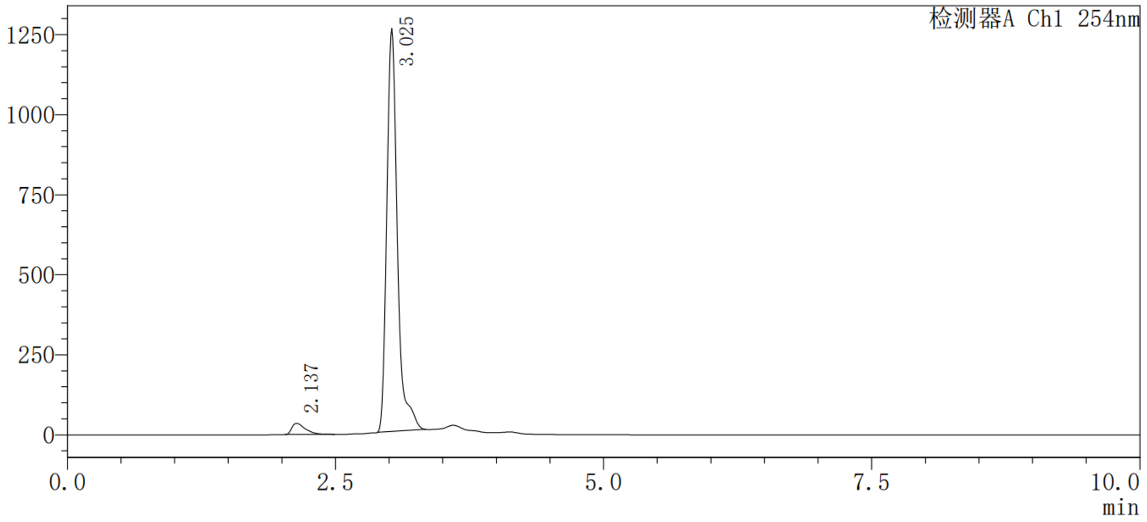

Peak	Retention time	Peak area	Peak height	Peak area %
1	2.137	301963	34805	3.38%
2	3.025	8631082	1258807	96.62%
In total		8933045	1293612	100%

Compound 17d: Hypersil BDS C18 (0.46 × 15 cm); UV detection at 254 nm; elution, MeOH/H2O = 95/5; T = 25℃; flow rate = 1.0 mL/min. Purity =99.91%.

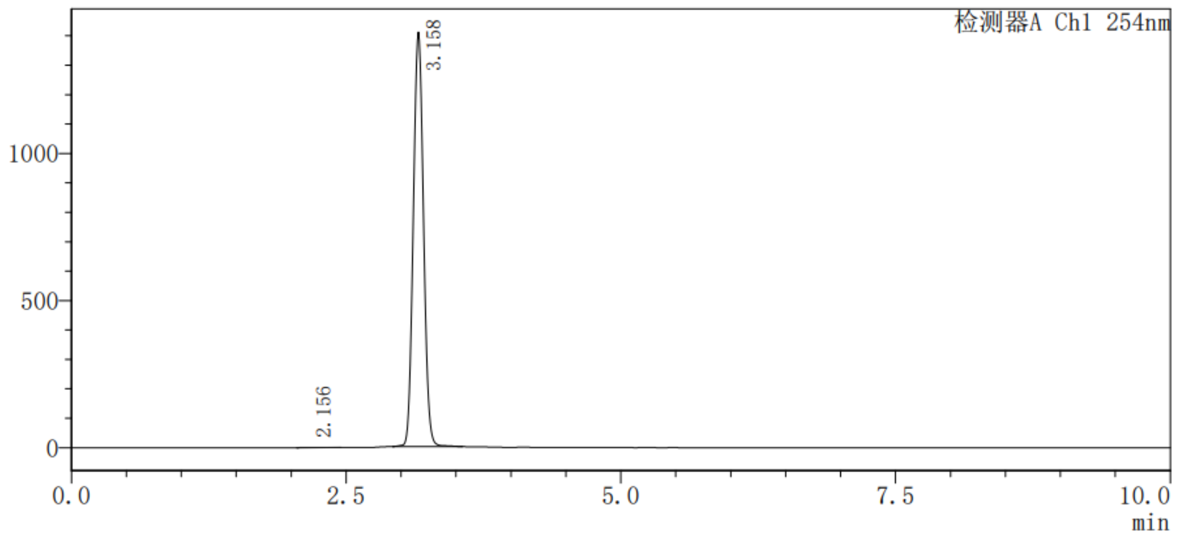

Peak	Retention time	Peak area	Peak height	Peak area %
1	2.156	7867	878	0.09%
2	3.158	9120524	1408776	99.91%
In total		9128391	1409654	100%

Compound 18b: Hypersil BDS C18 (0.46 × 15 cm); UV detection at 254 nm; elution, MeOH/H2O = 90/10; T = 25℃; flow rate = 1.5 mL/min. Purity = 96.04%.

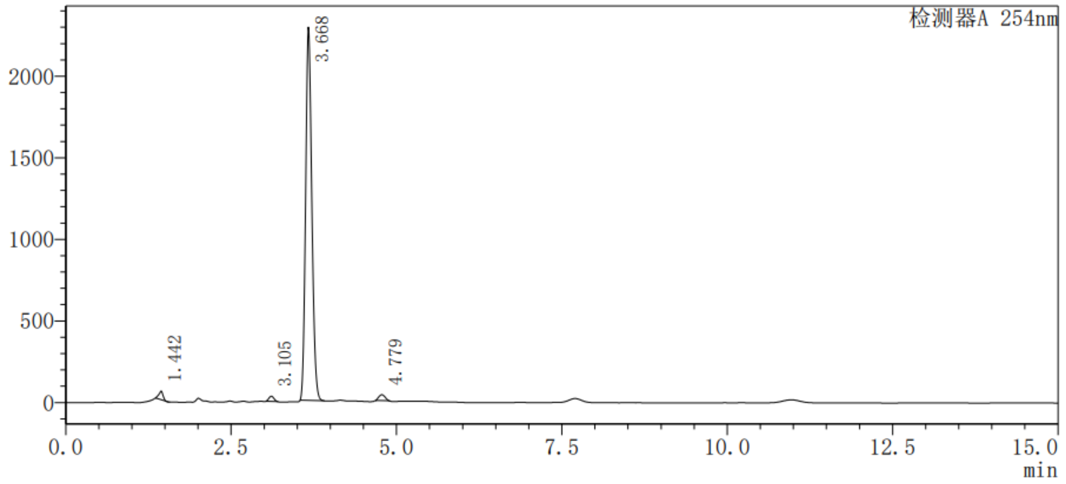

Peak	Retention time	Peak area	Peak height	Peak area %
1	1.442	215413	52379	1.393%
2	3.105	161282	31505	1.043%
3	3.668	14852243	2288715	96.042%
4	4.779	235424	35745	1.522%
In total		15464363	2408344	100%

Compound 18d: Hypersil BDS C18 (0.46 × 15 cm); UV detection at 254 nm; elution, MeOH/H2O = 90/10; T = 25℃; flow rate = 1.7 mL/min. Purity = 91.22%.

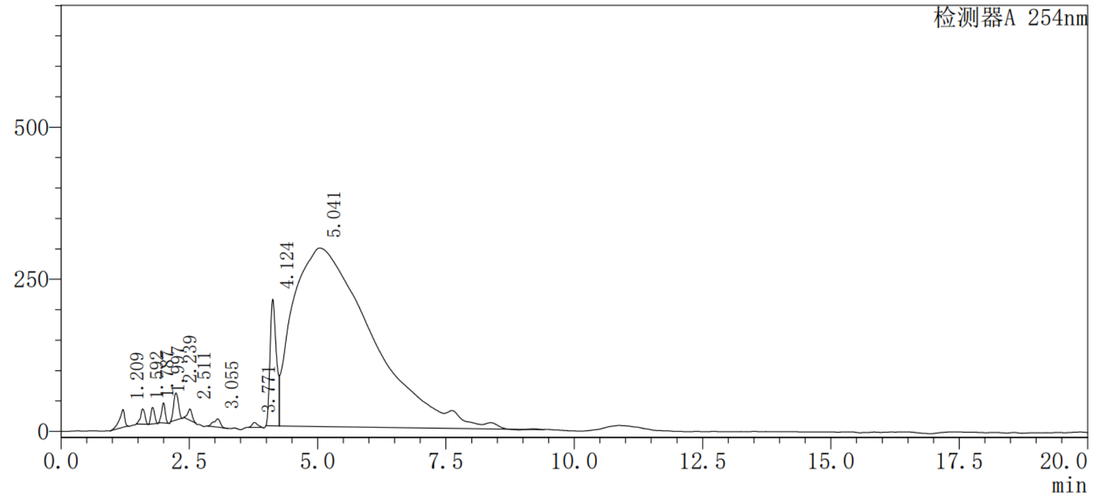

Peak	Retention time	Peak area	Peak height	Peak area %
1	1.209	211969	29237	0.612%
2	1.592	150838	25341	0.435%
3	1.787	136113	27095	0.393%
4	1.997	154155	32933	0.445%
5	2.239	288654	44317	0.833%
6	2.511	111973	18785	0.323%
7	3.055	122247	13739	0.353%
8	3.771	60638	8083	0.175%
9	4.124	1805661	208305	5.212%
10	5.041	31604573	293356	91.219%
In total		34646822	701191	100%

6. References

[1] Case, D. A. C., D.S.; Cheatham, T.E.; III; Darden, T.A.; Duke, R.E.; Giese, T.J.; Gohlke, H.; Goetz, A.W.; Greene, D.; Homeyer, N.; Izadi, S.; Kovalenko, A.; Lee, T.S.; LeGrand, S.; Li, P.; Lin, C.; Liu, J.; Luchko, T.; Luo, R.; Mermelstein, D.; Merz, K.M.; Monard, G.; Nguyen, H.; Omelyan, I.; Onufriev, A.; Pan, F.; Qi, R.; Roe, D.R.; Roitberg, A.; Sagui, C.; Simmerling, C.L.; Botello-Smith, W.M.; Swails, J.; Walker, R.C.; Wang, J.; Wolf, R.M.; Wu, X.; Xiao, L.; York D.M.; Kollman, P.A. *Amber16*, University of California: San Francisco, 2016.

[2] Frisch, M. J.; Trucks, G. W.; Schlegel, H. B.; Scuseria, G. E.; Robb, M. A.; Cheeseman, J. R.; Montgomery, J. A.; Vreven, T.; Kudin, K. N.; Burant, J. C.; Millam, J. M.; Iyengar, S. S.; Tomasi, J.; Barone, V.; Mennucci, B.; Cossi, M.; Scalmani, G.; Rega, N.; Petersson, G. A.; Nakatsuji, H.; Hada, M.; Ehara, M.; Toyota, K.; Fukuda, R.; Hasegawa, J.; Ishida, M.; Nakajima, T.; Honda, Y.; Kitao, O.; Nakai, O.; Klene, M.; Li, X.; Knox, J. E.; Hratchian, H. P.; Cross, J. B.; Bakken, V.; Adamo, C.; Jaramillo, J.; Gomperts, R.; Stratmann, R. E.; Yazyev, O.; Austin, A. J.; Cammi, R.; Pomelli, C.; Ochterski, J. W.; Ayala, P. Y.; Morokuma, K.; Voth, G. A.; Salvador, P.; Dannenberg, J. J.; Zakrzewski, V. G.; Dapprich, S.; Daniels, A. D.; Strain, M. C.; Farkas, O.; Malick, D. K.; Rabuck, A. D.; Raghavachari, K.; Foresman, J. B.; Ortiz, J. V.; Cui, Q.; Baboul, A. G.; Clifford, S.; Cioslowski, J.; Stefanov, B. B.; Liu, G.; Liashenko, A.; Piskorz, P.; Komaromi, I.; Martin, R. L.; Fox, D. J.; Keith, T.; Al-Laham, M. A.; Peng, C. Y.; Nanayakkara, A.; Challacombe, M.; Gill, P. M. W.; Johnson, B.; Chen, W.; Wong, M. W.; Gonzalez, C.; Pople, J. A. *Gaussian 03*, Revision E.01; Gaussian, Inc.: Pittsburgh PA, 2004.

[3] Stote, R. H.; Karplus, M., Zinc binding in proteins and solution: a simple but accurate nonbonded representation. *Proteins* **1995,** 23, (1), 12-31.

[4] Essmann, U. P., L.; Berkowitz, M. L.; Darden, T.; Lee, H.; Pedersen, L. G., A smooth particle mesh Ewald method. *J Chem Phys.* **1995,** 103, 8577-8593.

[5] Forester, T. R.; Smith, W., SHAKE, rattle, and roll: Efficient constraint algorithms for linked rigid bodies. *J Comput Chem* **2000,** 21, (2), 157.

[6] Hou, T. J.; Wang, J. M.; Li, Y. Y.; Wang, W., Assessing the Performance of the MM/PBSA and MM/GBSA Methods. 1. The Accuracy of Binding Free Energy Calculations Based on Molecular Dynamics Simulations. *J Chem Inf Model* **2011,** 51, (1), 69-82.
